# Spatially resolved multi-omics highlights cell-specific metabolic remodeling and interactions in gastric cancer

**DOI:** 10.1038/s41467-023-38360-5

**Published:** 2023-05-10

**Authors:** Chenglong Sun, Anqiang Wang, Yanhe Zhou, Panpan Chen, Xiangyi Wang, Jianpeng Huang, Jiamin Gao, Xiao Wang, Liebo Shu, Jiawei Lu, Wentao Dai, Zhaode Bu, Jiafu Ji, Jiuming He

**Affiliations:** 1 State Key Laboratory of Bioactive Substance and Function of Natural Medicines, Institute of Materia Medica, Chinese Academy of Medical Sciences and Peking Union Medical College, Beijing, 100050 China; 2 Key Laboratory for Applied Technology of Sophisticated Analytical Instruments of Shandong Province, Shandong Analysis and Test Center, Qilu University of Technology (Shandong Academy of Sciences), Jinan, 250014 China; 3 Key Laboratory for Natural Active Pharmaceutical Constituents Research in Universities of Shandong Province, School of Pharmaceutical Sciences, Qilu University of Technology (Shandong Academy of Sciences), Jinan, 250014 China; 4 Gastrointestinal Cancer Center, Key Laboratory of Carcinogenesis and Translational Research (Ministry of Education), Peking University Cancer Hospital and Institute, Beijing, 100142 China; 5 Shanghai Luming Biological Technology co.Ltd, Shanghai, 201102 China; 6 NHC Key Lab of Reproduction Regulation (Shanghai Institute for Biomedical and Pharmaceutical Technologies) & Shanghai Engineering Research Center of Pharmaceutical Translation, Fudan University, Shanghai, 200080 China; 7 Shanghai Key Laboratory of Gastric Neoplasms, Department of General Surgery, Shanghai Institute of Digestive Surgery, Ruijin Hospital, Shanghai Jiao Tong University School of Medicine, Shanghai, 200025 China; 8 NMPA Key Laboratory of safety research and evaluation of Innovative Drug, Institute of Materia Medica, Chinese Academy of Medical Sciences and Peking Union Medical College, Beijing, 100050 China

**Keywords:** Cancer metabolism, Metabolomics, Mass spectrometry

## Abstract

Mapping tumor metabolic remodeling and their spatial crosstalk with surrounding non-tumor cells can fundamentally improve our understanding of tumor biology, facilitates the designing of advanced therapeutic strategies. Here, we present an integration of mass spectrometry imaging-based spatial metabolomics and lipidomics with microarray-based spatial transcriptomics to hierarchically visualize the intratumor metabolic heterogeneity and cell metabolic interactions in same gastric cancer sample. Tumor-associated metabolic reprogramming is imaged at metabolic-transcriptional levels, and maker metabolites, lipids, genes are connected in metabolic pathways and colocalized in the heterogeneous cancer tissues. Integrated data from spatial multi-omics approaches coherently identify cell types and distributions within the complex tumor microenvironment, and an immune cell-dominated “tumor-normal interface” region where tumor cells contact adjacent tissues are characterized with distinct transcriptional signatures and significant immunometabolic alterations. Our approach for mapping tissue molecular architecture provides highly integrated picture of intratumor heterogeneity, and transform the understanding of cancer metabolism at systemic level.

## Abstract (web summary)

The spatial signature of metabolic remodeling in tumours remains to be explored. Here, the integration of mass spectrometry imaging-based spatial metabolomics and lipidomics with microarray-based spatial transcriptomics allows the visualisation of metabolic heterogeneity in gastric cancer.

## Introduction

Metabolic reprogramming was recognized as a core hallmark of tumor cells. A key feature of tumor cell metabolism is the ability to obtain nutrients from a frequently nutrient-poor microenvironment and utilize these nutrients to meet the demands of growth and proliferation^1,2^. In addition, the metabolic interaction between tumor and surrounding normal cells such as immune cells and stromal cells have profound effects on cancer progression and antitumor immune response^3,4^. Recent metabolomics and transcriptomics studies on distinct cancer tissues have revolutionized our understanding of tumor metabolism, paving ways to the development of novel strategies for tumor diagnosis and therapy^5^.

However, given the complexity of cellular metabolic networks, the heterogeneity of tumor microenvironment, and the diversity of intercellular metabolic communications, it is still challenging to comprehensively visualize the reprogrammed tumor metabolism and cell-cell interactions at multiple molecular levels. The development of mass spectrometry imaging (MSI) technique offers an insight approach to characterize the spatial signatures of metabolites and lipids in heterogeneous tumor tissues^6–8^. MSI-based spatially resolved metabolomics (SM) and spatially resolved lipidomics (SL) allow in situ screening of tumor initiation-, progression-, and metastasis-related metabolic biomarkers, thus allowing for the characterization of metabolic architecture of tumor and its surrounding microenvironment^9–14^. Spatially resolved transcriptomics (ST) was recently developed for the visualization and quantification of transcriptome with spatial resolution in individual tissue sections^15,16^. The introduction of ST into cancer tissue transcriptome-wide profiling has greatly revealed tumor metabolic mechanisms and cell-cell interactions in tumor-microenvironment at the transcriptional level^17–20^.

Gastric cancer is one of the most prevalent malignant diseases worldwide, with more than 1,080,000 new cases and over 760,000 deaths per year^21^. Metabolomics and transcriptomics exploitation on homogenized lesion tissue have advanced our understanding of gastric cancer biology, but the molecular informations regarding tissue architecture are lost during the course of sample pretreatment^22–24^. Single-cell RNA sequencing (scRNA-seq) on gastric cancer have enabled a better understanding of the transcriptional regulation of intratumoral heterogeneity and tumor-associated cellular reprogramming^25–28^, but the cell-specific metabolite and lipid alterations and interactions in gastric tumor microenvironment could not be acquired by scRNA-seq.

In this work, we propose an integrated spatially resolved multi-omics approach to explore the cell-specific metabolic remodeling and interactions in gastric cancer microenvironment. Adjacent frozen cancer tissue sections from gastric adenocarcinoma patients are prepared to perform SM, SL, and ST analysis, providing an atlas of spatially resolved metabolite, lipid, and gene expression patterns across tumor and surrounding normal cells. Identifying and imaging tumor-associated molecular alterations and cellular interactions of gastric cancer with its local microenvironment at both metabolic and transcriptional levels not only gives an insight into intratumor biochemical heterogeneity, but also helps to decipher the role of metabolic reprogramming in cancer growth and development.

## Results

### Spatially resolved multi-omics reveals intratumor heterogeneity

**Fig. 1:**
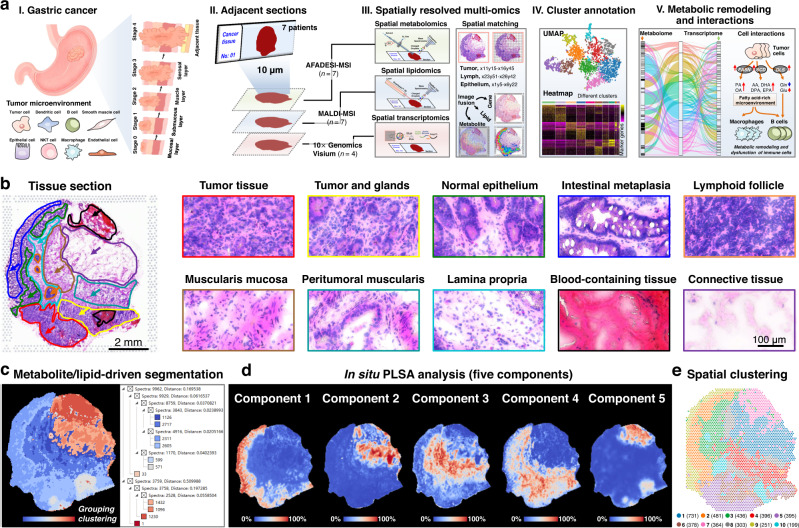
Spatially resolved multi-omics reveals intratumor heterogeneity of gastric cancer. **a** Strategy of integrated spatially resolved multi-omics for highlighting tumor metabolic remodeling and interactions. **b** H&E stain image of gastric cancer tissue section from patient “*No*.0429” and ×40 magnified H&E stain image of different gastric cancer tissue regions, scale bar = 2 mm for whole tissue section, scale bar = 100 μm for magnified images. The experiment was repeated three times. **c** Metabolite and lipid-driven tissue section segmentation based on the MALDI-MSI data. **d** Metabolite and lipid-driven in situ PLSA analysis. **e** Visium array spots colored by graph-based clustering algorithm.

With the goal of understanding intratumor metabolic remodeling and interactions in gastric tumor microenvironment, we proposed a spatially resolved multi-omics approach to integrate multilayer molecular information in heterogeneous tumor tissues (Fig. 1a). Gastric cancer tissues from seven individuals were cut into 10 μm frozen sections and then subjected to AFADESI-MSI based SM and MALDI-MSI based SL analysis, and four of them were used to perform 10× Genomics Visium-based ST analysis. For the integration of different omics data, the spatial resolution applied in this study was set to 100 μm. Fig. 1b illustrates the typical H&E images of gastric cancer tissue from patient *No*. 0429, and it suggests that in addition to tumor tissue, the gastric cancer sample also contains tumor and glandular mixed tissue, normal epithelium, intestinal metaplasia, lymphoid follicle, muscularis mucosa, peritumoral muscularis, lamina propria, blood-containing tissue, and connective tissue. Histology images of other representative gastric cancer samples also showed significant intratumor heterogeneity (Supplementary Fig. 1).

Data-driven tissue section segmentation map was built based on region-specific metabolite and lipid fingerprints, and different tissue regions with similar metabolite and lipid signatures were grouped together and given a specific color. The representative metabolite- and lipid-driven segmentation map of cancer tissue was shown in Fig. 1c. Different tissue regions in gastric cancer section exhibit obvious color diversity from cool blue to hot red, suggesting that there are significant differences in the spatial expression of metabolites and lipids. We further carried out unsupervised probabilistic latent semantic analysis (PLSA) based on the spatial expressions of tissue metabolites and lipids. All the detected variables were decomposed into five fundamental components to show the main spatial features of tissue metabolites and lipids (Fig. 1d). Component 1 represents metabolites and lipids that are significantly up-regulated in intestinal metaplasia and tumor tissues. Component 2 and component 3 characterize metabolites and lipids that are highly expressed in connective tissue and muscularis mucosa, respectively. Component 4 indicates a class of metabolites and lipids that are up-regulated in tumor and glandular mixed tissue. Metabolites and lipids that are specifically distributed in blood-containing tissue are decomposed as components 5. These results suggest significant intratumor metabolomic and lipidomic heterogeneity in gastric cancer tissue.

Furthermore, we explored the in situ transcriptional landscape and their heterogeneity in distinct regions of gastric cancer tissues by performing ST analysis. Four frozen cancer tissue sections which adjacent to the ones used for SM and SL were subjected to 10× Genomics Visium platform. Despite the limitation of sample analysis area, most tissue regions of gastric cancer tissue sections from different individuals could be contained within the 6.5 × 6.5 mm Visium array. Graph-based clustering algorithm was constructed to evaluate the intratumor transcriptomic heterogeneity in gastric cancer tissue. As shown in Fig. 1e, the whole cancer tissue section can be divided into ten different clusters based on underlying gene expressions. Moreover, it was found that the spatial patterns strongly recapitulated tissue histology. Cluster 1 and cluster 7 basically match the spatial features of connective tissue and the blood-containing tissue (Supplementary Fig. 3a, g). The spots in cluster 2 and cluster 9 are more distributed in normal epithelium and intestinal metaplasia (Supplementary Fig. 3b, h). Cluster 3 has obvious spatial correlation with lamina propria and muscularis mucosa (Supplementary Fig. 3c). The spots in cluster 4 and cluster 8 are mainly located in peritumoral muscularis (Supplementary Fig. 3d). Cluster 5 show significant spatial matching with tumor tissue, and cluster 6 exhibit obvious matching with tumor and glandular mixed tissue (Supplementary Fig. 3e, f). The spots in cluster 10 are more distributed in lymphoid follicle (Supplementary Fig. 3i).

### Region-specific molecule profiles in gastric cancer tissue

**Fig. 2:**
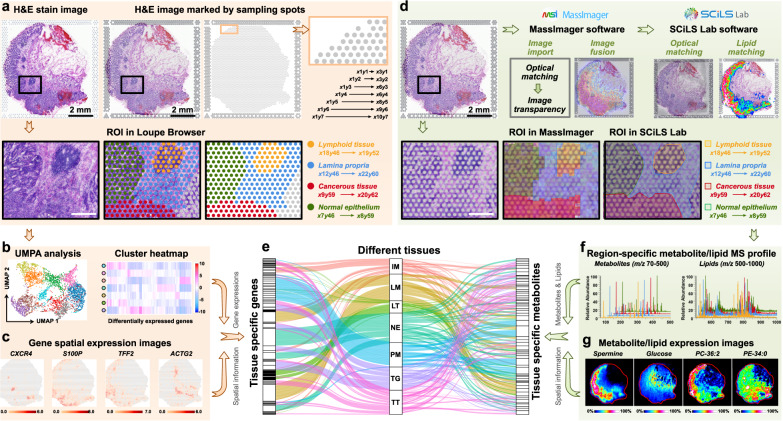
The extraction of gene, lipid, and metabolite profiles in different tumor micro-regions. **a** The process of extracting gene expression profiles in different tumor micro-regions of gastric cancer according to H&E stain image, scale bar = 2 mm for upper panels, scale bar = 500 μm for lower panels. The H&E stain experiment was repeated three times. **b** UMAP analysis and cluster heatmap of specifically expressed genes in different tumor micro-regions. **c** Spatial expression images of representative genes in gastric cancer tissue section (intensity in colour scale is log2 transformed). **d** The process of extracting metabolite and lipid profiles according to sampling spots-labeled H&E stain image, scale bar = 2 mm for upper panels, scale bar = 500 μm for lower panels. The H&E stain experiment was repeated three times. **e** Sankey diagram showing the distribution of marker genes and metabolites in different tissues. Each rectangle in the left represents a gene, each rectangle in the right represents a metabolite or lipid, each rectangle in the middle represents a tissue type, and the connection degree of each variable is showed based on the size of the rectangle. **f** Extracted region-specific metabolite and lipid profile. **g** MS images of representative metabolites and lipids in gastric cancer tissue section (intensity in colour scale is relative value). IM Intestinal metaplasia, LM Lamina propria, LT Lymphoid tissue, NE Normal epithelium, PM Peritumoral muscularis, TG Tumor and gland tissue, TT Tumor tissue.

Currently, SM, SL and ST analyses can only be performed separately on adjacent tumor sections. Tumor tissue is highly heterogeneous, so although the distance between adjacent sections is only 10 μm, different sections may still bring certain spatial errors in the process of section preparation and section transfer. 10× Genomics Visium-based ST enables point-by-point sampling and analysis of different micro-regions in H&E-stained tissue section. The sampling spots are distributed over tissue section with a spatial resolution of 100 μm. To more accurately match the spatial characteristics of SM, SL and ST data in tumor microenvironment, we labeled the ST sampling spots-labeled H&E images in this study. The coordinate of first spot in the upper left corner of H&E image was defined as *x*1*y*1, the spot with a horizontal interval of 100 μm is *x*2*y*1 and the spot with a vertical interval of 100 μm is *x*1*y*2. By analogy, we completed the definition of all sampling spots in H&E image (upper panel of Fig. 2a). Then, the gene expression profiles in different micro-regions of gastric cancer tissue can be extracted in a targeted manner using Loupe Browser software (lower panel of Fig. 2a). Furthermore, we can perform dimension reduction and cluster analysis based on tissue in situ gene expression signatures (Fig. 2b), and visualize the spatial features of differentially expressed genes (Fig. 2c).

Furthermore, we imported the sampling spots-labeled H&E image into AFADESI-MSI software MassImager and MALDI-MSI software SCiLS Lab for image fusion and spatial matching (upper panel of Fig. 2d and Supplementary Figs. 4, 5). Afterwards, the region-specific in situ AFADESI-MS and MALDI-MS profiles were extracted based on the sampling spots labeled-H&E image and used for spatial metabolomics and spatial lipidomics analysis (lower panel of Fig. 2d, f). Differentially expressed metabolites and lipids can be screened and imaged with spatial signatures (Fig. 2g). By this, region-specific metabolomic, lipidomic and transcriptomic fingerprints can be precisely extracted and visualized in highly heterogeneous gastric cancer tissue. Furthermore, we build a metabolome-transcriptome association network by integrating differentially expressed metabolites, lipids, genes and their spatial signatures. As shown in Fig. 2e, the spatial relationship between metabolites and gene expressions in tumor or surrounding normal tissues can be easily discovered through the metabolome-transcriptome association network.

### Region-specific metabolites, lipids, and gene expressions

Principal component analysis (PCA) model built based on the expressions of metabolites and lipids in different regions of gastric cancer tissue is shown in Supplementary Figure 6, and it indicates that different tissue micro-regions exhibit obvious clustering and grouping trends. The metabolite and lipid profile of tumor tissue is relatively close to that of intestinal metaplasia and epithelial tissue, and is quite different from that of connective tissue. AFADESI-MSI based SM platform exhibited better MS imaging performance for low-molecular-weight (*m/z* < 500) metabolites such as amino acids, polyamines, cholines, carnitines, organic acids, nucleotides, nucleosides, etc., with high sensitivity and low background interference. While, MALDI-MSI based SL platform showed better MS imaging performance for different kinds of lipids including fatty acids (FAs), phosphatidylcholines (PCs), phosphatidylethanolamines (PEs), ceramide-phosphates (CerPs), phosphatidylserines (PSs), phosphatidylglycerols (PGs), phosphatidylinositols (PIs), phosphatidic acids (PAs), sulfatide (SFTs), and lysophosphatides (LysoPLs). Supplementary Figs. 7, 8 show the spatial distributions of representative metabolites and lipids in gastric cancer tissue section imaged by AFADESI and MALDI, respectively. The combination of AFADESI-MSI and MALDI-MSI ensures high-coverage imaging of metabolites and lipids in different metabolic pathways of cellular metabolic networks. However, it should also be noted that some metabolites in the same metabolic pathway can be detected while some metabolites cannot be detected due to their low levels or the lack of easily ionized groups in their structure. Supplementary Fig. 9 demonstrates the numbers of imaged metabolites and lipids in key metabolic pathways including nucleotide metabolism, carbohydrate metabolism, lipid metabolism, amino acid metabolism, etc. Supplementary Figs. 10–35 illustrate the metabolites and lipids can be imaged in twenty-six specific metabolic pathways. Significantly, most metabolites and lipids exhibit highly heterogeneous spatial characteristics. Marker genes in different regions of gastric cancer tissue were screened and imaged in Supplementary Fig. 36.

### Dysregulated arginine and proline metabolism

**Fig. 3:**
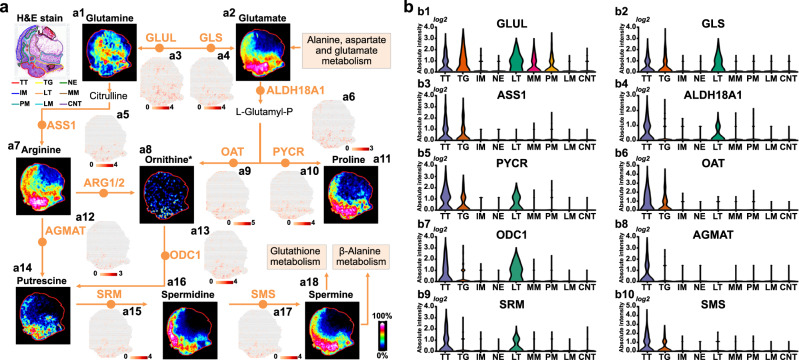
Visualization of reprogrammed arginine and proline metabolism pathway in gastric cancer. **a** MS images of key metabolites and spatial expression images of key genes in arginine and proline metabolism pathway (intensity in MS image colour scale is relative value, intensity in gene image colour scale is log2 transformed). **b** Violin plot show expression levels of key genes in arginine and proline metabolism pathway. *Ornithine only identified by high resolution MS spectrum. TT Tumor tissue, TG Tumor and gland tissue, NE Normal epithelium, IM Intestinal metaplasia, LT Lymphoid tissue, MM Muscularis mucosa, PM Peritumoral muscularis, LM Lamina propria, CNT Connective tissue.

Using the above strategy, we comprehensively investigated tumor-associated metabolic remodeling in gastric cancer. SM analysis suggested that arginine and proline all exhibit highly heterogeneous spatial distributions in gastric cancer tissues (Supplementary Fig. 37). Arginine plays significant roles in numerous metabolic pathways. Previous studies have demonstrated that arginine can modulate T cell metabolism and cell autophagy to influence tumor growth^29,30^. Proline and arginine are closely related in metabolic pathways, and it has been shown to be involved in the proliferation and invasion of tumor cells^31^. Here, we visualized key metabolites and regulatory genes in the entire arginine and proline metabolism pathways (Fig. 3a). Glutamine and glutamate are key components for the synthesis of arginine and proline, in which glutamine is down-regulated in tumor tissue, while the expression of glutamate was found to be up-regulated in tumor tissue (Fig. 3a1, a2). *GLUL* catalyzes the metabolism of glutamate to glutamine and is highly expressed in tumor tissue, lymphoid tissue and muscularis mucosa (Fig. 3a3, b1). *GLS*, in turn, can metabolize glutamine to glutamate. Gene expression image shows that *GLS* only highly expressed in tumor and lymphoid tissues (Fig. 3a4, b2). Both arginine and proline exhibit stronger expressions in tumor tissue, epithelium, and surrounding lymphoid tissues (Fig. 3a7, a11). Correspondingly, *ASS1*, *ALDH18A1* and *PYCR*, which regulate the synthesis of arginine and proline, also showed up-regulated expression in tumor and lymphoid tissues (Fig. 3a5, a6, a10, b3–b5). Arginine and proline can be further metabolized into putrescine, spermine, spermidine and other polyamines under the catalysis of *OAT*, *AGMAT*, *ODC1*, *SRM*, and *SMS*. Fig. 3a14, a16, a18 illustrate the spatial distributions of putrescine, spermine and spermidine in gastric cancer tissue section, and the results suggest that polyamines are mainly distributed in tumor tissues, followed by epithelium, intestinal metaplasia and lymphoid tissues. *OAT*, *AGMAT*, and *SMS* are only up-regulated in tumor tissue (Fig. 3a9, a12, a17, b6, b8, and b10), while *ODC1* and *SRM* are highly expressed in tumor and lymphoid tissues (Fig. 3a13, a15, b7, and b9). Overall, we found that the arginine and proline metabolism pathway was altered in gastric cancer, and the synthesis and metabolism of arginine and proline in tumor tissue were enhanced at both the metabolic and transcriptional levels.

### Significantly reprogrammed lipid synthesis and metabolism

**Fig. 4:**
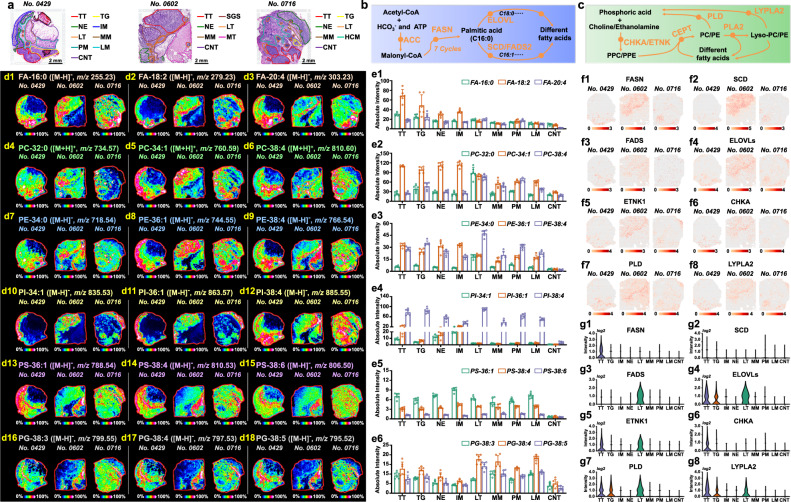
Visualization of reprogrammed lipid synthesis and metabolism pathways in gastric cancer. **a** H&E stain image of gastric cancer tissue section from patient “*No*.0429”, “*No*.0602”, and “*No*.0716”, scale bar = 2 mm. The experiment was repeated three times. **b** Fatty acid de novo synthesis pathway. **c** Synthesis and metabolism pathways of phosphatidylcholine and phosphatidylethanolamine. **d** MS images of representative lipids in gastric cancer tissues (intensity in colour scale is relative value). **e** Expression levels of representative lipids in different region spots of gastric cancer tissue from patient “*No*.0429” (seven tissue samples for spatial lipidomics, *n* = 6 independent section regions from patient “*No*.0429”, mean ± SD), *p*-values were calculated using the unpaired two-tailed t-test at confidence intervals 0.95. **f** Spatial expression images of key genes in lipid synthesis and metabolism pathways (intensity in colour scale is log2 transformed). **g** Expression levels of lipid metabolism-related key genes in different micro-regions of gastric cancer tissue from patient “*No*.0429”. TT Tumor tissue, TG Tumor and gland tissue, NE Normal epithelium, IM Intestinal metaplasia, LT Lymphoid tissue, MM Muscularis mucosa, PM Peritumoral muscularis, LM Lamina propria, CNT Connective tissue, SGS Serrated glandular structure, HCM Heterotopic cystic malformation.

Lipid metabolic reprogramming has been increasingly recognized as a hallmark of tumor cells^32^. As an important lipid, fatty acid (FA) plays indispensable roles in cell energy metabolism and cell signalling^33^. *ACC* and *FASN* catalyze the de novo synthesis of palmitic acid (FA-16:0), which can continue to be desaturated and elongated into different types of FAs under the catalysis of *SCD*, *FADS*, and *ELOVL* (Fig. 4b). By integrating SL and ST approach, we investigated the alteration of FA synthetic pathway in heterogeneous gastric cancer tissues. Figs. 4d1–d3 and e1 illustrate the MS images and expression levels of representative FAs in different gastric cancer tissue regions (Supplementary Data 1). Elevated expressions of FA-16:0 was found in tumor tissues. However, FA-20:4 was not only highly expressed in tumor tissues, but also up-regulated in lymphoid tissue, suggesting that FA-20:4 may be related to the immune response of lymphoid tissue. Correspondingly, *FASN* showed elevated expressions in tumor tissues (Fig. 4f1, g1). *SCD* and *FADS* are responsible for the desaturation of FAs. *SCD* is highly expressed in tumor tissues (Fig. 4f2), while *FADS* is more expressed in lymphoid tissues (Fig. 4f3, g3). *ELOVL*, which promote the elongation of FA carbon chain, exhibited up-regulated expression in both tumor and lymphoid tissues (Fig. 4f4, g4). These help to explain why FA-16:0 was only up-regulated in tumor tissues, while polyunsaturated long-chain FAs, such as FA-20:4, FA-22:4 and FA-22:5, were up-regulated in both tumor and lymphoid tissues (Fig. 4e1 and Supplementary Fig. 38).

Phospholipids are fundamental building blocks for cellar membranes and are likely to be further involved in signaling events important for tumor growth and metastasis^34^. Here, the spatial signatures of different phospholipids include PC, PE, PI, PS, PG, PA, and LysoPL were investigated. Interestingly, we found that the levels of saturated PC and PE such as PC-32:0 and PE-34:0 in lymphoid tissue were much higher than those in other tissue regions (Fig. 4d4, d7, e2, e3), while monounsaturated PC-34:1 and PE-36:1 exhibited stronger expressions in tumor, epithelium, and intestinal metaplasia tissues (Fig. 4d5, d8, e2, e3). Polyunsaturated long-chain PC and PE such as PC-38:4 and PE-38:4 presented up-regulated expressions in both tumor and lymphoid tissues, which matched the spatial features of polyunsaturated long-chain FAs (Fig. 4d6, d9, e2, e3). PC and PE are generated by combining FAs to phosphocholine (PPC) and phosphoethanolamine (PPE) under the catalysis of *CHKA*, *ETNK*, and *CEPT1* genes, and they can also be metabolized to FAs, choline and ethanolamine by the catalysis of *PLD*, *PLA2*, and *LYPLA2* genes (Fig. 4c). Both *ETNK1* and *CHKA* genes were found to be up-regulated in tumor tissue, but *ETNK1* genes also showed elevated expression in lymphoid tissue (Fig. 4f5, f6, g5, g6). *PLD* and *LYPLA2* genes presented up-regulated expression in tumor and lymphoid tissues (Fig. 4f7, f8, g7, g8). The spatially resolved characterization of lipids and related genes indicate that gastric tumor and surrounding lymphoid tissues possess more active lipid synthesis and metabolism. Fig. 4d10–d18 illustrate the MS images of other representative phospholipids in gastric cancer tissues. Monounsaturated PI-34:1 and PI-36:1 exhibited stronger expressions in intestinal metaplasia, epithelium, and tumor tissues, but very low in muscularis mucosa, connective, and lymphoid tissues (Fig. 4d10, d11, e4). In contrast, polyunsaturated PIs such as PI-38:4 and PI-38:5 only showed low expression in connective tissue (Fig. 4d12, e4, and Supplementary Fig. 39). Except for the low levels in connective tissue, all PSs exhibited strong expression in other gastric cancer regions (Fig. 4d13–15, e5). For PGs, they are mainly distributed in lamina propria, muscularis mucosa, and lymphoid tissues, and their expressions are not enhanced in tumor and intestinal metaplasia regions (Fig. 4d16–18, e6).

### Stepwise metabolic reprogramming was imaged

**Fig. 5:**
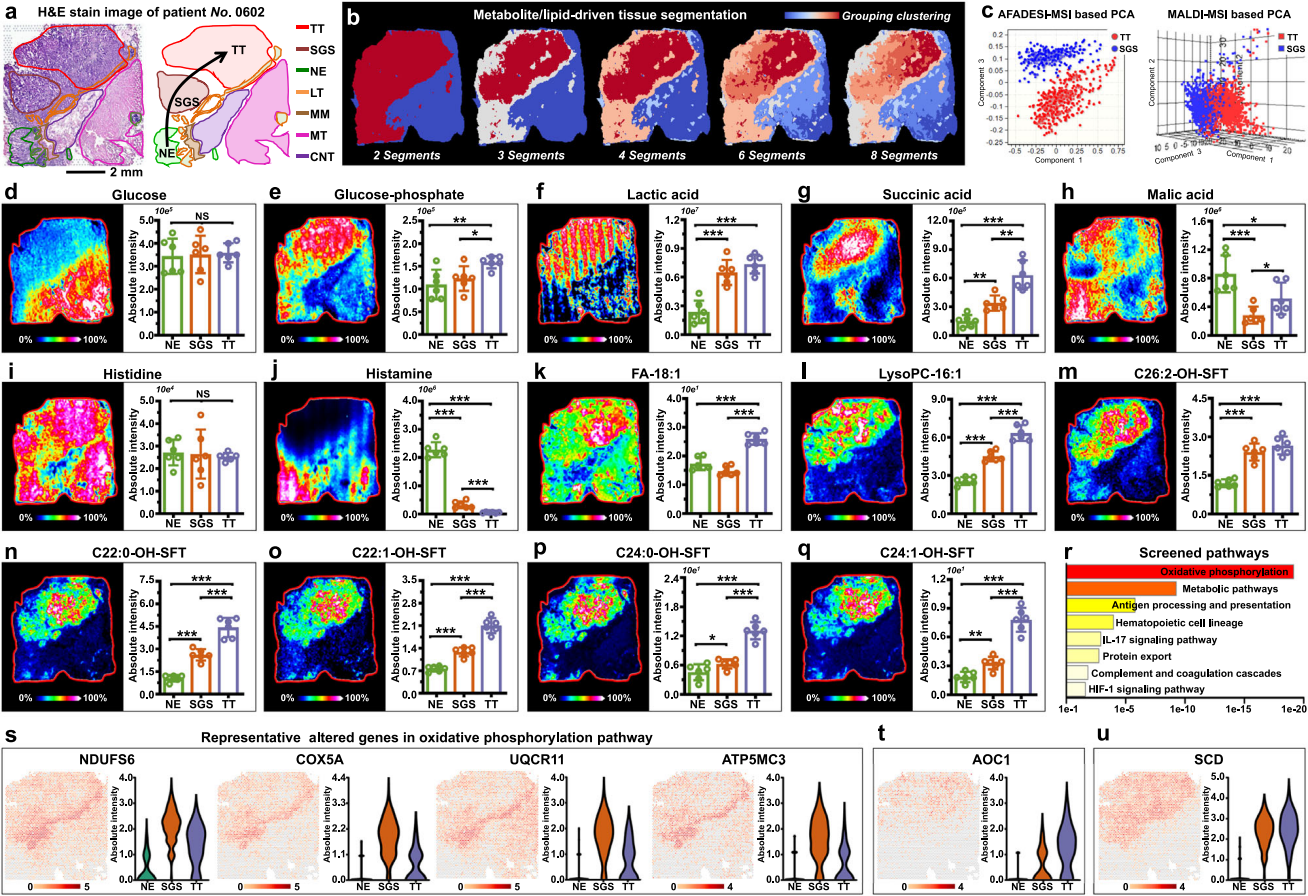
Visualization of stepwise metabolic reprogramming in gastric cancer. **a** H&E stain image of gastric cancer tissue section from patient “*No*.0602”, scale bar = 2 mm. The experiment was repeated three times. **b** Metabolite and lipid-driven tissue section segmentation. **c** PCA score plots based on AFADESI-MSI and MALDI-MSI data of tumor tissue (TT) and serrated glandular structure (SGS). **d**–**q** MS images and levels of glucose, glucose-phosphate, lactic acid, succinic acid, malic acid, histidine, histamine, FA-18:1, Lyso-PC-16:1, C26:2-OH-SFT, C22:0-OH-SFT, C22:1-OH-SFT, C24:0-OH-SFT, and C24:1-OH-SFT in different gastric cancer tissue section spots (seven tissue samples for spatial metabolomics and lipidomics, *n* = 6 independent section regions from patient “*No*.0602”, mean ± SD), ***p < 0.001, **p < 0.01, *p < 0.05, *p*-values were calculated using the unpaired two-tailed t test at confidence intervals 0.95, intensity in colour scale is relative value. **r** Pathways enriched in SGS tissue. **s** Representative altered genes in oxidative phosphorylation pathway, intensity in colour scale is log2 transformed. **t**, **u** Spatial expression images of *AOC1* and *SCD*, intensity in colour scale is log2 transformed. TT Tumor tissue, SGS Serrated glandular structure, NE Normal epithelium, LT Lymphoid tissue, MM Muscularis mucosa, MT Muscle tissue, CNT Connective tissue.

Patient *No*. 0602 was diagnosed with adenocarcinoma, but the histological image also revealed normal epithelium and serrated glandular structure. Gastric adenocarcinoma derived from the malignant hyperplasia of epithelium, and serrated glandular structure is a type of epithelium dysplasia^35^. The pathologic gradient alteration from epithelium to serrated lesion to tumor is indicated by the arrow in Fig. 5a. Coincidentally, metabolite- and lipid-driven segmentation map also showed gradient color alterations, indicating the stepwise metabolic reprogramming of gastric cancer (Fig. 5b).

Region-specific metabolite and lipid profiles of serrated lesion and tumor tissue were extracted to perform unsupervised PCA analysis, and the results showed striking differences between the two groups (Fig. 5c). Pathway-related discriminatory variables among epithelium, serrated lesion and tumor tissue were screened (Supplementary Data 2). Glucose is the direct energy source for cell metabolism. Although there was no expression difference in epithelium, serrated lesion and tumor tissue, it was significantly down-regulated compared with other normal tissues (Fig. 5d). Phosphorylation is an essential biochemical reaction for anaerobic glycolysis and aerobic oxidation of glucose. Fig. 5e shows the MS image of glucose-phosphate, and it suggests that the content of glucose-phosphate in tumor tissue is higher than that in epithelium and serrated lesion, and this corresponds to tumor cells tend to reprogram their metabolism to increase energy metabolism. The anaerobic glycolysis product lactic acid was found to exhibit higher expression in tumor and serrated lesion, suggesting that their anaerobic glycolysis reaction is more active than normal epithelium (Fig. 5f). Succinic acid and malic acid are key metabolites in Krebs cycle, succinic acid showed continuous up-regulation in epithelium, serrated lesion and tumor tissues (Fig. 5g), while malic acid presented down-regulated expression in serrated lesion and tumor tissue than that in normal epithelium (Fig. 5h). Histidine metabolism was screened as another abnormal metabolic pathway. Although the expression of histidine in different tissues did not show obvious different, its decarboxylation product, histamine, exhibited a dramatic decrease in serrated lesion and tumor, especially in tumor tissue (Fig. 5i, j). Unsaturated FA such as FA-18:1 and unsaturated lysophospholipids such as lysophosphatidylcholine (LysoPC)−16:1 were found to be differentially expressed in normal epithelium, serrated lesion and tumor tissues (Fig. 5k–l). Sulfatides (SFT) play significant roles in numerous biological processes including cell immune and tumor progression^36^. Interestingly, here we found that SFT such as C22:0-OH-SFT, C22:1-OH-SFT, C24:0-OH-SFT, C24:1-OH-SFT, and 26:2-OH-SFT all showed a stepwise up-regulation trend from epithelium to serrated lesion to tumor tissues (Fig. 5m–q).

Furthermore, to explore whether this stepwise metabolic alteration was affected by upstream gene, we extracted region-specific gene expression data to perform transcriptomic analysis. Differently expressed pathways were screened by KOBAS, and Fig. 5r illustrates the altered pathways that enriched in serrated lesion (Supplementary Data 3). Oxidative phosphorylation (OXPHOS) was the most dysregulated pathway, consistent with metabolite alterations in glucose phosphorylation, anaerobic glycolysis and aerobic oxidation found by SM. Spatial expressions of discriminatory genes in OXPHOS pathway were imaged, *NDUFS6*, *NDUFA6*, *NDUFAB1*, *NDUFB4*, *NDUFB3*, *COX5A*, *COX7B*, *COX7A2*, *UQCR11*, *UQCR10*, *UQCRQ*, *ATP5MC3*, *ATP5F1E*, and *ATP5PF* exhibited highest expressions in serrated lesion, followed by tumor and normal epithelium (Fig. 5s and Supplementary Fig. 40). Histidine metabolism, biosynthesis of unsaturated fatty acids, and tryptophan metabolism were screened as pathways that enriched in tumor tissue. The *AOC1* gene regulates the oxidation of histamine and its expression is highest in tumor tissue, followed by serrated lesion and lowest in normal epithelium (Fig. 5t). Immunohistochemical staining of *AOC1* protein on adjacent cancer section were coincident with the *AOC1* gene expression image (Supplementary Fig. 41). Further combining with the spatial feature of histamine (Fig. 5i), we can speculate that the down-regulation of histamine in tumor tissue is caused by its excessive oxidation. *SCD* is a key gene in the biosynthesis of unsaturated FA, and it was found to be significantly up-regulated in serrated lesion and tumor tissue (Fig. 5u). This is consistent with the changing trend of unsaturated FAs and some lipids containing unsaturated FA side chains (Fig. 5k–l).

### Immunometabolic reprogramming in tumor interface region

**Fig. 6:**
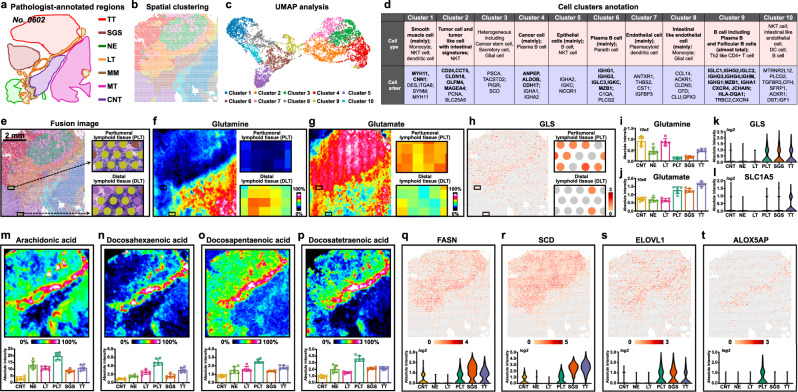
Imaging the immunometabolic reprogramming in tumor interface region of gastric cancer. **a** Pathologist-annotated regions of cancer tissue section from patient “*No*.0602”. **b** Visium array spots colored by graph-based clustering algorithm. **c** UMAP plot of gastric cancer tissue colored by clusters. **d** Cell cluster annotation of gastric cancer tissue. **e** The fusion image of visium array spots and H&E stain image, scale bar = 2 mm. The H&E stain experiment was repeated three times. **f**, **g** MS images of glutamine and glutamate in whole gastric cancer tissue section and in lymphoid tissue regions, intensity in colour scale is relative value. **h** Spatial expression images of *GLS* in whole gastric cancer tissue section and in lymphoid tissue regions, intensity in colour scale is log2 transformed. **i**–**l** Expression levels of glutamine, glutamate, *GLS* genes and SLC1A5 in different gastric cancer tissue section spots (seven tissue samples for spatial metabolomics, *n* = 6 independent section regions from patient “*No*.0602”, mean ± SD), *p*-values were calculated using the unpaired two-tailed t-test at confidence intervals 0.95. **m**–**p** MS images and expression levels of arachidonic acid, docosahexaenoic acid, docosapentaenoic acid, and docosatetraenoic acid in different gastric cancer tissue section spots (seven tissue samples for spatial lipidomics, *n* = 6 independent section regions per tissue sample, mean ± SD), intensity in colour scale is relative value. **q**–**t** Spatial images and expression levels of *FASN*, *SCD*, *ELOVL1*, and *ALOX5AP* in gastric cancer tissue, intensity in colour scale is log2 transformed. TT Tumor tissue, SGS Serrated glandular structure, NE Normal epithelium, LT Lymphoid tissue, PLT Peritumoral lymphoid tissue, MM Muscularis mucosa, MT Muscle tissue, CNT Connective tissue.

The spatial transcriptome landscape of tumor and neighboring tissues were explored and a total of ten distinct clusters were identified. Significantly, when we projected the cluster assignments back onto tissue section coordinates, we discovered a distinct “interface” cluster (cluster 9) at the junction of tumor and neighboring tissues (Fig. 6b). UMAP plots show that the “interface” cluster 9 is also located at the middle transition region in UMAP-space, with normal and normal-like cluster 1, cluster 5, cluster 6, cluster 8, cluster 10 on the left, tumor and tumor-like cluster 2, cluster 3, cluster 4, cluster 7 on the right (Fig. 6c).

To better understand the composition and biological representation of gene expression-driven clusters, we annotated these cell clusters with their dominant gene markers. The detail annotations of these clusters were demonstrated in Fig. 6d (Supplementary Data 4). Strong concordance was observed between pathologist-annotated region and cell cluster annotation. For example, cluster 3, cluster 4, and cluster 7 were identified as tumor tissues according to pathologist’s annotations. Cell annotation results showed that cluster 3 is heterogeneous including cancer stem cell, secretory cell, and glial cell, while cluster 4 is mainly composed of cancer cells, cluster 7 contained endothelial cells and few plasmacytoid dendritic cells. Cluster 2 exhibits some gene signatures of intestinal metaplasia with array spots mainly located in the serrated lesion regions. Coincidentally, serrated lesion mostly occurs in colorectum. Most notably, although only very few amounts of tumor-infiltrating immune cells were identified in tumor tissue, we discovered that “interface” cluster 9 exhibits significant immune and inflammation-related signatures with plenty of plasma B cell, follicular B cell, and Th2-like CD4 + T cells. The pathologist re-examined cluster 9 region and found that it contained some lymphoid tissue. To validate plasma B cell and follicular B cells in cluster 9, we explored the concordance between cell annotation and staining of the corresponding proteins within the cancer section. Immunohistochemical staining of CD 20 and CD 38 on adjacent cancer sections were coincident with the defined plasma B cell- and follicular B cell-enriched cluster 9 region (Supplementary Fig. 42). Located at tumor boundary where the tumor contacts neighboring tissues, the immune and inflammation-related cluster 9 is believed to be important for tumor cell invasion and progression.

Accumulating evidence suggest that tumor cells can reshape their metabolism to create a microenvironment suitable for tumor growth and suppress the immune surveillance system^37,38^. In immune and inflammation-related cluster 9, we found two lymphoid tissues with different distances from tumor boundary, the one adjacent to tumor cells was defined as peritumoral lymphoid tissue (PLT, Fig. 6e), and the one adjacent to normal epithelium was classified as distal lymphoid tissue (DLT, Fig. 6e). SM and SL analysis indicate that PLT undergoes remarkable metabolic reprogramming. For instance, glutamine plays energy-generating and biosynthetic roles in growing cells, and many tumor cell lines display glutamine addiction^39^, this phenomenon was further confirmed here in gastric cancer (Fig. 6f). More interestingly, we found a dramatic decrease in glutamine levels in PLT compared to DLT, suggesting that glutamine is also reprogrammed in PLT (Fig. 6f, i, Supplementary Data 5). SLC transporters represented by the heavily studied *SLC1A5* are essential for pushing glutamine into the cells, and then glutamine was converted to glutamate by glutaminase (*GLS*). In contrast to glutamine, the content of glutamate in PLT was significantly higher than that in DLT (Fig. 6g, j). Meanwhile, we discovered *GLS* gene and glutamine transporter *SLC1A5* exhibited higher expression in PLT (Fig. 6h, k, l). These data indicate that glutamine uptake and metabolism is up-regulated in PLT.

FAs are indispensable for tumor energy metabolism and signaling^33^. Here, we also found that FAs, especially long-chain unsaturated FAs such as arachidonic acid, docosahexaenoic acid, docosapentaenoic acid, and docosatetraenoic acid were also significantly up-regulated in PLT (Fig. 6m–p). Previous studies suggest that the increased synthesis of FAs in tumor cells will generate a FA-rich microenvironment, which affects the biofunctions of immune cells^40–42^. Following ST analysis showed that FA synthesis-related *FASN*, *SCD* and *ELOVL* genes were also up-regulated in PLT (Figs. 6q–s). Arachidonic acid can be metabolized to prostaglandins, leukotriene, and lipoxin, involved in chronic inflammation^43^. The *ALOX5AP* gene promotes the metabolism of arachidonic acid through 5-lipoxygenase pathway to generate leukotriene inflammatory mediators, and its expression in PLT is much higher than that in other tissues (Fig. 6t). Docosahexaenoic acid was proved to be substrate for the biosynthesis of anti-inflammatory proresolving endogenous mediators^43^. The upregulation of these long-chain polyunsaturated FAs in PLT matches the biological features of immune and inflammation-related cluster 9, and also indicates that PLT exhibited an enhanced inflammatory response than DLT to inhibit the proliferation of tumor cells.

## Discussion

Tumor cells can reprogram the regulatory and functional properties of their metabolism to support the synthesis of building blocks and energy components required for cancer development. Understanding how reprogrammed metabolic networks affect tumor growth holds the key for identifying potential metabolic vulnerabilities for better cancer treatment^44,45^. However, tumor tissues often exhibit significant molecular heterogeneity, and metabolic communications between tumor and surrounding normal cells are critical for tumor growth, proliferation, and metastasis^46–48^. MSI enables in situ analysis of metabolites and lipids in heterogeneous cancer tissues while retaining spatial information, and thus allows for spatially resolved mapping of cancer-associated metabolite and lipid changes^49,50^. In this study, we performed MSI-based SM and SL analysis and 10× Genomics Visium-based ST sequencing on gastric cancer tissues. Tumor-, epithelium-, intestinal metaplasia-, lymphoid follicle-, muscularis mucosa-, lamina propria-, serrated lesion-, and connective tissue-specific metabolic profiles and gene expressions can be accurately extracted for dimension reduction analysis and discriminatory variable screening. The integration of SM, SL, and ST adds extra value for exploring tumor metabolism: (i) metabolites and lipids are interconnected in metabolic networks, and the combined SM and SL profiling provide a more comprehensive picture of tumor metabolism; (ii) the expressions of metabolites and lipids are regulated by upstream genes, and the introduction of ST can help us visualize complex tumor metabolic reprogramming at multiple interrelated levels.

However, the fact that more than 40,000 metabolites are distributed in the cellular metabolic network with significant content variation, which makes their in situ MSI challenging^51^. AFADESI-MSI was developed for high-sensitive tissue metabolites imaging by our group, and the spatial features of over 1500 metabolites such as amino acids, polyamines, carnitines, cholines, nucleosides, nucleotides, nitrogen bases, organic acids, carbohydrates, etc. in biological tissues were successfully mapped use this technique^9,52^. MALDI-MSI is the most commonly used MSI technique, which is more suitable for the imaging of lipids ^7,53,54^. Here, we carried out AFADESI-MS and MALDI-MS imaging analysis on adjacent gastric cancer tissue sections, respectively, to ensure high-coverage visualization of metabolites and lipids. The imaged metabolites are extensively distributed in cancer-associated carbohydrate metabolism, energy metabolism, lipid metabolism, and amino acid metabolism pathways, and thus allows for a comprehensive and deep exploring of altered metabolites in different metabolic pathways.

Arginine and proline metabolism was found to be significantly reprogrammed in gastric cancer. Both arginine and its synthesis-related *ASS1* gene are up-regulated in tumor tissues (Fig. 3a5, a7, b3). The elevated arginine in gastric cancer cells can be used for nitric oxide, protein, and nucleotides synthesis, and as a direct activator of *mTOR*^55,56^. Given the indispensable roles of arginine plays in cancer growth, arginine deprivation through targeting arginine deiminase or arginase has been developed as an effective approach for cancer therapy^57,58^. Moreover, it should be noted that arginine is a key precursor metabolite for cellular polyamine synthesis. An important feature of cancer cell is the ability to upregulate the expressions of polyamines to promote tumor growth, invasion and metastasis^59^. And this was further confirmed in our study, as shown in Fig. 3a16, a18, polyamines including spermine and spermidine exhibit significant up-regulation in gastric cancer cells. *ODC1* gene, which catalyzes the first step of polyamine biosynthesis, also showed elevated expression in gastric cancer tissues (Fig. 3a13, b7). Suggestive evidence for *ODC1* can be induced during carcinogenesis by a variety of oncogenic stimuli has been provided by earlier observations^60,61^. Therefore, we speculate that the up-regulated expressions of polyamines in gastric cancer should be attributed to the enhancement of polyamine synthesis.

In the tumor microenvironment, glutamine is indispensable for both tumor cells and immune cells. Targeting glutamine metabolism or blocking glutamine were proved to be effective approaches for inhibiting tumor growth^62,63^. Here, we found that glutamine is overutilized within the tumor tissue, and its content also exhibited a dramatic decrease in peritumoral lymphoid tissue (Fig. 6f, i). Given the key influences of glutamine on immune cells such as regulate macrophage polarization and active effector T cell to exert antitumor function^64,65^, we speculate that the low levels of glutamine in tumor and peritumoral lymphoid tissues should be an important reason for the inability of immune cells to inhibit the growth and proliferation of gastric cancer. Histamine is an important inflammatory biogenic amine and is also recognized as a main mediator of cell proliferation^66^. SM analysis showed that histamine was significantly down-regulated in gastric tumor tissues (Fig. 5j). However, elevated levels of histamine have been discovered in other tumors including colon, melanoma, and breast cancer^67,68^. Growing evidence suggests that histamine involved in the modulation of immune responses such as enhancing T helper type 1 responses and promoting myeloid cell differentiation to suppress cancer formation^69,70^. Coincidentally, we identified very few infiltrating immune cells in the analyzed gastric cancer tissue sections. The low expression of histamine and the lack of infiltrating immune cells in the tumor microenvironment may be another reason why the immune system fails to suppress gastric cancer growth.

Lipid reprogramming was recognized as a prominent metabolic alteration in tumor. To support the synthesis of biological membranes and to generate energy components, tumor cells usually increase their lipogenesis and FA oxidation^32,71,72^. In this study, we carried out SL analysis and imaged the spatial alterations of different lipids including FA, PC, PE, PS, PG, PI, PA, ST, CerP, LysoPL, and cholesterol in gastric cancer tissues. In general, we found that lipids showed the following spatial characteristics: (i) except for PS and PI, most lipids exhibited elevated expressions in tumor tissues; (ii) some lipids with short FA chains presented totally different spatial signatures with those contained polyunsaturated FA chains, such as FA, PC, PE, and PI (Fig. 4d, e). Following ST analysis revealed that *FASN*, *SCD*, *FADS* and *ELOVL* genes which catalyze the synthesis, elongation, and desaturation of FA; *CHKA* and *ETNK1* genes which regulate phospholipid synthesis; *PLD* and *LYPLA2* genes which control lipid metabolism; *CPT1A* and *CRAT* genes which catalyze the β-oxidation of FA all showed up-regulated expressions in tumor tissues (Fig. 4f, g, and Supplementary Fig. 43). Transcription factors sterol regulatory element-binding proteins (*SREBPs*) coordinate genes for lipid synthesis. *SREBP1* (encoded by *SREBF1*) mainly controls genes involved in the FA synthesis pathway such as *FASN*, *SCD*, *FADS*, and *ELOVL*, while *SREBP2* (encoded by *SREBF2*) tends to regulate genes in the cholesterol biosynthesis pathway such as *ACAT2*, *HMGCS1*, and *HMGCR*^34,73,74^. Here, we found *SREBF1* and *SREBF2* genes also exhibited increased expressions in tumor tissues (Supplementary Fig. 44). Spatially resolved characterization of up-regulated lipids, lipid synthesis-related enzyme genes, and transcriptional regulator genes in gastric cancer once again proves that tumor cells have to reshape the regulatory and functional properties of their lipid synthesis and metabolism pathways to maintain cell activity.

It’s also worth noting that tumor-associated lipid alterations strongly impact the differentiation and activation of immune cells, and the immune cells can also reprogram their lipid metabolism to inhibit tumor growth^75–77^. Here, only paucity of T cells was discovered in the parenchyma and stroma of gastric cancer tissues, which can be characterized as an immune-desert phenotype. However, we found a thin cluster rich in Plasma B cell, Follicular B cell, and Th2-like CD4 + T cell at the interface of tumor parenchyma and neighboring tissues, and this cluster also included plenty lymphoid tissue (Fig. 6a–e). In addition, we identified significant lipid alterations in lymphoid tissue at the interface regions. Compared with distal lymphoid tissue, the expressions of FA de novo synthesis-related *FASN*, *SCD*, *ELOVL* genes are up-regulated in interfacing lymphoid tissue (Fig. 6q–s). Moreover, the levels of long-chain polyunsaturated FAs such as arachidonic acid, docosahexaenoic acid, docosapentaenoic acid, and docosatetraenoic acid were found to be dramatically up-regulated in interfacing lymphoid tissue, even higher than those in tumor tissues (Fig. 6m–p). And, this consistent with previous report that the accumulation of long-chain FAs in immune cells and tumor microenvironment drives dysfunction of immune cells, leading to the immunosuppression of tumor^41^. In addition to FAs, several studies have shown that cholesterol is enriched in the tumor microenvironment, and cholesterol can inhibit T cell differentiation and induce T cell functional exhaustion^78–80^. Here, our results indicate that the level of cholesterol sulfate were increased in the tumor parenchyma and epithelium of gastric cancer (Supplementary Fig. 45). Moreover, cholesterol synthesizing enzyme genes *HMGCS1* and *HMGCR*, as well as *SREBF2* genes that regulate genes in cholesterol synthesis pathway, all were elevated in tumor and interfacing lymphoid tissues (Supplementary Figs. 44, 46). We speculated that the upregulation of FA and cholesterol synthesis at transcriptional level, and the accumulation of long-chain polyunsaturated FAs and cholesterol at lipid level should be one of the reasons why immune cells could not effectively inhibit the growth of gastric cancer.

In conclusion, our study shows how SM, SL, and ST approach can be integrated for characterizing the complex tumor metabolic remodeling and tumor-microenvironment metabolic interactions in highly heterogeneous cancer tissues. Metabolite, lipid, and gene expression signatures and their spatial alterations in tumor microenvironment were precisely imaged and further linked in distinct metabolic pathways. Insights into cancer-associated metabolic dependencies and immunometabolic alterations emerged from our analysis not only help to better understand the molecular mechanisms of tumor, but also provide potential vulnerabilities that could be targeted for cancer therapy.

## Methods

### Ethics approval

This research complies with all relevant ethical regulations. The study of human tumor samples was performed according to the Declaration of Helsinki and Good Clinical Practice and approved by the Ethnical Committee of Peking Cancer Hospital (grant no. 2022KT87). Informed written consent was obtained from all participants. The animal experiments were conducted with the approval of the Animal Ethical Committee at the Institute of Materia Medica, Chinese Academy of Medical Science, and Peking Union Medical College (grant no. 00007751).

### Human gastric cancer tissue specimen

Postoperative cancer tissue from seven male patients diagnosed with gastric cancer and underwent surgery were included in this study. The age distribution is 50–89. All the tumor classification of these seven patients is adenocarcinoma. And all patients did not undergo chemotherapy or radiotherapy before the surgery. The collected cancer tissues were immediately placed in dry ice and then transferred to a −80 °C refrigerator. This study is mainly focused on the development of integrated spatial multi-omics analysis method and the exploration of tumor metabolic remolding, so no gender-based analyses have been performed.

### Xenograft tumor specimen

Xenograft model in nude mice with human gastric cancer SGC7901 cells were built. NPG mice (female, 6 weeks, 18–20 g, stock no. SCXK2019-0002) were obtained from Beijing Vitalstar Biotechnology Co.,Ltd. Mice were kept on a 12 h/12 h light/dark cycle with the temperature at 20–24 °C and humidity at 40–70%. The experimental protocols (00007751) were approved by the Animal Ethical Committee at the Institute of Materia Medica, Chinese Academy of Medical Science, and Peking Union Medical College. The care and use of animals complied with the Guide for the Care and Use of Laboratory Animals published by the National Institutes of Health. Anesthesia was given before tumor implantation subcutaneously. Seven, thirteen, and twenty-one days after the transplantation of cancer cells, mice were euthanized by CO_2_ inhalation and tumors were harvested for mass spectrometry imaging analysis. Our animal protocol sets the maximal tumor size at 1.5 cm diameter. In these studies, all the tumor size was not exceeded 1.5 cm in diameter.

### Preparation and processing of cancer tissue section

The postoperative gastric cancer tissues were embedded in OTC and cut into 10 μm serial frozen sections at −20 °C on a cryostat microtome (Leica CM 1860 UV). Two sets of tissue sections were mounted onto SUPERFROST PLUS slides (Thermo) for AFADESI-MSI. Two sets of tissue sections were mounted onto indium tin oxide (ITO)-coated glass slides for MALDI-MSI analysis. One set of tissue sections were stained with hematoxylin and eosin and evaluated by pathologists. The pathologists evaluated the cellular composition and heterogeneity of the tissue sections, and then selected a 6.5 × 6.5 mm area with the most significant tumor heterogeneity from the entire tissue section. Then, we cut the original tissue sample according to the rectangular area delineated by the pathologist, and only the regions with significant tumor heterogeneity were retained. New adjacent tissue sections were prepared on retained cancer tissues and mounted onto 10 × Genomics Visium array slides for spatially resolved transcriptomics analysis. Before AFADESI-MSI and MALDI-MSI analysis, the tissue sections were dried in vacuum for about 15 min.

### AFADESI-MSI

Custom-built AFADESI-MSI platform equipped with Q-Exactive Orbitrap mass spectrometer (Thermo Scientific, Bremen, Germany) and AFADESI ion source was used for tissue metabolites imaging. The experiment was carried out in both positive and negative ion modes at *m/z* 70–1000. The spray solvent used in this study was acetonitrile and water (80:20, *v*/*v*), and the flow rate of spray solvent was set to 5.0 μL/min. Sprayer voltages were set at 4500 V and −4500 V in positive and negative ion mode, respectively. The extracting gas flow of AFADESI ion source was 45 L/min. The flow rate of nebulizing gas (N_2_) was set to 0.7 MPa. Imaging analysis was performed by continuously scanning the tissue section in *x*-direction at 100 μm/sec, separated by a 100 μm vertical step in *y*-direction. MassImager Pro^TM^ software was used for background subtraction, image reconstruction, and the calculation of average metabolite expressions in region of interest^81^.

### Matrix coating and MALDI-MSI

1,5-Diaminonaphthalene, 2.0 mg/mL in acetonitrile and water (70:30, *v*/*v*), were used as MALDI matrix for tissue MALDI-MSI. Eight cycles of MALDI matrix were sprayed onto cancer tissue sections with a flow rate of 75 μL/min by using a HTX TM-Sprayer^TM^ (HTX Technologies, Carrboro, NC). The pray gas pressure and spray temperature were set to10 psi and 55 °C, respectively. Track speed and spacing of the sprayer were set to 800 mm/min and 3 mm, respectively. After spraying matrix, the tissue sections were subjected to rapifleX^TM^ MALDI TOF/TOF MS (Bruker Daltonics, Billerica, MA) for MALDI-MSI analysis. The *m/z* scan rang was set to 80–1000 in both positive and negative ion modes. The spatial resolution of MALDI imaging was set to 50 μm. The MS images were viewed and processed using SCiLS Lab 2018b software (GmbH, Bremen, Germany).

### Spatially resolved transcriptomics analysis

All reagents were obtained from Visium Spatial Gene Expression Reagent Kits, and the experiments were operated according to the user guide of “Visium Spatial Protocols-Tissue Preparation Guide (CG000240)”, “Visium Spatial Gene Expression Reagent Kits-Tissue Optimization (CG000238)”, and “Visium Spatial Gene Expression Reagent Kits (CG000239)”

#### Sample preparation

Tissue samples were prepared and processed based on the User Guide of Visium Spatial Gene Expression. Frozen tissue sections (10 μm, adjacent to the ones being analyzed by AFADESI-MSI and MALDI-MSI) were mounted onto 10×Genomics Visium array slides. Then, the frozen tissue sections were dehydrated with isopropanol for 1.0 min, fixed in methanol for 1.0 min and stained with hematoxylin and eosin. Next, the slides were mounted in 80% glycerol and brightfield images were taken on 3D HISTECH Pannoramic MIDI FL, whole-slide scanner at 40× resolution.

#### ST barcoded microarray slide information

Library preparation slides were purchased from the Spatial Transcriptomics team (https://www.10xgenomics.com/). The diameter of the array spots is 55 μm, and the distance between adjacent array spots is 100 μm, covering an area of 6.5 × 6.5 mm^2^. Each slide includes four capture areas, each with about 5000 unique gene expression spots.

#### Tissue optimization

Adjacent frozen cancer tissue sections on 10× Genomics Visium array slides were fixed, stained, and permeabilized for different times. Poly adenylated mRNA from the attached tissue section were captured by probes on the slides. Then, add Master Mix containing reverse transcription (RT) reagents and fluorescently labeled nucleotides to the surface of the tissue section to obtain fluorescently labeled cDNA. Next, remove excess tissue, leaving fluorescently labeled cDNA covalently linked to oligonucleotides on the Visium array slides. Then, fluorescently labeled cDNA is visualized. The permeabilization time that results in maximum fluorescence signal with the lowest signal diffusion is optimal. If the signal is the same at two time points, the longer permeabilization time was selected.

#### On-slide tissue permeabilization, cDNA synthesis, library construction and sequencing

Fixed and stained tissue sections were permeabilized using permeabilization enzyme. Then, the primers on the spots capture the poly-adenylated mRNA released from the overlying cells. Add RT reagents on permeabilized tissue sections and incubate to produce spatially barcoded, full-length cDNA from poly-adenylated mRNA on the slide. Second strand cDNA synthesis Mix regent was added on the tissue surface for second strand synthesis, and this followed by denaturation and transfer of the cDNA from capture area to a corresponding tube for amplification and library construction. After transfer of cDNA from the slide, spatially barcoded, full-length cDNA is amplified by PCR to generate sufficient mass for library construction. Then, P5, P7, i7, and i5 sample indexes, and TruSeq Read 2 are added via End Repair, A-tailing, Adaptor Ligation, and PCR. The final libraries contain the P5 and P7 primers used in Illumina amplification. TruSeq Read 1 is used for priming and sequencing the 16 bp Spatial Barcode and 12 bp UMI, and TruSeq Read 2 is used for priming and sequencing the cDNA insert. The two 10 bp sample indexes are sequenced in the i5 and i7 read respectively.

#### Data analysis

Loupe Browser 6 software was used to visualize the spatial signatures of transcriptomics data, to perform dimensionality reduction analysis, and to screen differentially expressed genes. Tissue annotation and multi-omics clustering were performed independently. Graph-based clustering analysis was performed to find highly-connected “modules” in the graph according to the tissue in situ gene expression profiles, and the spots with similar gene expressions in the graph are given the same color. Uniform manifold approximation and projection (UMAP) analysis was performed to visualize the cell ranger among different tissue regions. Significant features tool was used to find features expressed highly with groups, relative to other checked groups in the selected category.

### Spatially resolved metabolomics analysis

10× Genomics Visium sampling spots-labeled H&E images were imported into MassImager Pro^TM^ software for image fusion and spatial matching. After background subtraction, region-specific mass spectrometry spectra of different tissue regions were extracted using “arbitrary region tool” in MassImager Pro^TM^ software. The separated sample dataset matrixes were then imported into the Markerview^TM^ software 1.2.1 (AB SCIEX) for peak picking, peak alignment and isotope removing (process spectra options: the mass tolerance was 0.01 Da). The dataset matrixes were then further imported into SIMCA-P 14.0 software package (Umetrics AB, Umeå, Sweden) for multivariate statistical data analyses. Discriminating metabolites can be screened both from MassImager Pro^TM^ and SIMCA-P 14.0 software^81^. *P*-values per metabolite between two groups were calculated by unpaired two-tailed t-test at confidence intervals 0.95.

### Spatially resolved lipidomics analysis

10× Genomics Visium sampling spots-labeled H&E images were imported into SCiLS Lab 2018b software. Then, region-specific mass spectrometry spectra were extracted using “create new polygonal region button” in SCiLS Lab. Region-specific mass spectrometry spectra based principal component analysis (PCA) was carried out to explore the global changes of lipids in different tissue microregions and screen region-specific lipids. Data-driven segmentation analysis was performed based on the mass spectrometry spectra of each image pixels, and the results can be used to evaluate lipid expression similarity between different tissue image pixels. Probabilistic latent semantic analysis (PLSA) was performed based on the in situ mass spectrometry spectra, and the results can be interpreted as spatial tissue components and their corresponding mass distribution in the tissue component.

### Analyte identification

The ions of interest were first extracted to compared with in-house database (LuMet-animal database, about 2000 standards of endogenous metabolites built by Shanghai Luming Biotechnology Co., LTD), HMDB, METLIN, and LIPID MAPS databases using exact molecular weights and a mass accuracy of less than 5 ppm (Q-Exactive Orbitrap mass spectrometer) or 10 ppm (rapifleX^TM^ MALDI TOF/TOF mass spectrometer). Then, the ions were extracted to perform high resolution MS/MS analysis direct on tissue sections. For those ions with very low levels for which on-tissue MS/MS spectra could not be obtained, we made tissue homogenates and analyzed them by LC-MS/MS through Q-Exactive Orbitrap mass spectrometer. Resolving power of Q-Exactive Orbitrap mass spectrometer for MS/MS was set at 17,500, the Collision energy was set at 10, 20, and 40 eV. The mass spectrometer operated as follows: spray voltage, 3500 V; sheath gas flow rate, 40 arbitrary units for positive ion mode and 35 arbitrary units for negative ion mode; auxiliary gas flow rate, 10 arbitrary units for positive ion mode and 8 arbitrary units for negative ion mode; capillary temperature, 320 °C. For on-tissue MALDI-MS/MS analysis, CID was turn on, solation window was set to ± 0.6% of precursor, fragments shot was set to 500, and the laser application mode was ISD. Then, the analytes were further identified based on the similarity of MS/MS spectra of potential metabolites in cancer tissues and MS/MS spectra of metabolites in LuMet, HMDB, METLIN, and LIPID MAPS database. The fragment ions of putative metabolites should be reasonably attributed to the structure of annotated metabolites. The structure-specific pattern ions of the target analyte were demonstrated in Supplementary Figs. 47–74.

### Differential metabolic pathway screening

#### Spatially resolved transcriptomics-driven differential metabolic pathway screening

Marker genes in different tissue regions of gastric cancer tissue were extracted by Loupe Browser 6 software. Differential expressed genes with fold change ≥ 2 and false discovery rate < 0.05 between the tumor and peritumoral tissue regions were selected. To integrate with the metabolism data, the KEGG pathway enrichment analyses of DEGs were performed by KOBAS-i^82^.

#### Spatially resolved metabolomics and lipidomics-driven differential metabolic pathway screening

Discriminating metabolites and lipids between the tumor and peritumoral tissue regions were screened by spatially resolved metabolomics and lipidomics analysis. Then, the discriminating metabolites and lipids were imported into KEGG and MetaboAnalyst 5.0 to perform metabolic pathway analysis, and the metabolic pathways with the most significant changes in metabolites and lipids can be screened.

#### Metabolome-transcriptome association network

Marker genes in different tissue regions with fold change ≥ 2 and *P*-value < 0.05 between the tumor and peritumoral tissue regions were selected, and marker metabolites and lipids in different tissues regions with fold change ≥ 1.5 were selected for metabolome-transcriptome association network building. Sankey diagram for the metabolome-transcriptome association network help to visualize the spatial relationship between metabolites and gene expression in tumor or surrounding normal tissues.

### Cell annotation

The Cell Ranger available from 10× Genomics with default parameters, was used to conduct the cell clustering analysis based on the UMAP result. A total of 10 clusters were identified and candidate maker genes of each cluster were selected as differentially overexpressed genes compared to other clusters. We annotated cell clusters based on the expression of curated known cell markers. The above cell markers with cell types were selected from Cell Markers and CellTypist database^83,84^ and these selected marker genes in the annotated clusters have log_2_ fold change > 1 and *P*-value < 0.01 to other clusters.

### Immunohistochemistry

The expression of CD20, CD38, and AOC1 in adjacent cancer tissue sections were explored. After fixed in paraformaldehyde for 15 min, the sections were immersed in 0.25% Triton X-100 (ZLI-9308, Zhongshan Goldenbridge Biotechnology Ltd. Co., Beijing, China) for 15 min and blocked with 1% bovine serum albumin (Sigma-Aldrich, St. Louis, MO) for 30 min. Then, the sections were incubated with antibodies against CD20 (Abcam; ab78237; 1:100), CD38 (Abcam; ab108403; 1:500), and AOC1 (ABP1, Abcam; ab278497; 1:500) at 4 °C overnight, followed by rewarming at room temperature for 30 min. Then, PV-9000 two-step immunohistochemical kit was used according to the manufacturer’s instructions (PV-9000, Zhongshan Goldenbridge Biotechnology Ltd. Co., Beijing, China), and a DAB kit was used subsequently to detect antigen-antibody binding.

### Reporting summary

Further information on research design is available in the Nature Portfolio Reporting Summary linked to this article.

## Supplementary information

Supplementary Information

Description of Additional Supplementary Files

Supplementary Data 1

Supplementary Data 2

Supplementary Data 3

Supplementary Data 4

Supplementary Data 5

Supplementary Data 6

Reporting Summary

## Data Availability

Sequencing and spatial transcriptomics data generated in this study have been deposited in the GSA (Genome Sequence Archive)-human database of the National Genomics Data Center under accession number HRA003070. The raw ADADESI-MS data and MALDI-MS data generated in this study have been deposited in the OMIX database of the National Genomics Data Center under accession number OMIX002397. ADADESI-MS imaging and MALDI-MS imaging data have been deposited in the Metaspace database which allows visualization of mass spectrometry imaging results, and all the MS imaging data can be directly download from [https://metaspace2020.eu/datasets?q = 710b1782-343a-11ed-89bf-738830e26a67]. The remaining data are available within the Article and Supplementary Information.

## References

[1] Pavlova NN, Thompson CB (2016). The emerging hallmarks of cancer metabolism. Cell Metab..

[2] Reinfeld BI (2021). Cell-programmed nutrient partitioning in the tumour microenvironment. Nature.

[3] Chang CH (2015). Metabolic competition in the tumor microenvironment is a driver of cancer progression. Cell.

[4] Bayik D, Lathia JD (2021). Cancer stem cell-immune cell crosstalk in tumour progression. Nat. Rev. Cancer.

[5] Karczewski KJ, Snyder MP (2018). Integrative omics for health and disease. Nat. Rev. Genet..

[6] Wu C, Dill AL, Eberlin LS, Cooks RG, Ifa DR (2013). Mass spectrometry imaging under ambient conditions. Mass Spectrom. Rev..

[7] Van de Plas R, Yang J, Spraggins J, Caprioli RM (2015). Image fusion of mass spectrometry and microscopy: A multimodality paradigm for molecular tissue mapping. Nat. Methods.

[8] Wang L (2022). Spatially resolved isotope tracing reveals tissue metabolic activity. Nat. Methods.

[9] Sun C (2019). Spatially resolved metabolomics to discover tumor-associated metabolic alterations. Proc. Natl Acad. Sci. USA..

[10] Lewis SM (2021). Spatial omics and multiplexed imaging to explore cancer biology. Nat. Methods.

[11] Ganesh S (2021). Spatially resolved 3D metabolomic profiling in tissues. Sci. Adv..

[12] Guenther S (2015). Spatially resolved metabolic phenotyping of breast cancer by desorption electrospray ionization mass spectrometry. Cancer Res..

[13] Eberlin LS (2012). Classifying human brain tumors by lipid imaging with mass spectrometry. Cancer Res..

[14] Gouw AM (2019). The MYC oncogene cooperates with sterol-regulated element-binding protein to regulate lipogenesis essential for neoplastic growth. Cell Metab..

[15] Ståhl PL (2016). Visualization and analysis of gene expression in tissue sections by spatial transcriptomics. Science.

[16] Saltz J (2018). Spatial organization and molecular correlation of tumor-infiltrating lymphocytes using deep learning on pathology images. Cell Rep..

[17] Andersson A (2021). Spatial deconvolution of HER2-positive breast cancer delineates tumor-associated cell type interactions. Nat. Commun..

[18] Ravi VM (2022). Spatially resolved multi-omics deciphers bidirectional tumor-host interdependence in glioblastoma. Cancer Cell.

[19] Hunter MV, Moncada R, Weiss JM, Yanai I, White RM (2021). Spatially resolved transcriptomics reveals the architecture of the tumor-microenvironment interface. Nat. Commun..

[20] Moncada R (2020). Integrating microarray-based spatial transcriptomics and single-cell RNA-seq reveals tissue architecture in pancreatic ductal adenocarcinomas. Nat. Biotechnol..

[21] Sung H (2021). Global Cancer Statistics 2020: GLOBOCAN estimates of incidence and mortality worldwide for 36 cancers in 185 countries. CA Cancer J. Clin..

[22] Lee I-S (2021). Transcriptomic profiling identifies a risk stratification signature for predicting peritoneal recurrence and micrometastasis in gastric cancer. Clin. Cancer Res..

[23] Kadam W, Wei B, Li F (2021). Metabolomics of gastric cancer. Adv. Exp. Med. Biol..

[24] Liu Z (2021). Genomic and transcriptomic profiling of hepatoid adenocarcinoma of the stomach. Oncogene.

[25] Kumar V (2022). Single-Cell Atlas of lineage states, tumor microenvironment, and subtype-specific expression programs in gastric cancer. Cancer Disco..

[26] Zhang M (2021). Dissecting transcriptional heterogeneity in primary gastric adenocarcinoma by single cell RNA sequencing. Gut.

[27] Sathe A (2020). Single-cell genomic characterization reveals the cellular reprogramming of the gastric tumor microenvironment. Clin. Cancer Res..

[28] Wang R (2021). Single-cell dissection of intratumoral heterogeneity and lineage diversity in metastatic gastric adenocarcinoma. Nat. Med..

[29] Geiger R (2016). L-Arginine Modulates T cell metabolism and enhances survival and anti-tumor activity. Cell.

[30] Poillet-Perez L (2018). Autophagy maintains tumour growth through circulating arginine. Nature.

[31] Liu M (2020). Inhibiting both proline biosynthesis and lipogenesis synergistically suppresses tumor growth. J. Exp. Med..

[32] Snaebjornsson MT, Janaki-Raman S, Schulze A (2020). Greasing the wheels of the cancer machine: The role of lipid metabolism in cancer. Cell Metab..

[33] Röhrig F, Schulze A (2016). The multifaceted roles of fatty acid synthesis in cancer. Nat. Rev. Cancer.

[34] Broadfield LA, Pane AA, Talebi A, Swinnen JV, Fendt SM (2021). Lipid metabolism in cancer: New perspectives and emerging mechanisms. Dev. Cell.

[35] Cao HL (2016). Clinical features of upper gastrointestinal serrated lesions: An endoscopy database analysis of 98746 patients. World J. Gastroenterol..

[36] Dong YW (2014). Sulfatide epigenetically regulates miR-223 and promotes the migration of human hepatocellular carcinoma cells. J. Hepatol..

[37] Chapman NM, Chi H (2022). Metabolic adaptation of lymphocytes in immunity and disease. Immunity.

[38] Bidault G (2021). SREBP1-induced fatty acid synthesis depletes macrophages antioxidant defences to promote their alternative activation. Nat. Metab..

[39] Smith B (2016). Addiction to coupling of the warburg effect with glutamine catabolism in cancer cells. Cell Rep..

[40] Gaber T, Strehl C, Buttgereit F (2017). Metabolic regulation of inflammation. Nat. Rev. Rheumatol..

[41] Manzo T (2020). Accumulation of long-chain fatty acids in the tumor microenvironment drives dysfunction in intrapancreatic CD8+ T cells. J. Exp. Med..

[42] Xu S (2021). Uptake of oxidized lipids by the scavenger receptor CD36 promotes lipid peroxidation and dysfunction in CD8(+) T cells in tumors. Immunity.

[43] Buckley CD, Gilroy DW, Serhan CN (2014). Proresolving lipid mediators and mechanisms in the resolution of acute inflammation. Immunity.

[44] Luengo A, Gui DY, Vander Heiden MG (2017). Targeting Metabolism for Cancer Therapy. Cell Chem. Biol..

[45] Martinez-Outschoorn UE, Peiris-Pages M, Pestell RG, Sotgia F, Lisanti MP (2017). Cancer metabolism: A therapeutic perspective. Nat. Rev. Clin. Oncol..

[46] Song M (2021). Cancer-associated fibroblast-mediated cellular crosstalk supports hepatocellular carcinoma progression. Hepatology.

[47] Leone RD, Powell JD (2020). Metabolism of immune cells in cancer. Nat. Rev. Cancer.

[48] Chen Y, McAndrews KM, Kalluri R (2021). Clinical and therapeutic relevance of cancer-associated fibroblasts. Nat. Rev. Clin. Oncol..

[49] Eberlin LS (2014). Alteration of the lipid profile in lymphomas induced by MYC overexpression. Proc. Natl. Acad. Sci. USA..

[50] Pirro V (2017). Intraoperative assessment of tumor margins during glioma resection by desorption electrospray ionization-mass spectrometry. Proc. Natl. Acad. Sci. USA..

[51] Wishart DS, Mandal R, Stanislaus A, Ramirez-Gaona M (2016). Cancer metabolomics and the human metabolome database. Metabolites.

[52] He J (2018). A sensitive and wide coverage ambient mass spectrometry imaging method for functional metabolites based molecular histology. Adv. Sci..

[53] Norris JL, Caprioli RM (2013). Analysis of tissue specimens by matrix-assisted laser desorption/ionization imaging mass spectrometry in biological and clinical research. Chem. Rev..

[54] Sun C, Wang F, Zhang Y, Yu J, Wang X (2020). Mass spectrometry imaging-based metabolomics to visualize the spatially resolved reprogramming of carnitine metabolism in breast cancer. Theranostics.

[55] Chen CL (2021). Arginine is an epigenetic regulator targeting TEAD4 to modulate OXPHOS in prostate cancer cells. Nat. Commun..

[56] Chantranupong L (2016). The CASTOR Proteins Are Arginine Sensors for the mTORC1 Pathway. Cell.

[57] Changou CA (2014). Arginine starvation-associated atypical cellular death involves mitochondrial dysfunction, nuclear DNA leakage, and chromatin autophagy. Proc. Natl. Acad. Sci. USA..

[58] Kremer JC (2017). Arginine deprivation inhibits the Warburg effect and upregulates glutamine anaplerosis and serine biosynthesis in ASS1-deficient cancers. Cell Rep..

[59] Madeo F, Eisenberg T, Pietrocola F, Kroemer G (2018). Spermidine in health and disease. Science.

[60] McNamara KM, Gobert AP, Wilson KT (2021). The role of polyamines in gastric cancer. Oncogene.

[61] Bachmann AS, Geerts D (2018). Polyamine synthesis as a target of MYC oncogenes. J. Biol. Chem..

[62] Altman BJ, Stine ZE, Dang CV (2016). From Krebs to clinic: Glutamine metabolism to cancer therapy. Nat. Rev. Cancer.

[63] Leone RD (2019). Glutamine blockade induces divergent metabolic programs to overcome tumor immune evasion. Science.

[64] Byun JK (2020). Inhibition of Glutamine utilization synergizes with immune checkpoint inhibitor to promote antitumor immunity. Mol. Cell.

[65] Jha AK (2015). Network integration of parallel metabolic and transcriptional data reveals metabolic modules that regulate macrophage polarization. Immunity.

[66] Massari NA, Nicoud MB, Medina VA (2020). Histamine receptors and cancer pharmacology: An update. Br. J. Pharmacol..

[67] Li H (2022). The allergy mediator histamine confers resistance to immunotherapy in cancer patients via activation of the macrophage histamine receptor H1. Cancer Cell.

[68] Sarasola MP, Táquez Delgado MA, Nicoud MB, Medina VA (2021). Histamine in cancer immunology and immunotherapy. Current status and new perspectives. Pharm. Res. Perspect..

[69] Yang XD (2011). Histamine deficiency promotes inflammation-associated carcinogenesis through reduced myeloid maturation and accumulation of CD11b+Ly6G+ immature myeloid cells. Nat. Med..

[70] Ruffell B, Coussens LM (2011). Histamine restricts cancer: Nothing to sneeze at. Nat. Med..

[71] Bian X (2021). Lipid metabolism and cancer. J. Exp. Med..

[72] DeBerardinis RJ, Chandel NS (2016). Fundamentals of cancer metabolism. Sci. Adv..

[73] Horton JD (2003). Combined analysis of oligonucleotide microarray data from transgenic and knockout mice identifies direct SREBP target genes. Proc. Natl Acad. Sci. USA..

[74] Triki M (2020). mTOR signaling and SREBP activity increase FADS2 expression and can activate sapienate biosynthesis. Cell Rep..

[75] Köberlin MS (2015). A conserved circular network of coregulated lipids modulates innate immune responses. Cell.

[76] Lim SA (2021). Lipid signalling enforces functional specialization of T(reg) cells in tumours. Nature.

[77] Liu X (2021). Reprogramming lipid metabolism prevents effector T cell senescence and enhances tumor immunotherapy. Sci. Transl. Med..

[78] Ma X (2018). Cholesterol negatively regulates IL-9-producing CD8(+) T cell differentiation and antitumor activity. J. Exp. Med..

[79] Ma X (2019). Cholesterol Induces CD8+ T Cell Exhaustion in the Tumor Microenvironment. Cell Metab..

[80] Picarda E, Ren X, Zang X (2019). Tumor Cholesterol up, T cells down. Cell Metab..

[81] He J (2018). MassImager: A software for interactive and in-depth analysis of mass spectrometry imaging data. Anal. Chim. Acta..

[82] Bu D (2021). KOBAS-i: Intelligent prioritization and exploratory visualization of biological functions for gene enrichment analysis. Nucleic Acids Res..

[83] Zhang X (2019). CellMarker: A manually curated resource of cell markers in human and mouse. Nucleic Acids Res..

[84] Domínguez Conde, C. et al. Cross-tissue immune cell analysis reveals tissue-specific features in humans. *Science* **376**, eabl5197 (2022). 10.1126/science.abl5197 PMC7612735 35549406

