# Supplementary material for: Spatially resolved multi-omics highlights cell-specific metabolic remodeling and interactions in gastric cancer
Source: Nat Commun. 2023 May 10;14:2692. doi: 10.1038/s41467-023-38360-5 (PMC10172194; doi:10.1038/s41467-023-38360-5)
Supplement: Supplementary file 1 — Supplementary Information [file 41467_2023_38360_MOESM1_ESM.pdf]

Supplementary information

Spatially resolved multi-omics highlights cell-specific metabolic remodeling and interactions in gastric cancer

Chenglong Sun[1,2,3,†], Anqiang Wang[4,†], Yanhe Zhou[1,†], Panpan Chen[2,3], Xiangyi Wang[1], Jianpeng Huang[1], Jiamin Gao[1], Xiao Wang[2,3], Liebo Shu[5], Jiawei Lu[5], Wentao Dai[6,7*], Zhaode Bu[4*], Jiafu Ji[4*], Jiuming He[1,8*]

[1] State Key Laboratory of Bioactive Substance and Function of Natural Medicines, Institute of Materia Medica, Chinese Academy of Medical Sciences and Peking Union Medical College, Beijing 100050, China.
[2] Key Laboratory for Applied Technology of Sophisticated Analytical Instruments of Shandong Province, Shandong Analysis and Test Center, Qilu University of Technology (Shandong Academy of Sciences), Jinan 250014, China.
[3] Key Laboratory for Natural Active Pharmaceutical Constituents Research in Universities of Shandong Province, School of Pharmaceutical Sciences, Qilu University of Technology (Shandong Academy of Sciences), Jinan, 250014, China.
[4] Gastrointestinal Cancer Center, Key Laboratory of Carcinogenesis and Translational Research (Ministry of Education), Peking University Cancer Hospital and Institute, Beijing 100142, China.
[5] Shanghai Luming Biological Technology co.Ltd, Shanghai 201102, China.
[6] NHC Key Lab of Reproduction Regulation (Shanghai Institute for Biomedical and Pharmaceutical Technologies) & Shanghai Engineering Research Center of Pharmaceutical Translation, Fudan University, Shanghai 200080, China.
[7] Shanghai Key Laboratory of Gastric Neoplasms, Department of General Surgery, Shanghai Institute of Digestive Surgery, Ruijin Hospital, Shanghai Jiao Tong University School of Medicine, Shanghai 200025, China.
[8] NMPA Key Laboratory of safety research and evaluation of Innovative Drug, Institute of Materia Medica, Chinese Academy of Medical Sciences and Peking Union Medical College, Beijing 100050, China.
† These authors contributed equally: Chenglong Sun, Anqiang Wang, Yanhe Zhou.
* These authors jointly supervised this work: Wentao Dai, dwent_job@163.com; Zhaode Bu, buzhaode@cjcrcn.org; Jiafu Ji, jijiafu@hsc.pku.edu.cn; Jiuming He, hejiuming@imm.ac.cn

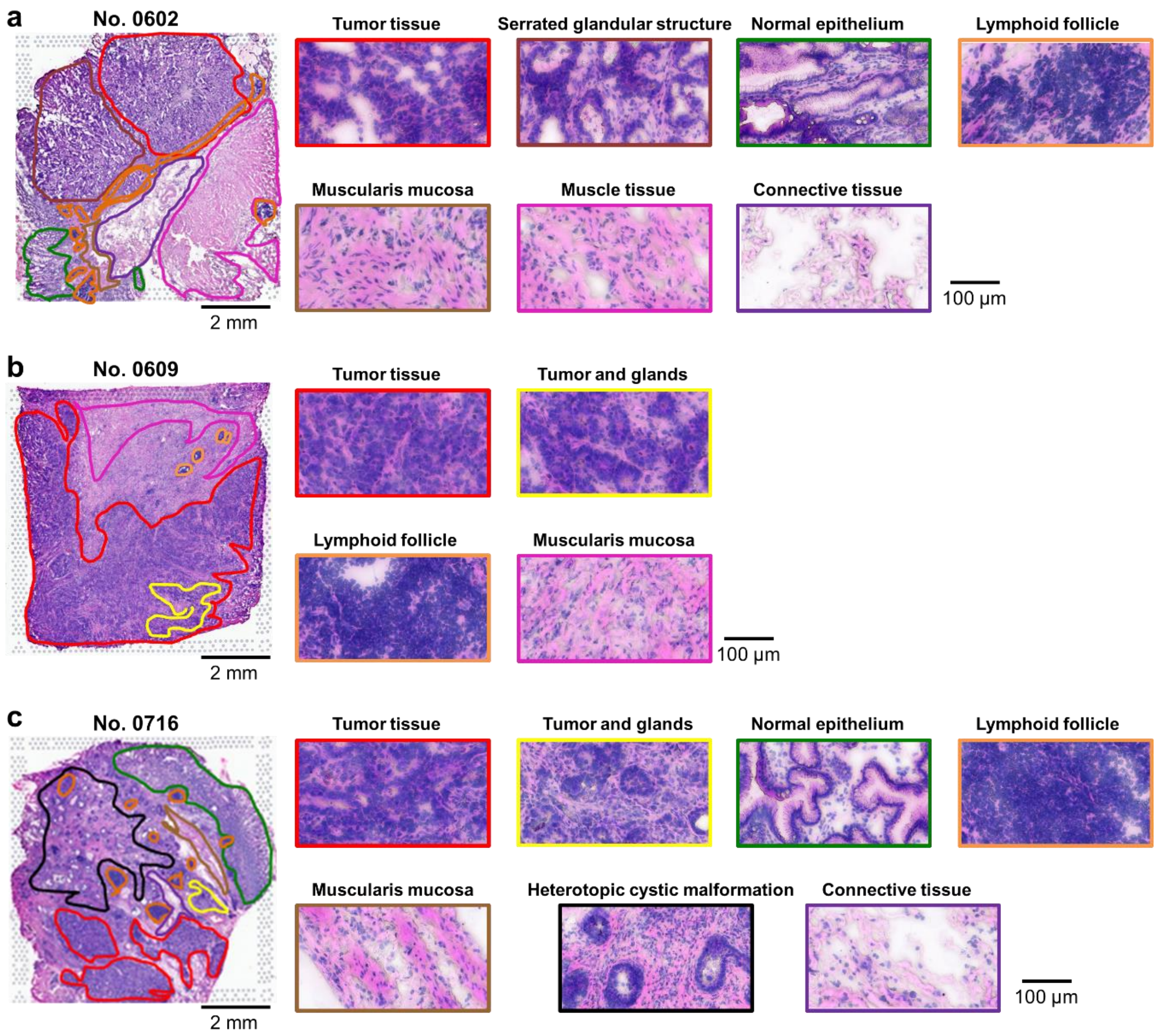

Supplementary Figure 1 | H&E stain image of gastric cancer tissue section from different patients. a, Typical H&E staining image of gastric cancer tissue for patients “No.0602”. **b,** Typical H&E staining image of gastric cancer tissue for patients “No.0609”. **c,** Typical H&E staining image of gastric cancer tissue for patients “No.0716”. scale bar = 2 mm for whole tissue sections, scale bar = 100 μm for magnified image. The experiment was repeated three times.

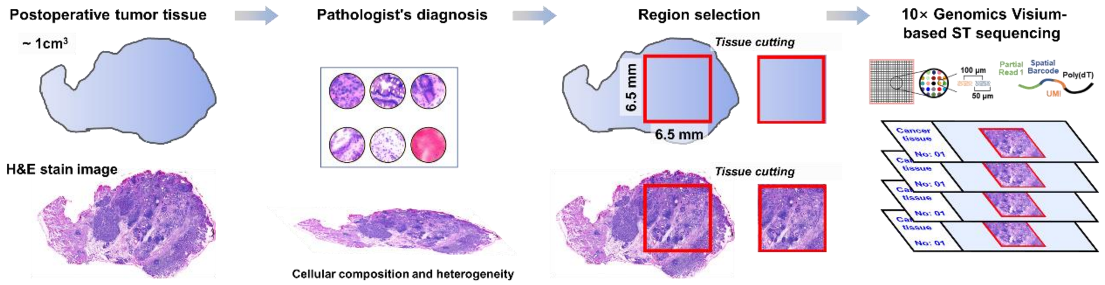

Supplementary Figure 2 | The process of selecting scan area for 10× Genomics sequencing.

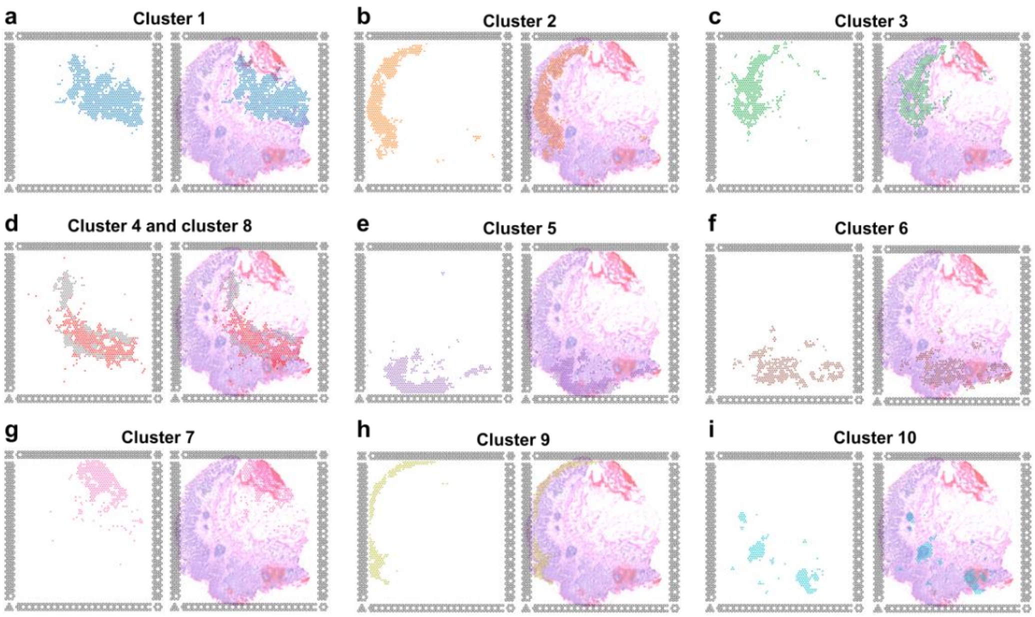

Supplementary Figure 3 | Visium array spots colored by graph-based clustering algorithm for gastric cancer tissue section. a, Graph-based Visium array spots of cluster 1. **b,** Graph-based Visium array spots of cluster 2. **c,** Graph-based Visium array spots of cluster 3. **d,** Graph-based Visium array spots of cluster 4 and cluster 8. **e,** Graph-based Visium array spots of cluster 5. **f,** Graph-based Visium array spots of cluster 6. **g,** Graph-based Visium array spots of cluster 7. **h,** Graph-based Visium array spots of cluster 9. **i,** Graph-based Visium array spots of cluster 10.

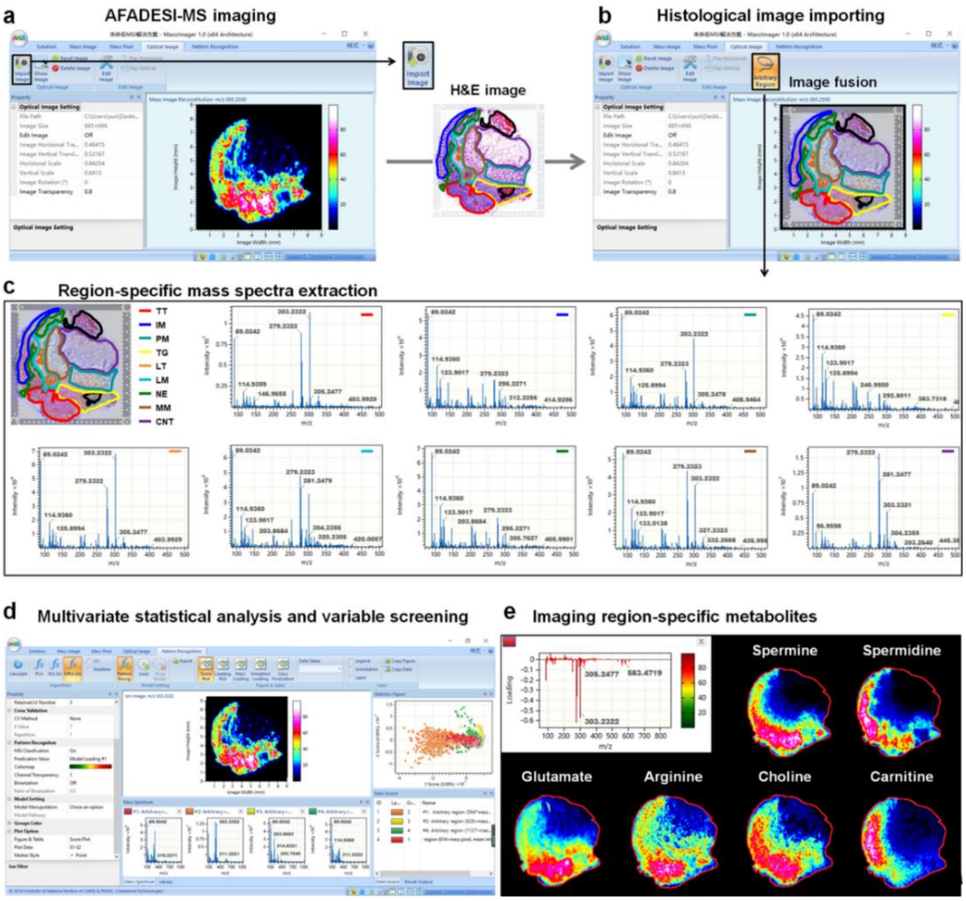

Supplementary Figure 4 | Spots-labeled H&E image was imported into AFADESI-MSI software MassImager for image fusion and feature metabolites screening. a, The operation interface of MassImager software. **b,** Import histological staining images into MassImager software. **c,** Extraction of AFADESI MS data from different tissue regions. **d,** *In situ* multivariate statistical analysis. **e,** MS images of screened region-specific metabolites.

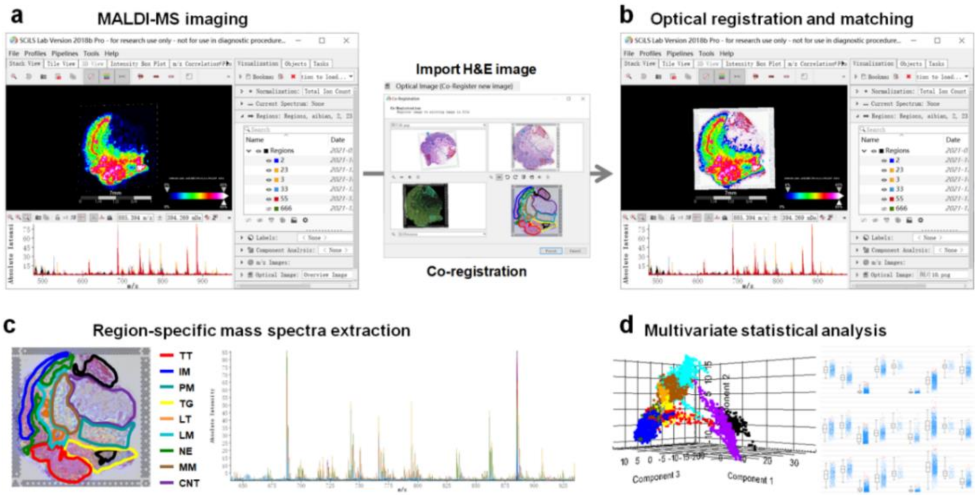

Supplementary Figure 5 | Spots-labeled H&E image was imported into MALDI-MSI software SCiLS Lab for image fusion and feature lipids screening. a, The operation interface of SCiLS Lab software. **b,** Import histological staining images into SCiLS Lab software. **c,** Extraction of MALDI MS data from different tissue regions. **d,** *In situ* multivariate statistical analysis.

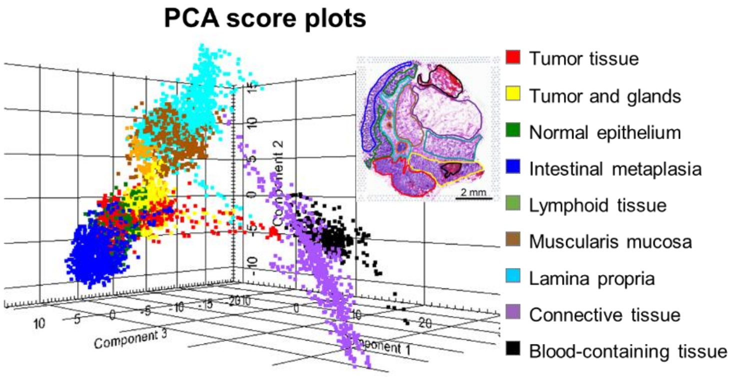

Supplementary Figure 6 | PCA score plots for different gastric cancer tissue regions based on the metabolite and lipid profiles.

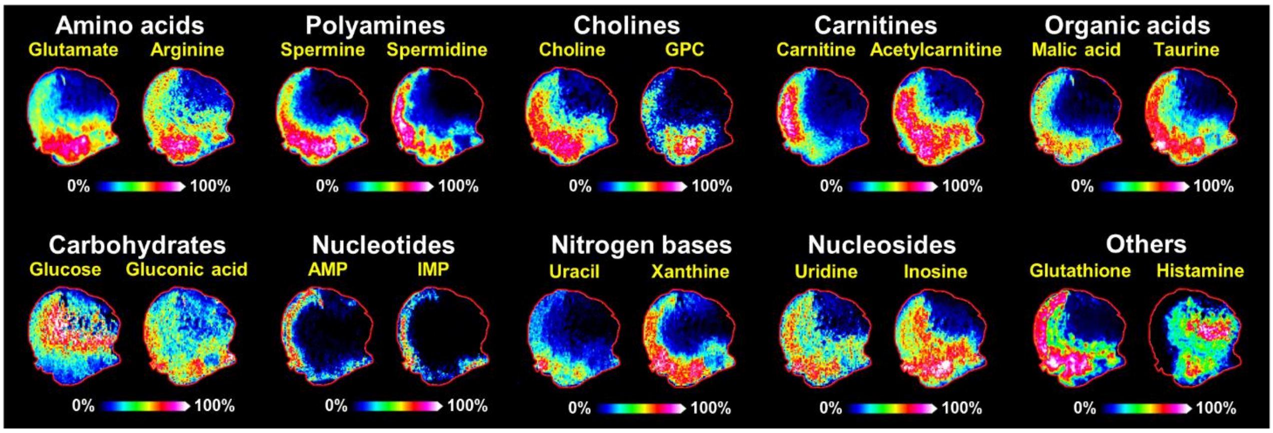

Supplementary Figure 7 | Representative metabolites that imaged by AFADESI-MSI based platform. GCP: glycerophosphorylcholine, AMP: adenosine monophosphate, IMP: inosine monophosphate, intensity in colour scale is relative value.

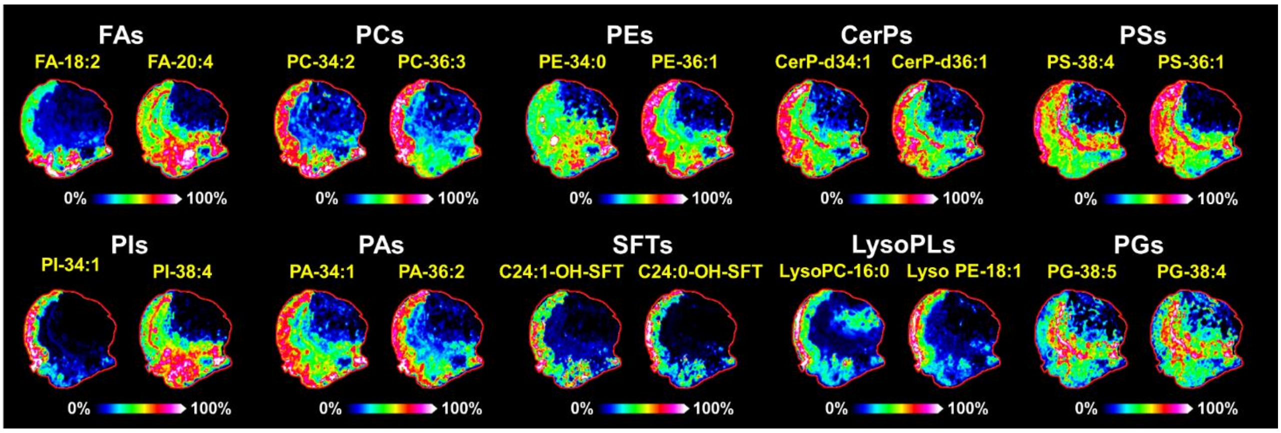

Supplementary Figure 8 | Representative lipids that imaged by MALDI-MSI based platform. PC: Phosphatidylcholine, LysoPL: Lysophosphatide, PC: phosphatidylcholine, PE: phosphatidylethanolamine, CerP: ceramide-phosphate, PS: phosphatidylserine, PG: phosphatidylglycerol, PI: phosphatidylinositol, PA: phosphatidic acid, SFT: sulfatide, intensity in colour scale is relative value.

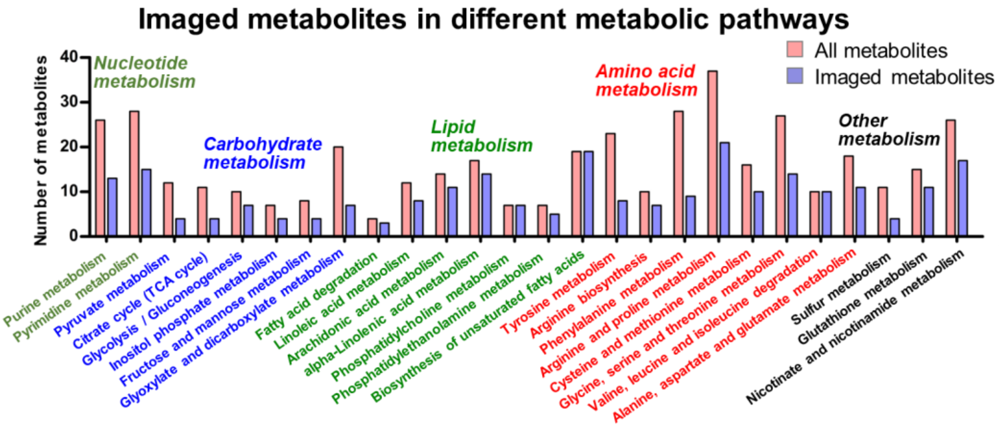

Supplementary Figure 9 | Imaged metabolites and lipids in different metabolic pathways.

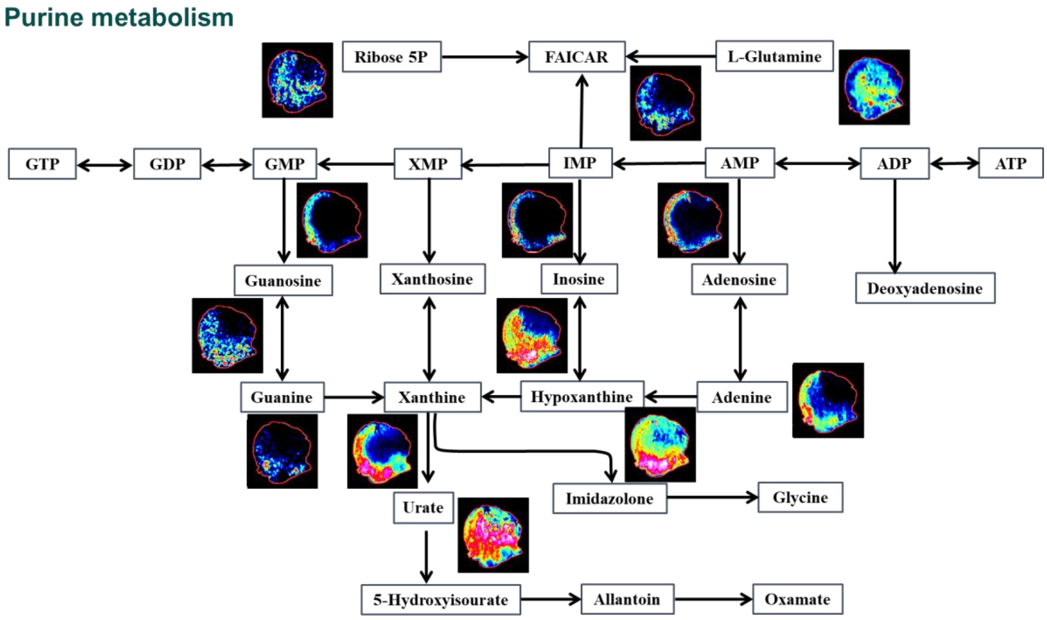

Supplementary Figure 10 | MS images of detected metabolites in purine metabolism pathway (intensity in colour scale is relative value).

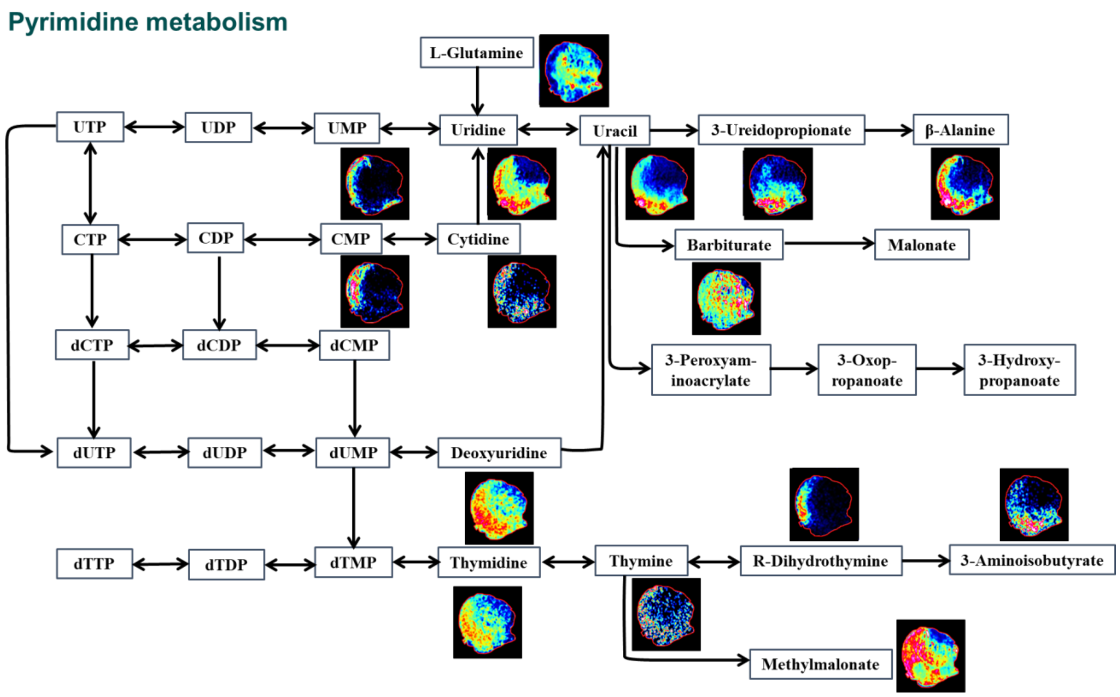

Supplementary Figure 11 | MS images of detected metabolites in pyrimidine metabolism pathway (intensity in colour scale is relative value).

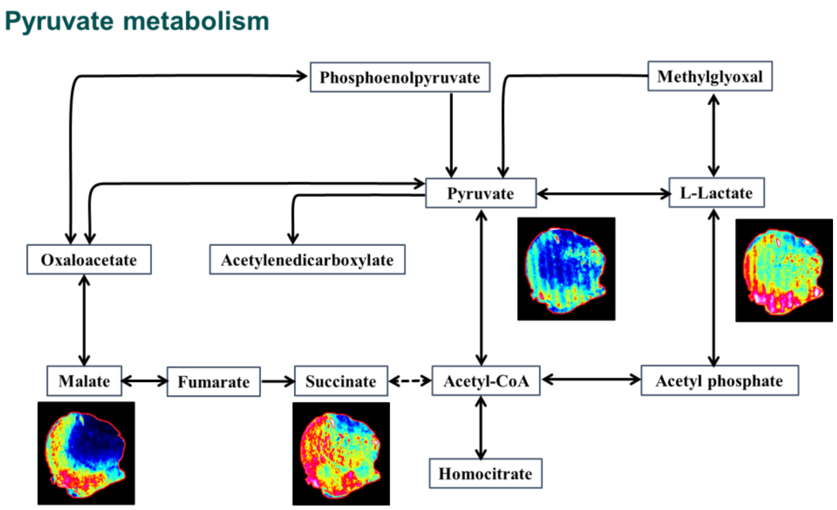

Supplementary Figure 12 | MS images of detected metabolites in pyruvate metabolism pathway (intensity in colour scale is relative value).

Citrate cycle (TCA cycle)

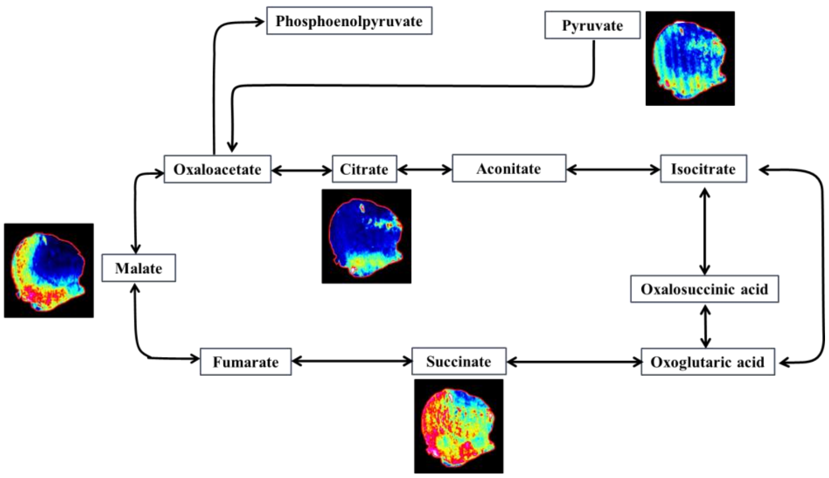

Supplementary Figure 13 | MS images of detected metabolites in citrate cycle pathway (intensity in colour scale is relative value).

Glycolysis / Gluconeogenesis

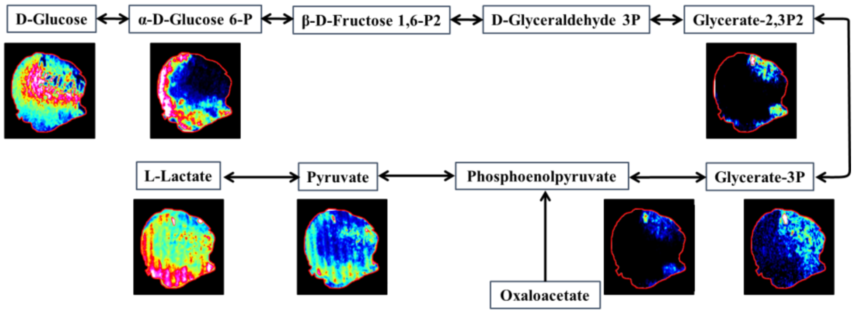

Supplementary Figure 14 | MS images of detected metabolites in glycolysis and gluconeogenesis pathways (intensity in colour scale is relative value).

Inositol phosphate metabolism

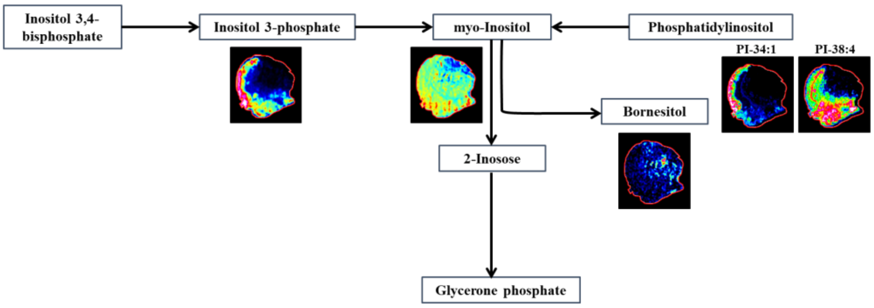

Supplementary Figure 15 | MS images of detected metabolites in inositol phosphate pathway (intensity in colour

scale is relative value).

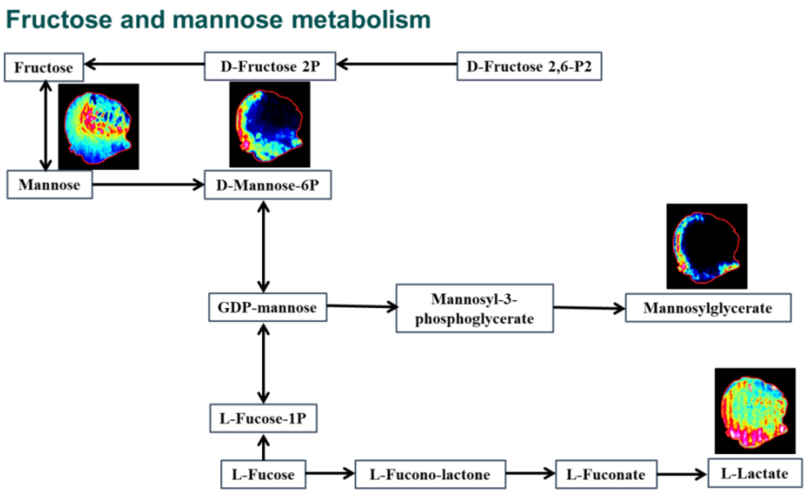

Supplementary Figure 16 | MS images of detected metabolites in fructose and mannose metabolism pathway (intensity in colour scale is relative value).

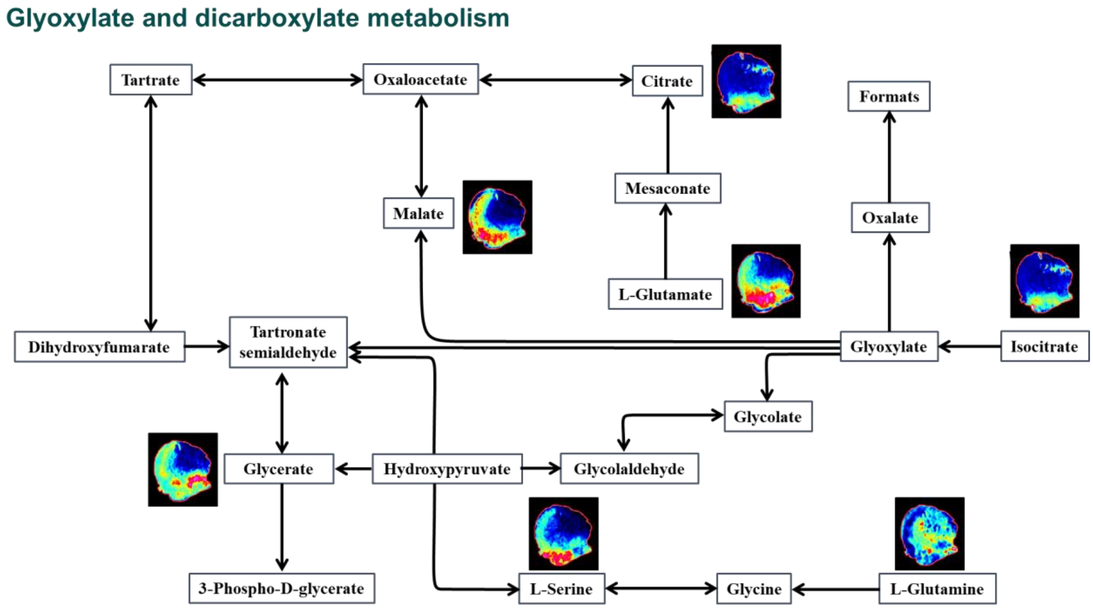

Supplementary Figure 17 | MS images of detected metabolites in glyoxylate and dicarboxylate pathway (intensity in colour scale is relative value).

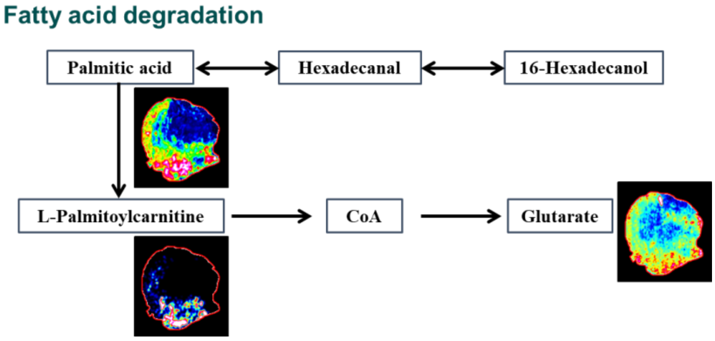

Supplementary Figure 18 | MS images of detected metabolites in fatty acid degradation pathway (intensity in colour scale is relative value).

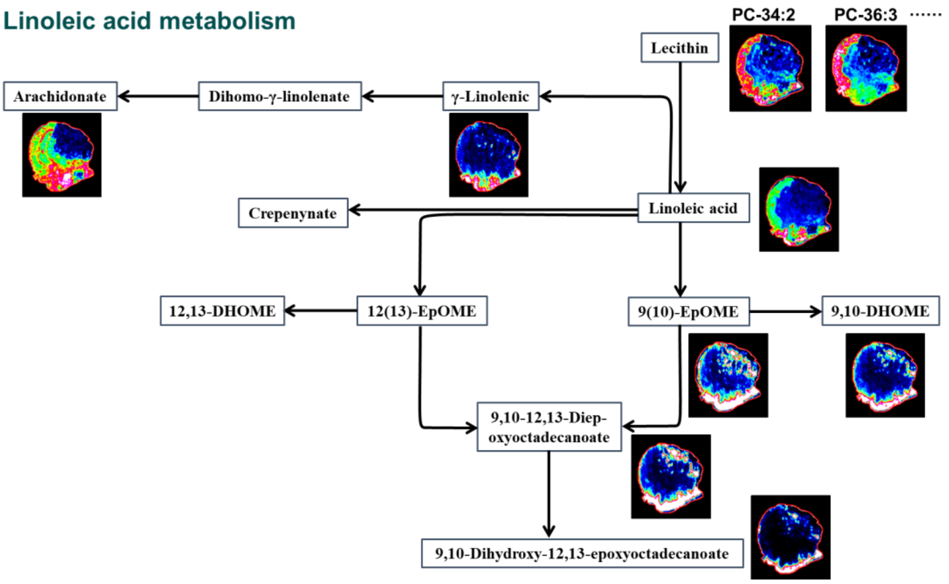

Supplementary Figure 19 | MS images of detected metabolites in linoleic acid metabolism pathway (intensity in colour scale is relative value).

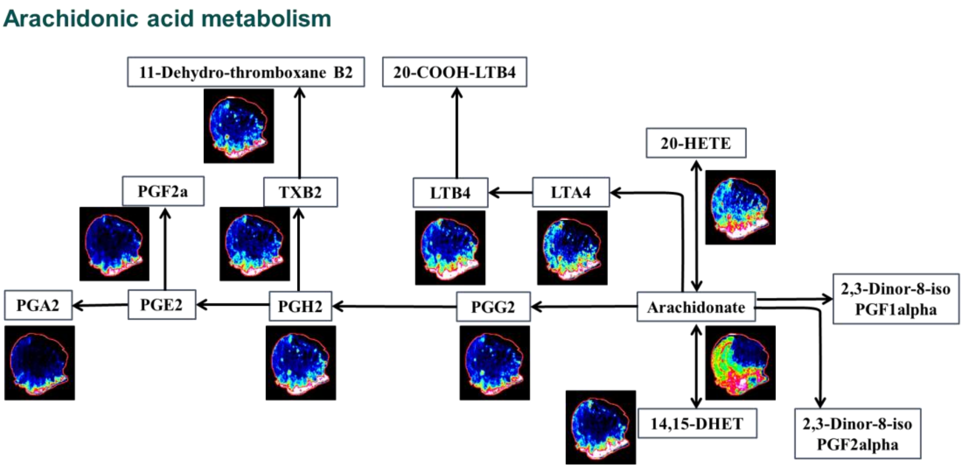

Supplementary Figure 20 | MS images of detected metabolites in arachidonic acid metabolism pathway (intensity in colour scale is relative value).

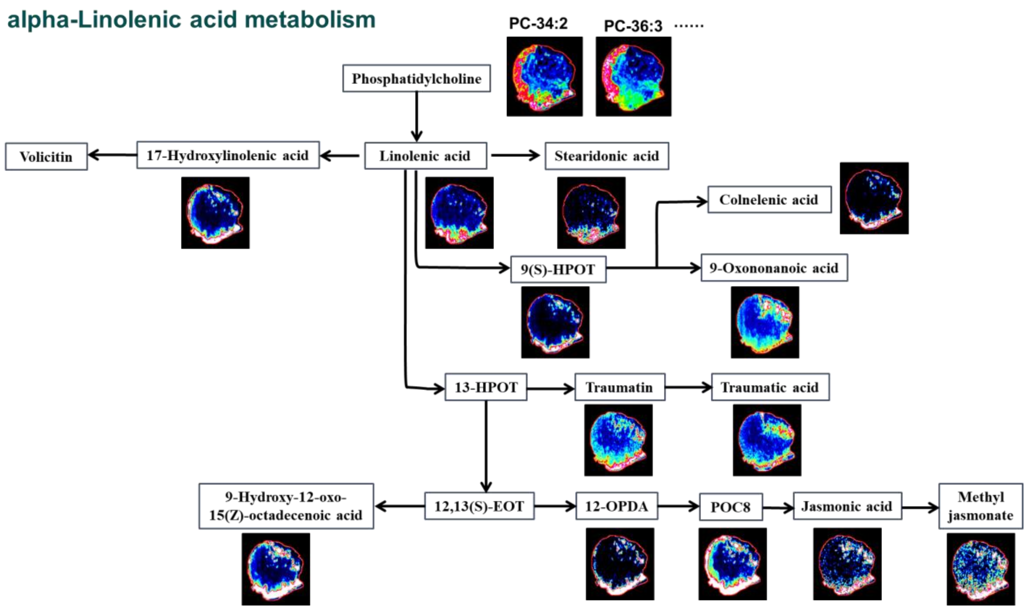

Supplementary Figure 21 | MS images of detected metabolites in alpha-linolenic acid metabolism pathway (intensity in colour scale is relative value).

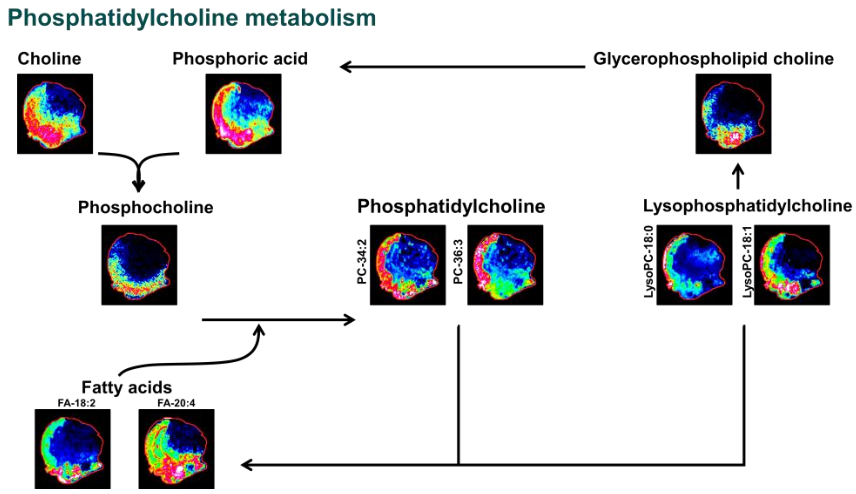

Supplementary Figure 22 | MS images of detected metabolites in phosphatidylcholine metabolism pathway (intensity in colour scale is relative value).

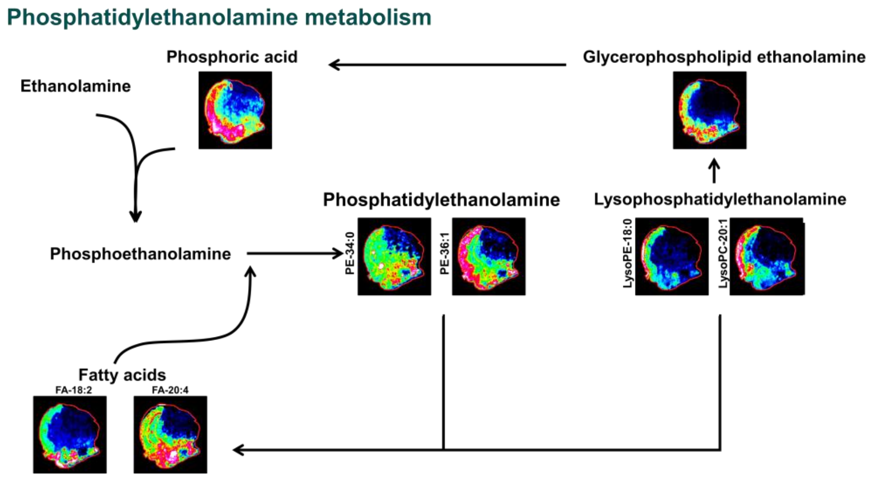

Supplementary Figure 23 | MS images of detected metabolites in phosphatidylethanolamine metabolism pathway (intensity in colour scale is relative value).

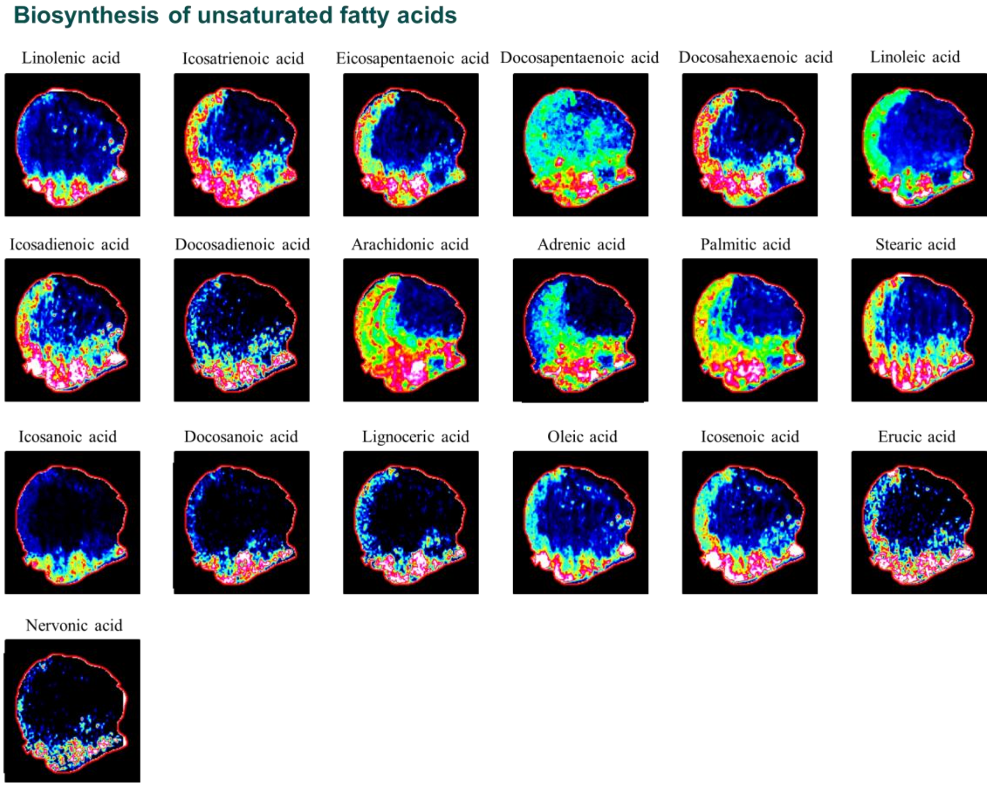

Supplementary Figure 24 | MS images of detected metabolites in biosynthesis of unsaturated fatty acids pathway (intensity in colour scale is relative value).

Tyrosine metabolism

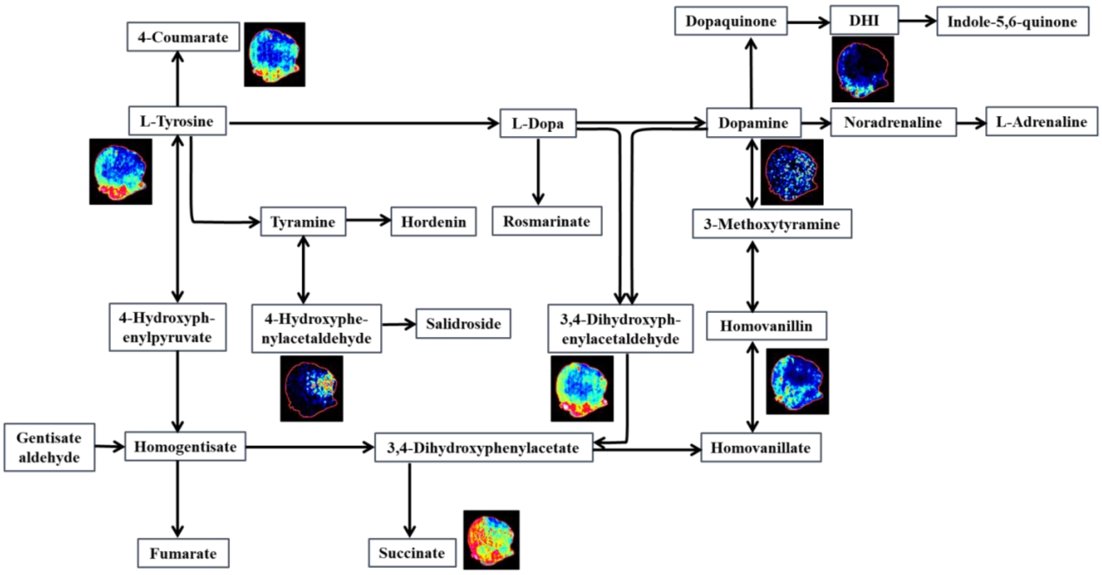

Supplementary Figure 25 | MS images of detected metabolites in tyrosine metabolism pathway (intensity in colour scale is relative value).

Arginine biosynthesis

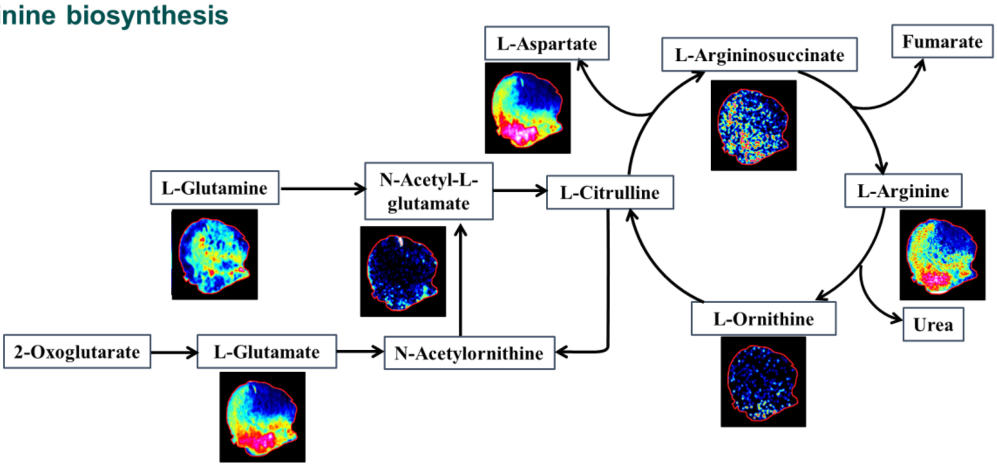

Supplementary Figure 26 | MS images of detected metabolites in arginine biosynthesis pathway (intensity in colour scale is relative value).

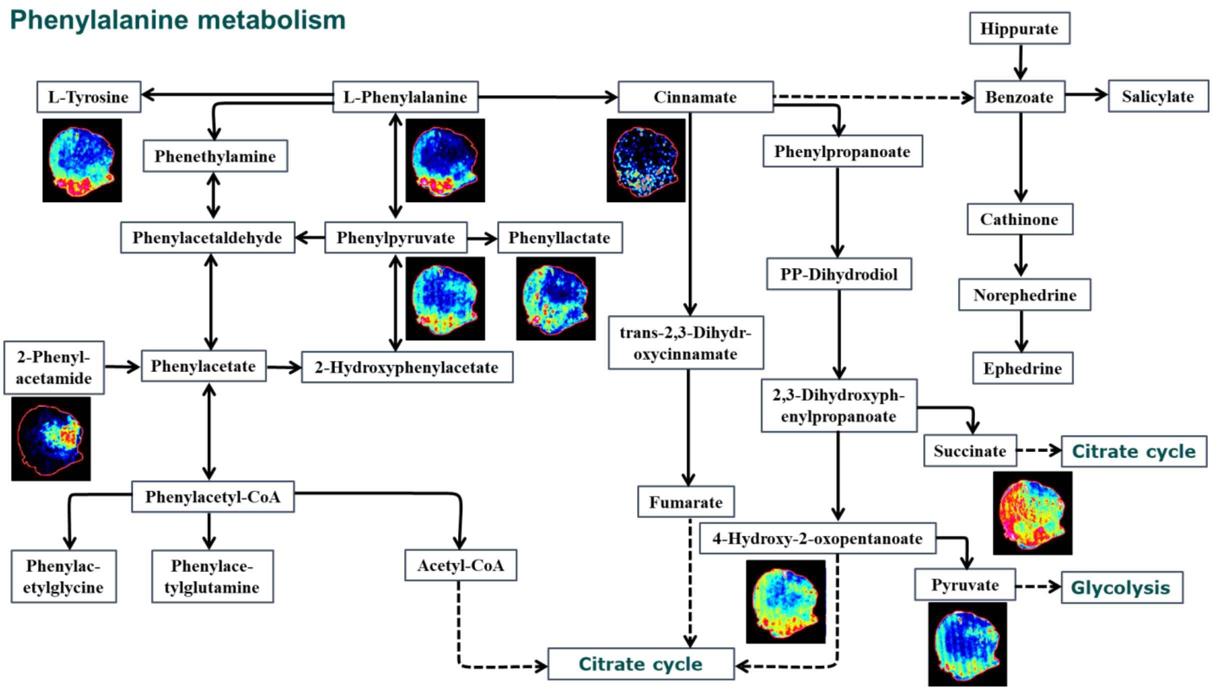

Supplementary Figure 27 | MS images of detected metabolites in phenylalanine metabolism pathway (intensity in colour scale is relative value).

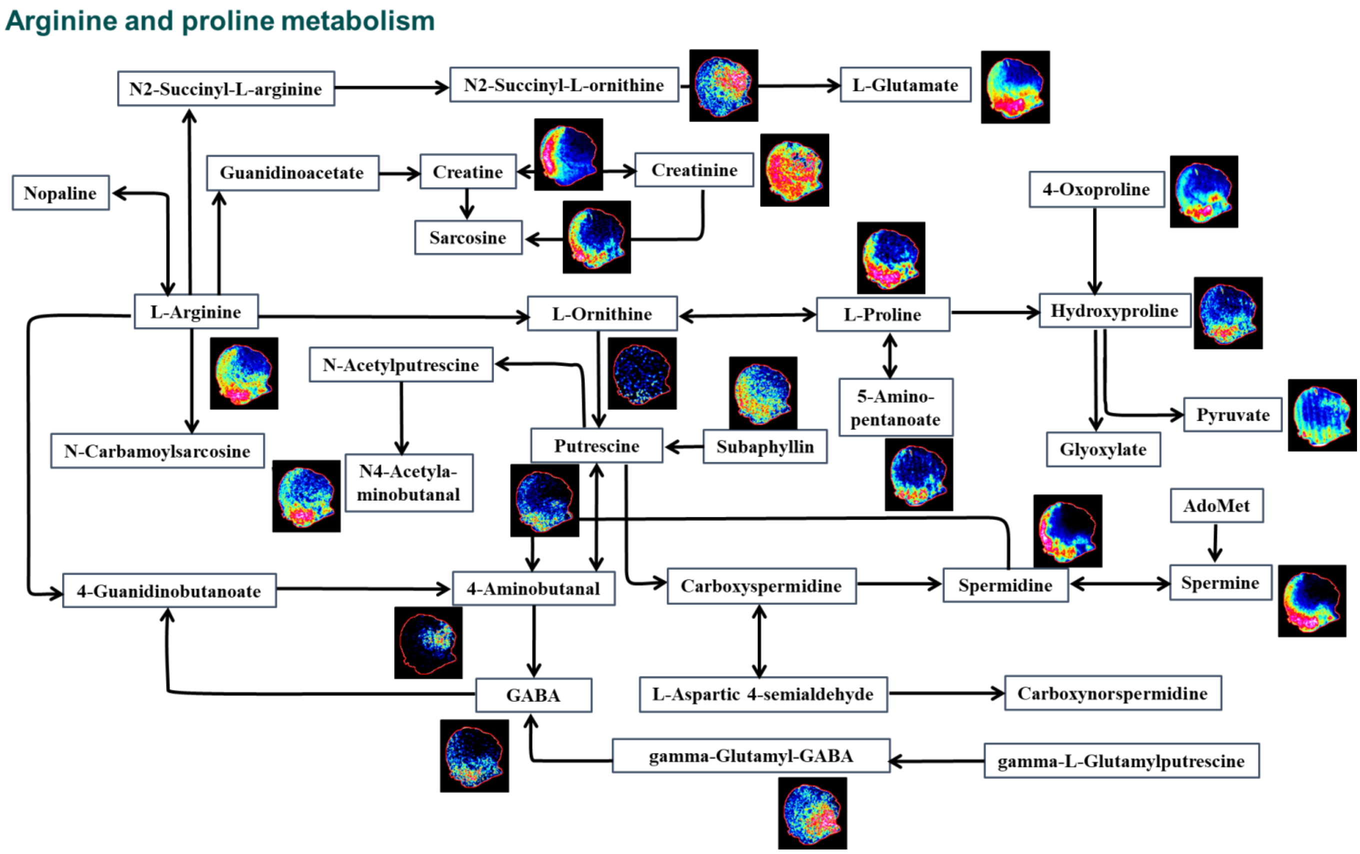

Supplementary Figure 28 | MS images of detected metabolites in arginine and proline metabolism pathway (intensity in colour scale is relative value).

Cysteine and methionine metabolism

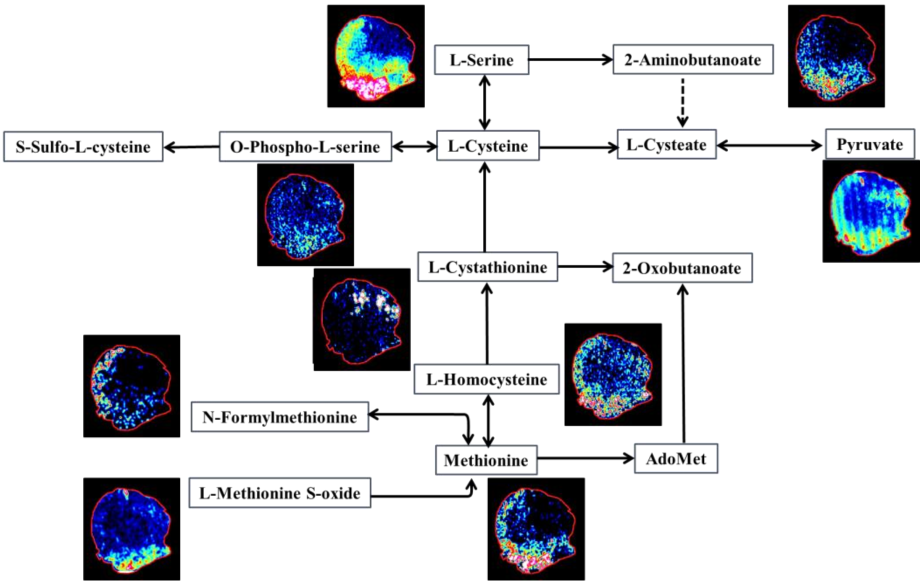

Supplementary Figure 29 | MS images of detected metabolites in cysteine and methionine metabolism pathway (intensity in colour scale is relative value).

Glycine, serine and threonine metabolism

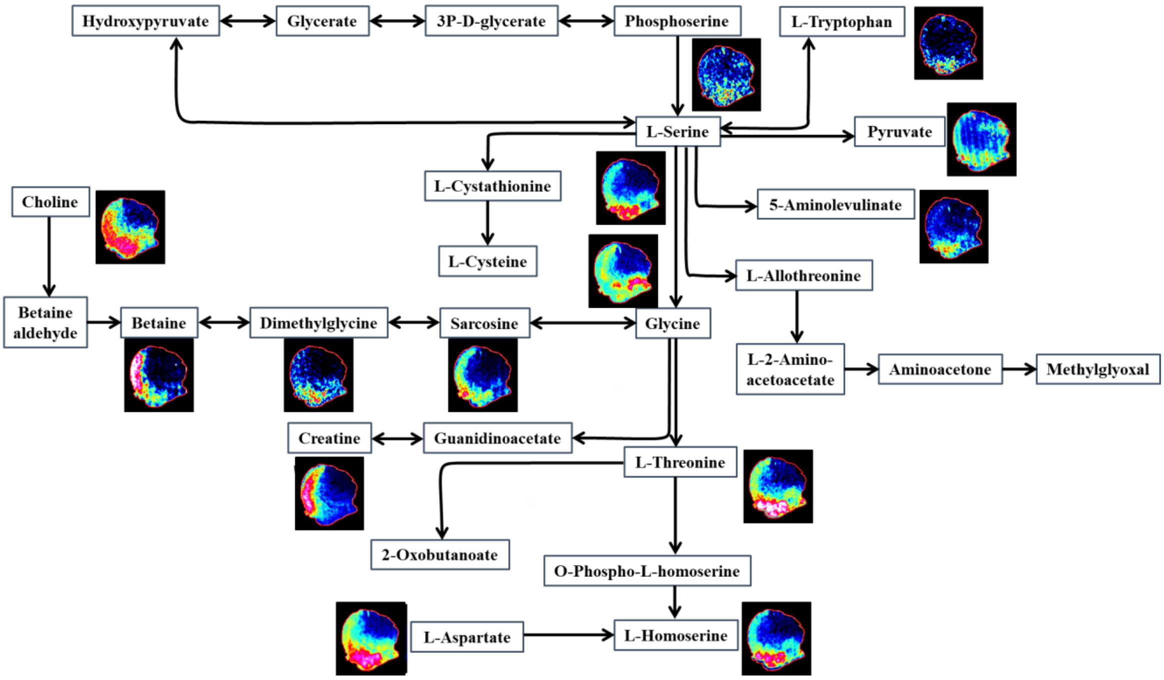

Supplementary Figure 30 | MS images of detected metabolites in glycine, serine and threonine metabolism pathway (intensity in colour scale is relative value).

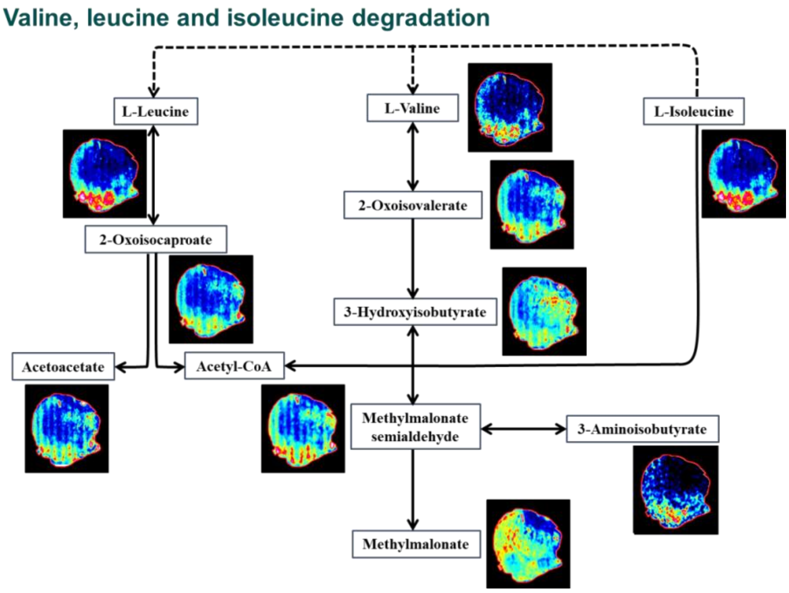

Supplementary Figure 31 | MS images of detected metabolites in valine, leucine and isoleucine degradation pathway (intensity in colour scale is relative value).

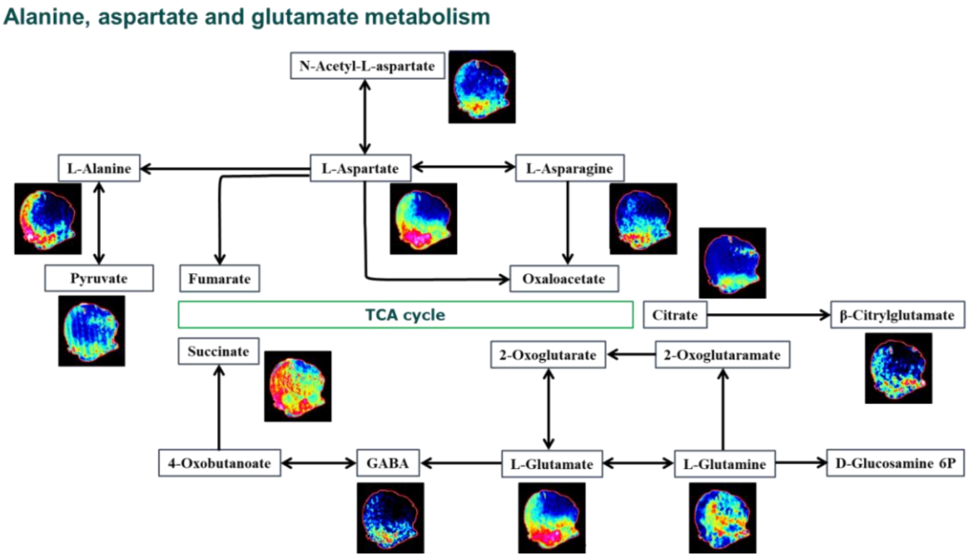

Supplementary Figure 32 | MS images of detected metabolites in alanine, aspartate and glutamate metabolism pathway (intensity in colour scale is relative value).

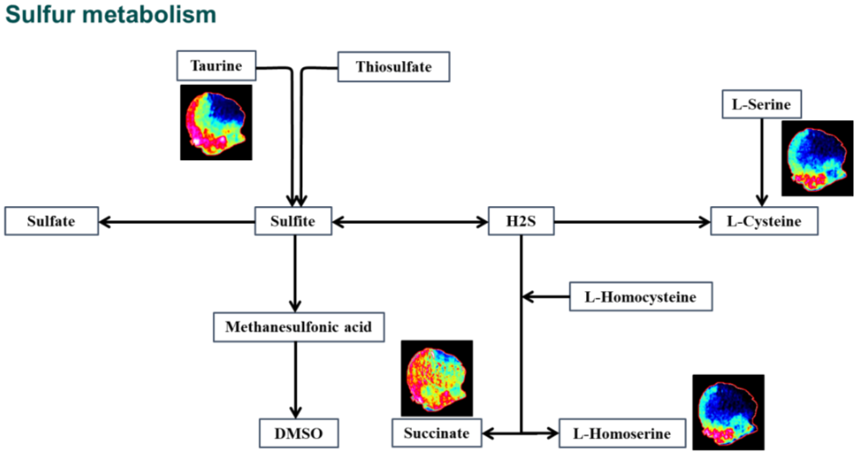

Supplementary Figure 33 | MS images of detected metabolites in sulfur metabolism pathway (intensity in colour scale is relative value).

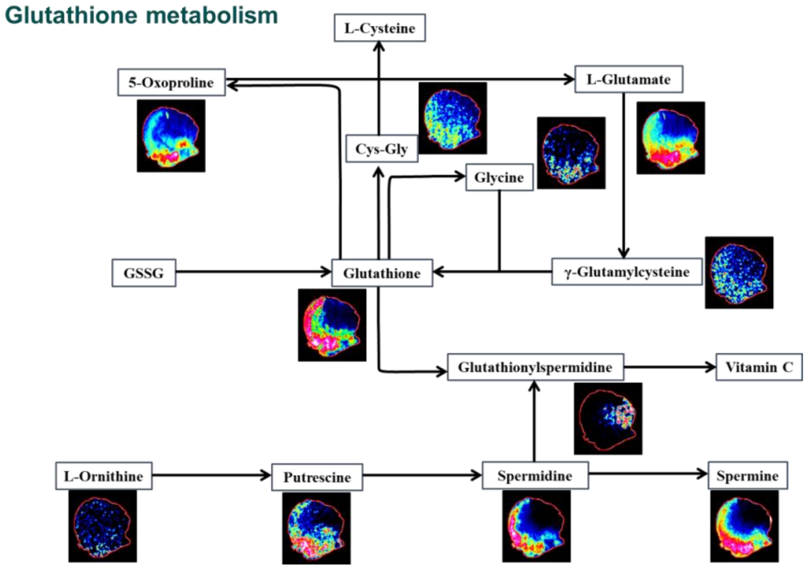

Supplementary Figure 34 | MS images of detected metabolites in glutathione metabolism pathway (intensity in colour scale is relative value).

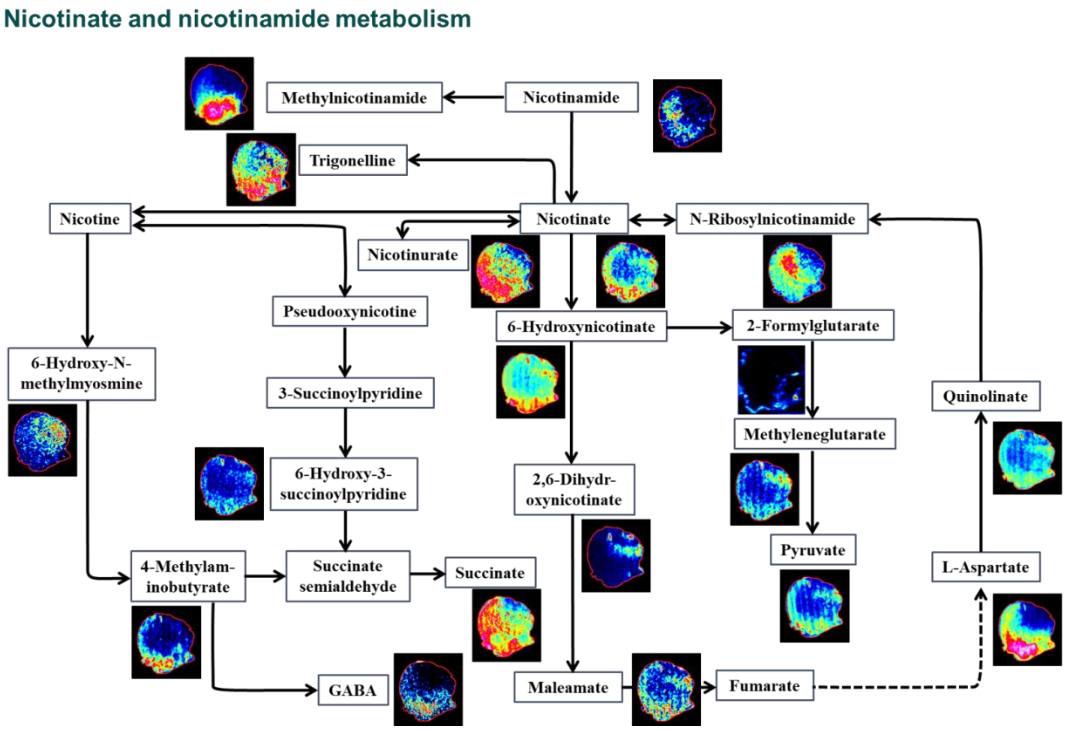

Supplementary Figure 35 | MS images of detected metabolites in nicotinate and nicotinamide metabolism pathway (intensity in colour scale is relative value).

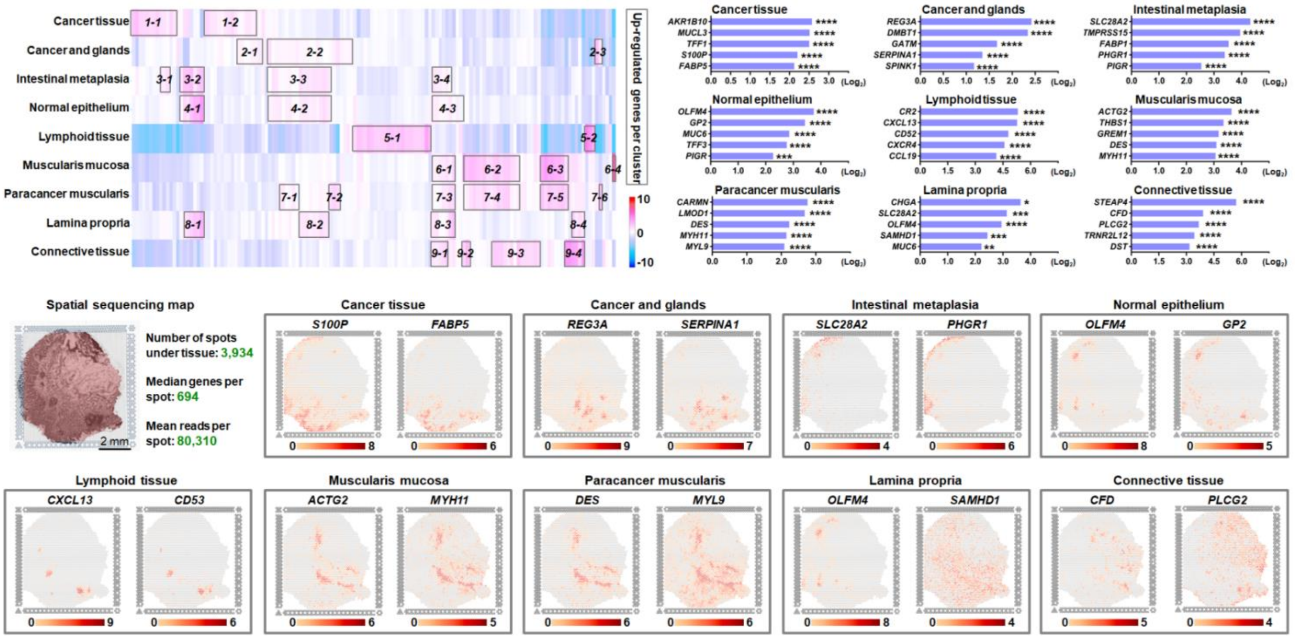

Supplementary Figure 36 | Visualization of region-specific genes in gastric cancer tissue. All scanning spots (n = 3934, the n number means the total number of spatial transcriptomics sequencing spots from patient "*No*.0429") were used for statistical analysis, ****p<0.001, ***p<0.01, **p<0.05, **p<0.05, *p<0.1, p-values are adjusted using the Benjamini-Hochberg correction for multiple tests, intensity in colour scale is log2 transformed.

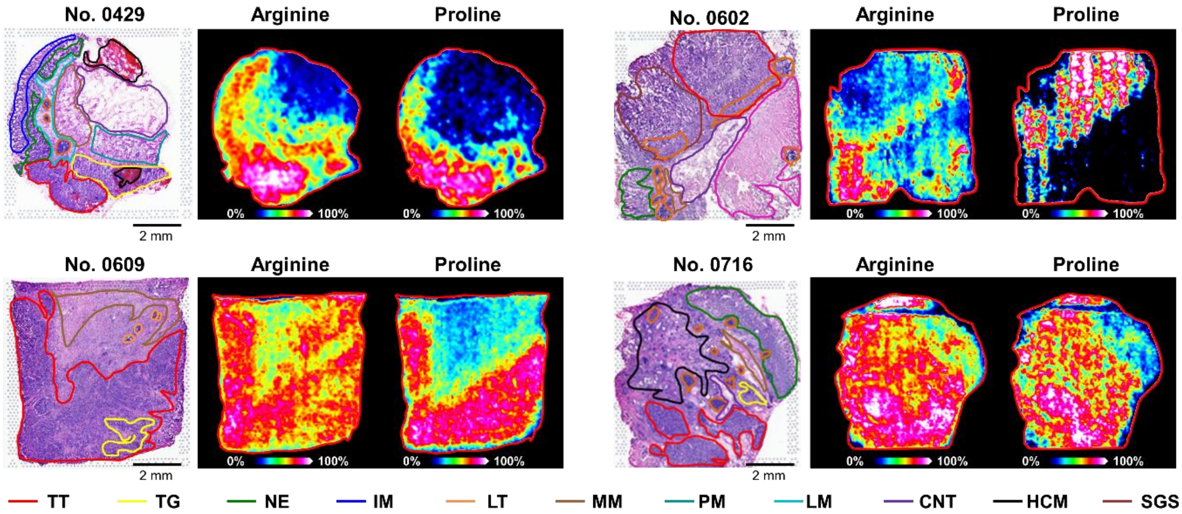

Supplementary Figure 37 | MS images of arginine and proline in gastric cancer tissue sections. TT: tumor tissue, TG: tumor and gland tissue, NE: normal epithelium, IM: intestinal metaplasia, LT: lymphoid tissue, MM: muscularis mucosa, PM: peritumoral muscularis, LM: lamina propria, CNT: connective tissue, SGS: serrated glandular structure, HCM: heterotopic cystic malformation, intensity in colour scale is relative value. Scale bar = 2 mm

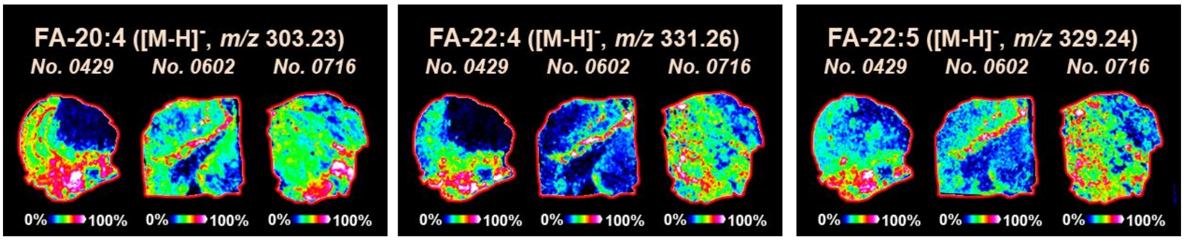

Supplementary Figure 38 | MS images of representative polyunsaturated long-chain fatty acids in gastric cancer tissue sections, intensity in colour scale is relative value.

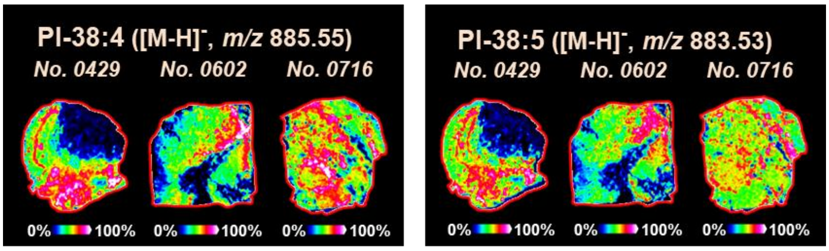

Supplementary Figure 39 | MS images of representative polyunsaturated PIs in gastric cancer tissue sections, , intensity in colour scale is relative value.

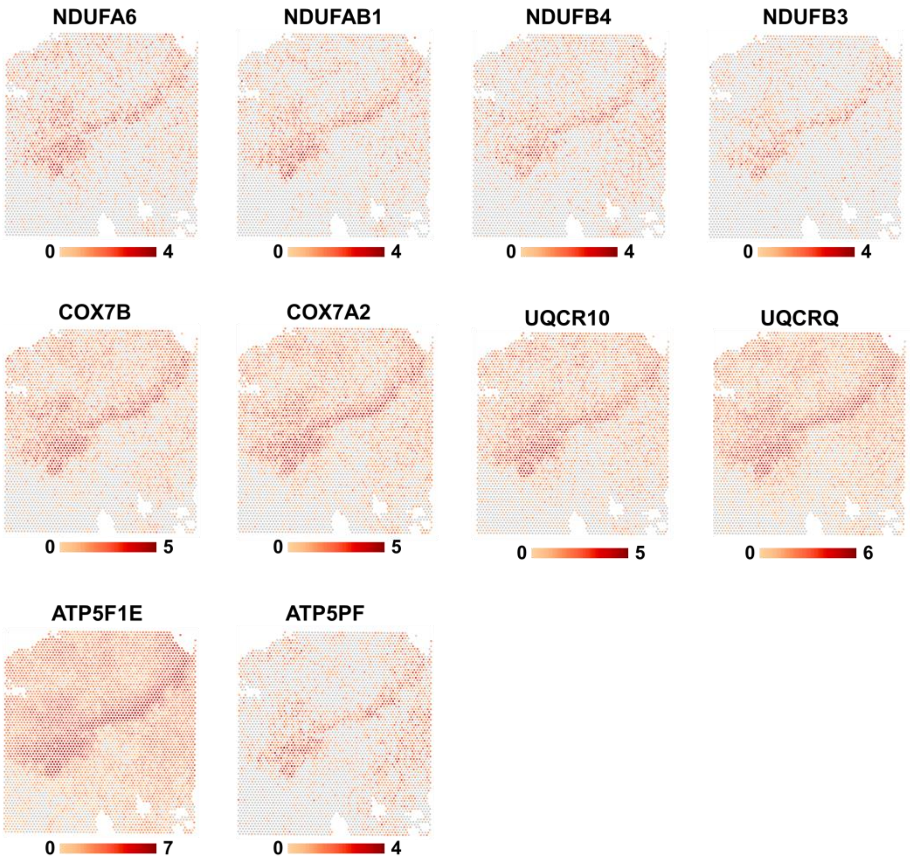

Supplementary Figure 40 | Other altered genes in oxidative phosphorylation pathway, intensity in colour scale is log2 transformed.

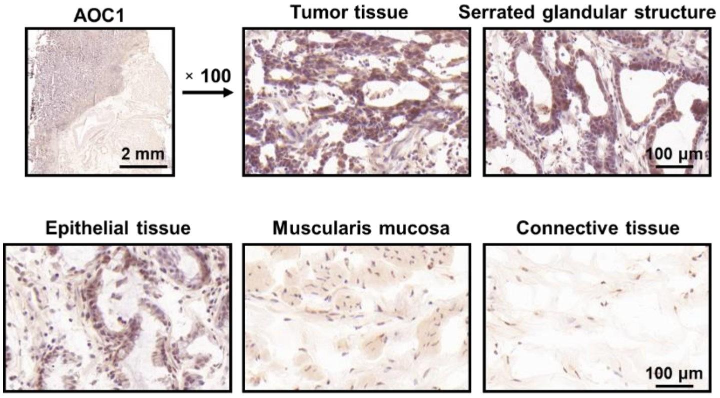

Supplementary Figure 41 | Expression of AOC1 in different tissue regions of the gastric cancer tissue section. Scale bar = 2 mm for whole tissue sections, scale bar = 100 μm for magnified image. The IHC images are representative of one patient "*No*.0602", and the experiment was repeated three times.

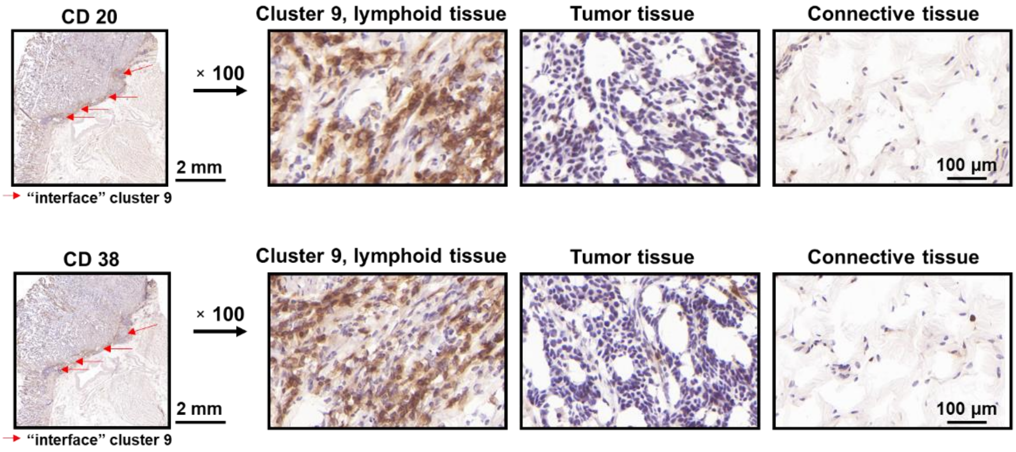

Supplementary Figure 42 | IHC stain image of CD20 and CD 38 in adjacent gastric cancer tissue sections from patient "No.0602". Scale bar = 2 mm for whole tissue sections, scale bar = 100 μm for magnified image. The IHC images are representative of one patient "*No*.0602", and the experiment was repeated three times.

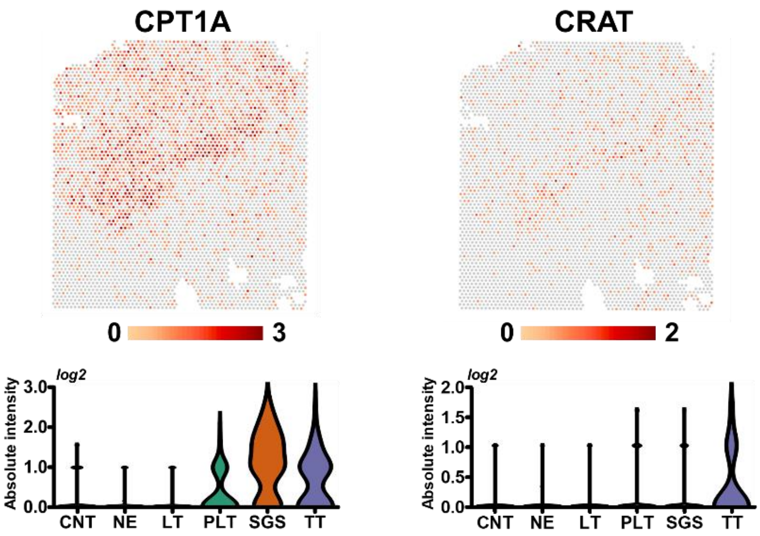

Supplementary Figure 43 | Expressions of CPT1A and CRAT genes, intensity in colour scale is log2 transformed.

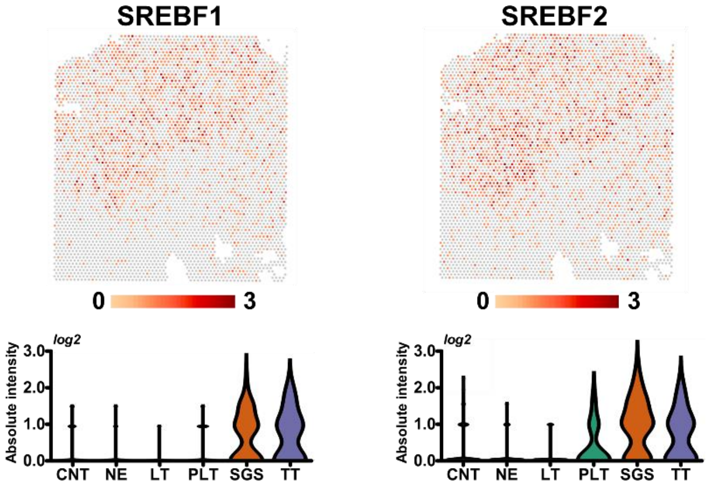

Supplementary Figure 44 | Expressions of SREBF1 and SREBF2 genes, intensity in colour scale is log2 transformed.

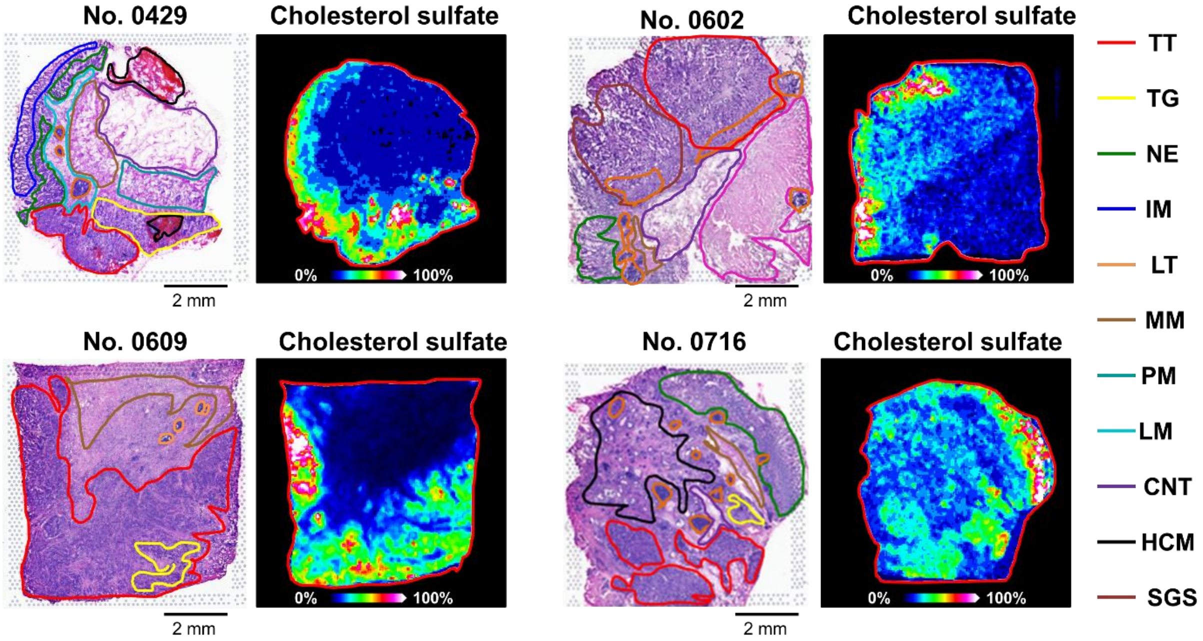

Supplementary Figure 45 | MS images of cholesterol sulfate in different gastric cancer tissue sections. TT: tumor tissue, TG: tumor and gland tissue, NE: normal epithelium, IM: intestinal metaplasia, LT: lymphoid tissue, MM: muscularis mucosa, PM: peritumoral muscularis, LM: lamina propria, CNT: connective tissue, SGS: serrated glandular structure, HCM: heterotopic cystic malformation, intensity in colour scale is relative value. Scale bar = 2 mm.

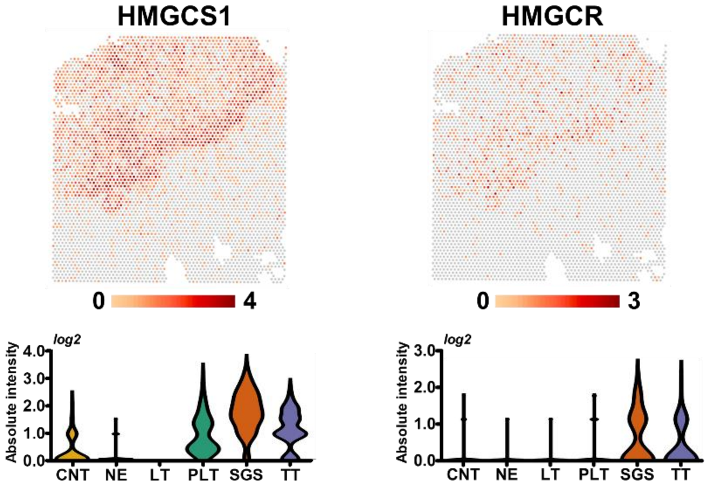

Supplementary Figure 46 | Expressions of HMGCS1 and HMGCR genes, intensity in colour scale is log2 transformed.

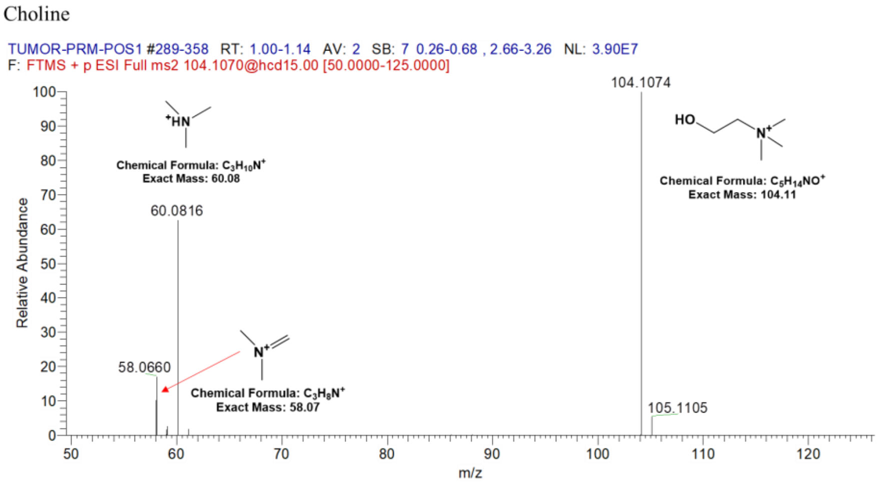

Supplementary Figure 47 | The MS/MS spectrum and the fragmentation pathway of choline.

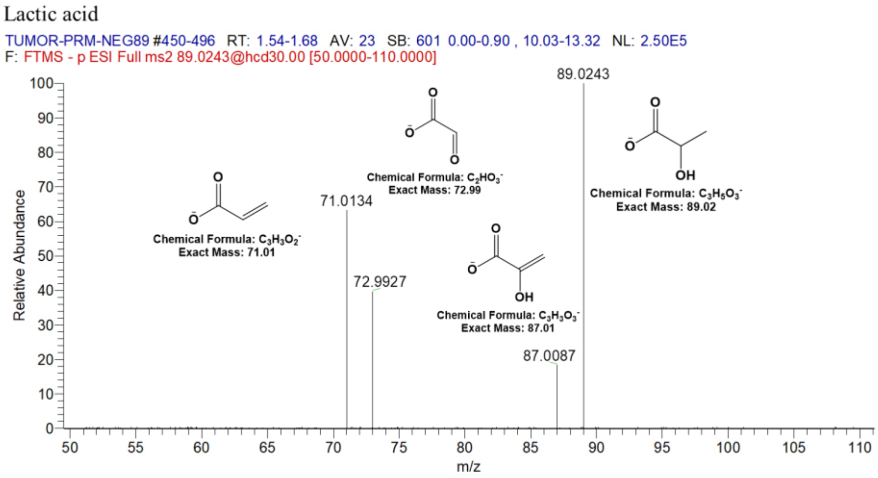

Supplementary Figure 48 | The MS/MS spectrum and the fragmentation pathway of lactic acid.

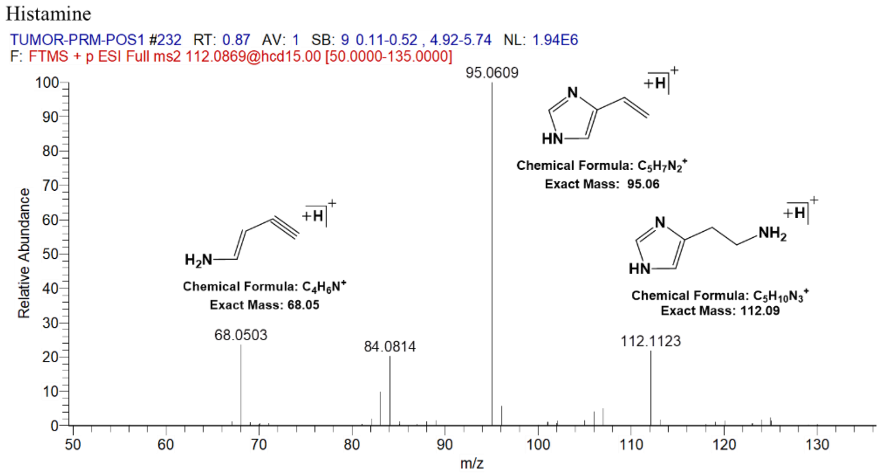

Supplementary Figure 49 | The MS/MS spectrum and the fragmentation pathway of histamine.

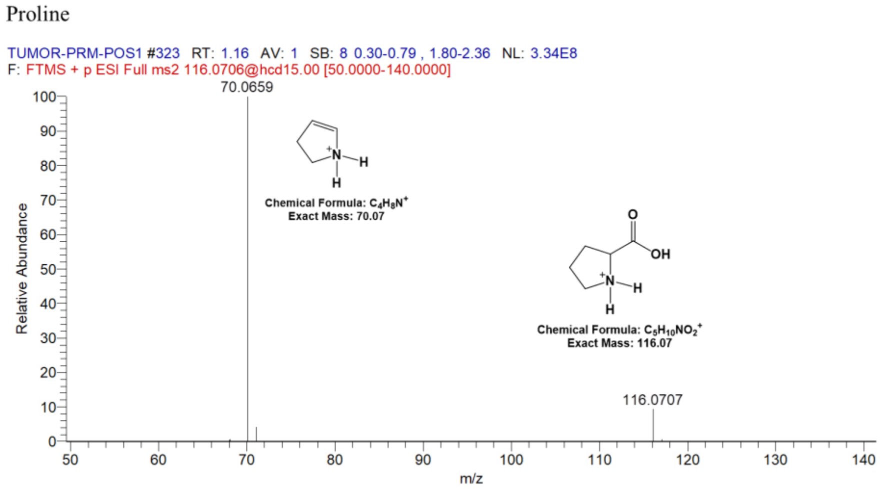

Supplementary Figure 50 | The MS/MS spectrum and the fragmentation pathway of proline.

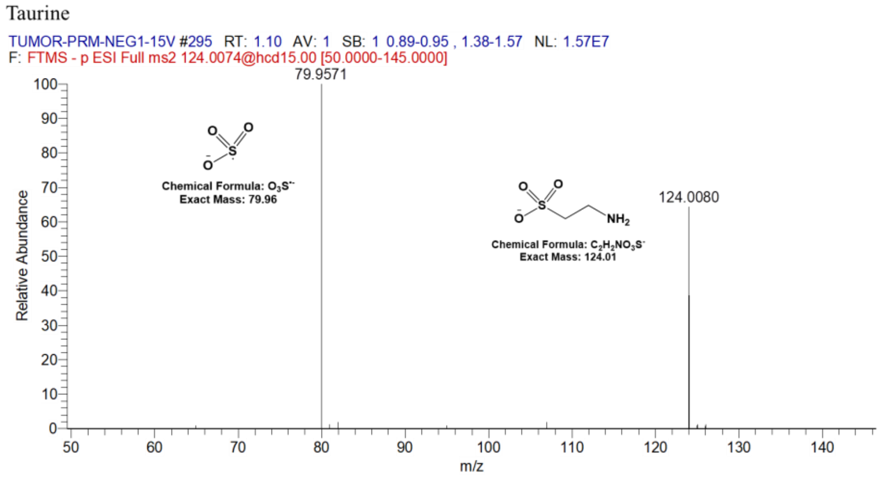

Supplementary Figure 51 | The MS/MS spectrum and the fragmentation pathway of taurine.

Malic acid

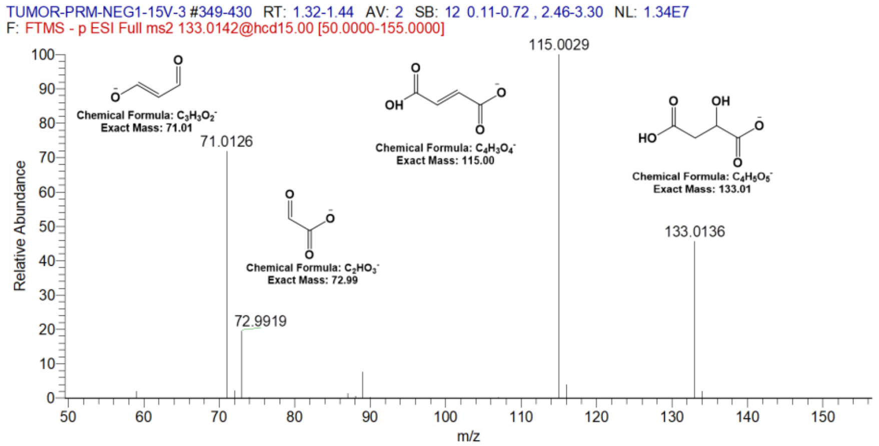

Supplementary Figure 52 | The MS/MS spectrum and the fragmentation pathway of malic acid.

Hypoxanthine

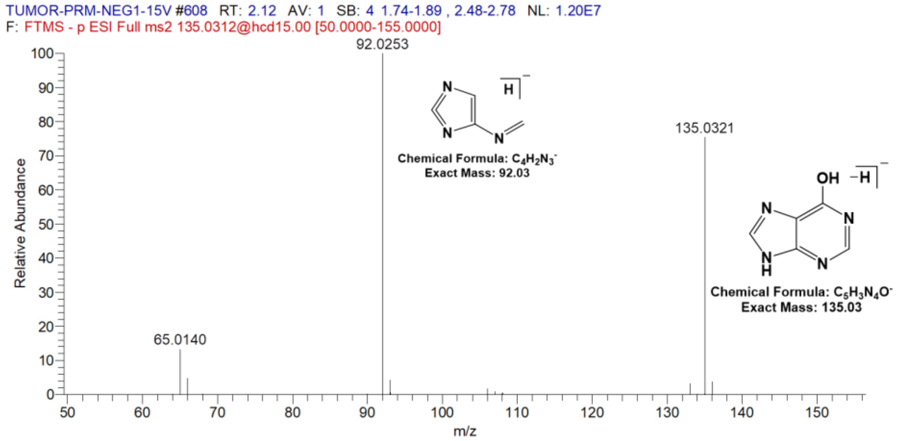

Supplementary Figure 53 | The MS/MS spectrum and the fragmentation pathway of hypoxanthine.

Glutamate

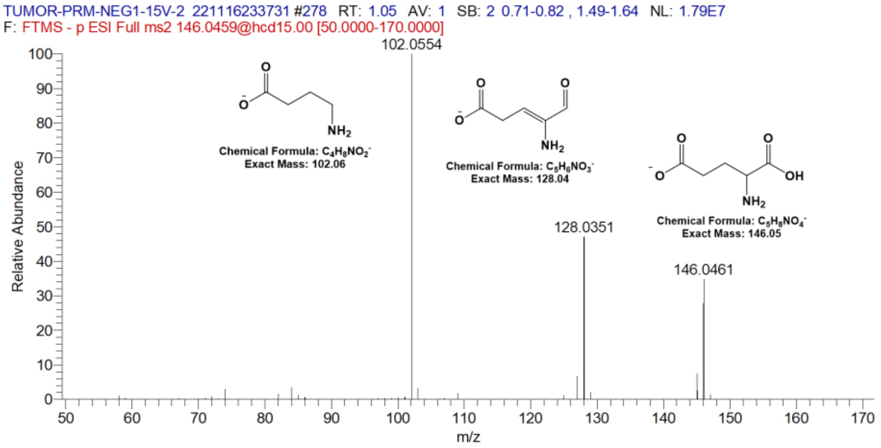

Supplementary Figure 54 | The MS/MS spectrum and the fragmentation pathway of glutamate.

Spermidine

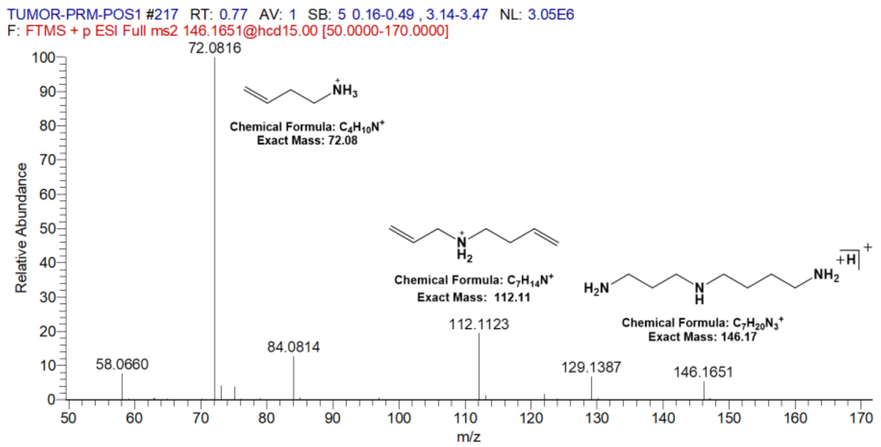

Supplementary Figure 55 | The MS/MS spectrum and the fragmentation pathway of spermidine.

Spermine

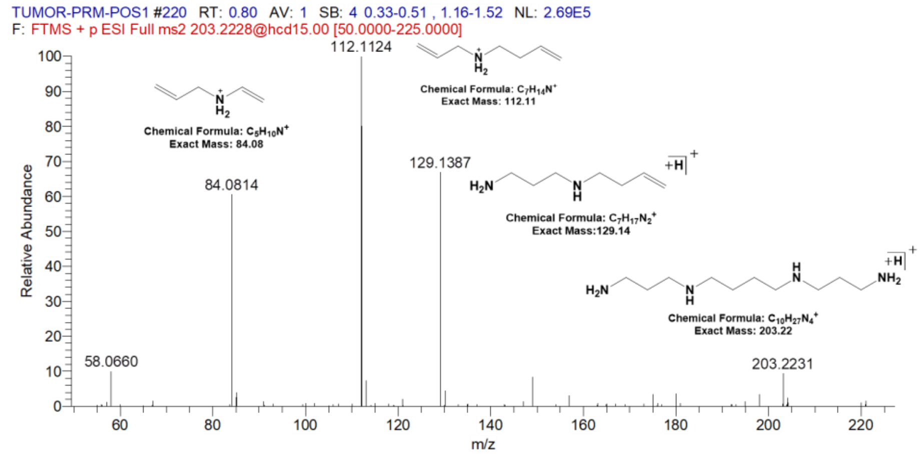

Supplementary Figure 56 | The MS/MS spectrum and the fragmentation pathway of spermine.

Histidine

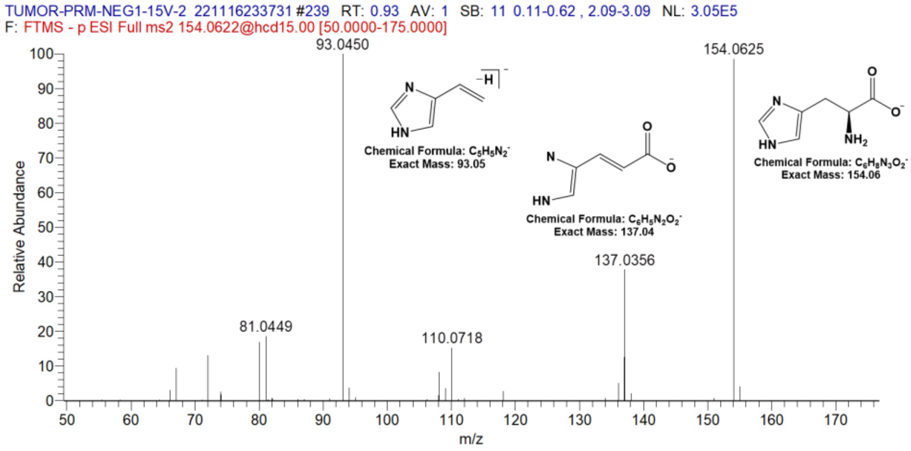

Supplementary Figure 57 | The MS/MS spectrum and the fragmentation pathway of histidine.

Arginine

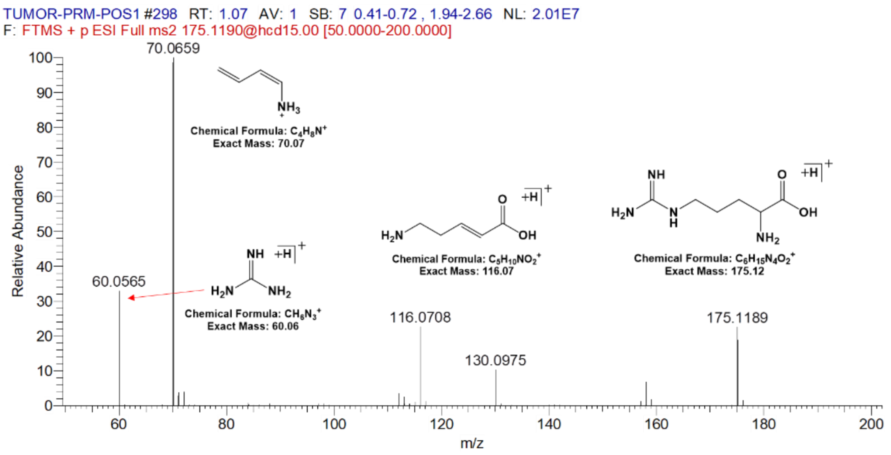

Supplementary Figure 58 | The MS/MS spectrum and the fragmentation pathway of arginine.

Phenylalanine

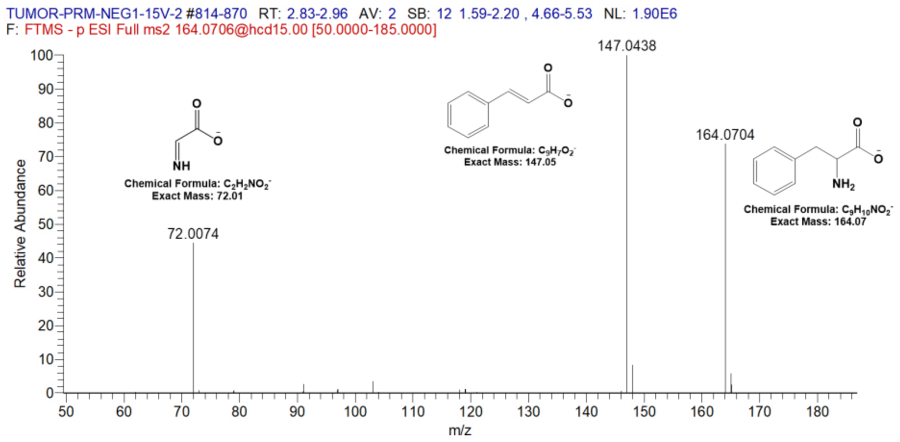

Supplementary Figure 59 | The MS/MS spectrum and the fragmentation pathway of phenylalanine.

Phosphocholine

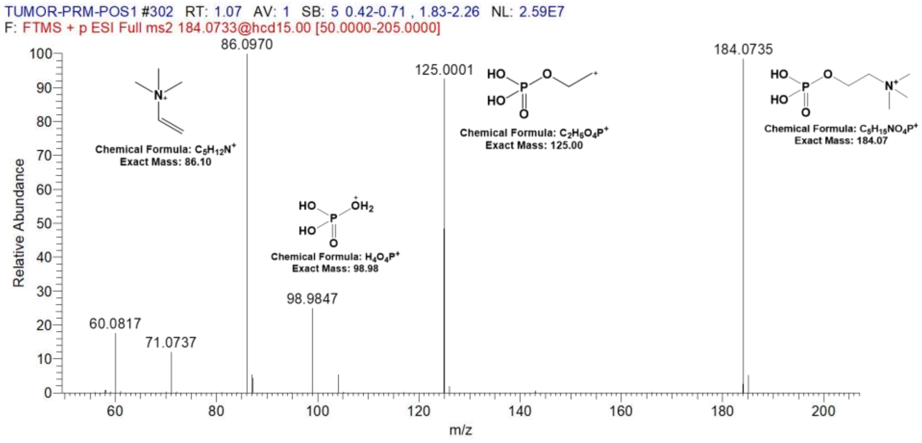

Supplementary Figure 60 | The MS/MS spectrum and the fragmentation pathway of phosphocholine.

Glycerophosphocholine

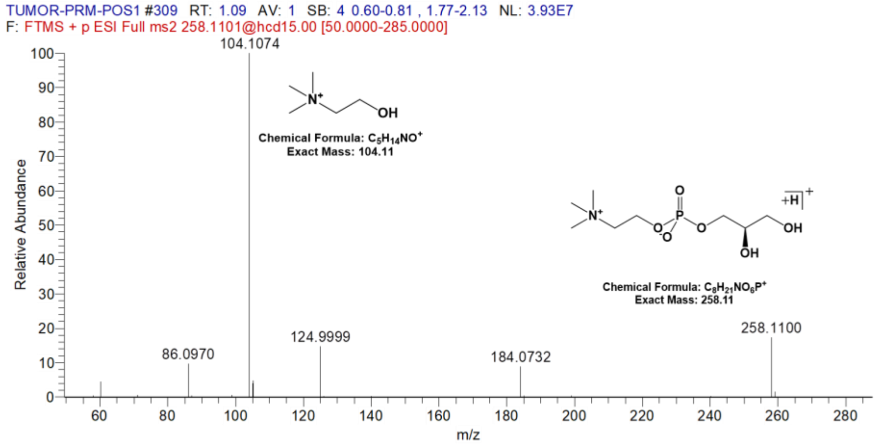

Supplementary Figure 61 | The MS/MS spectrum and the fragmentation pathway of glycerophosphocholine.

FA-16:0

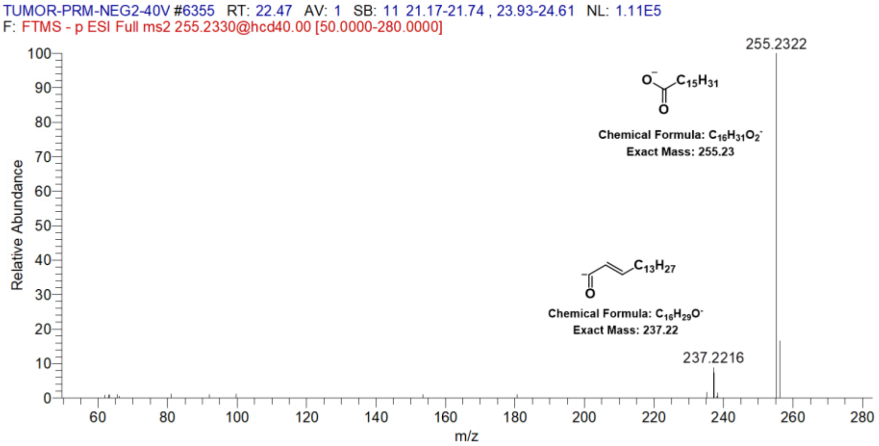

Supplementary Figure 62 | The MS/MS spectrum and the fragmentation pathway of FA-16:0.

FA-18:2

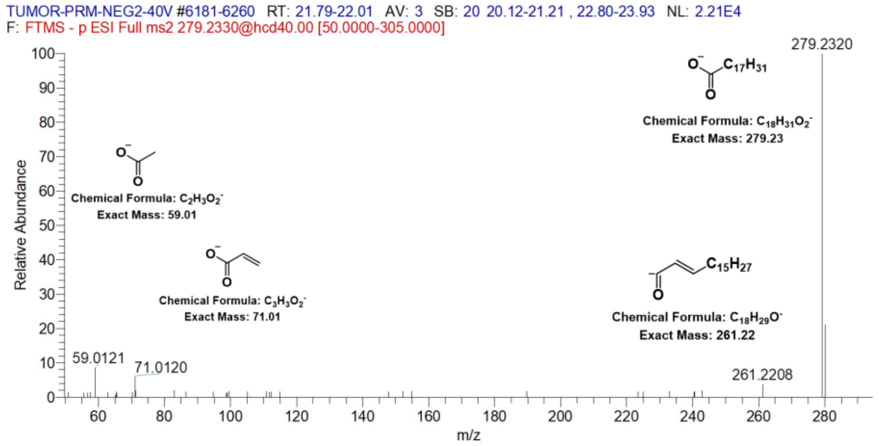

Supplementary Figure 63 | The MS/MS spectrum and the fragmentation pathway of FA-18:2.

FA-20:5

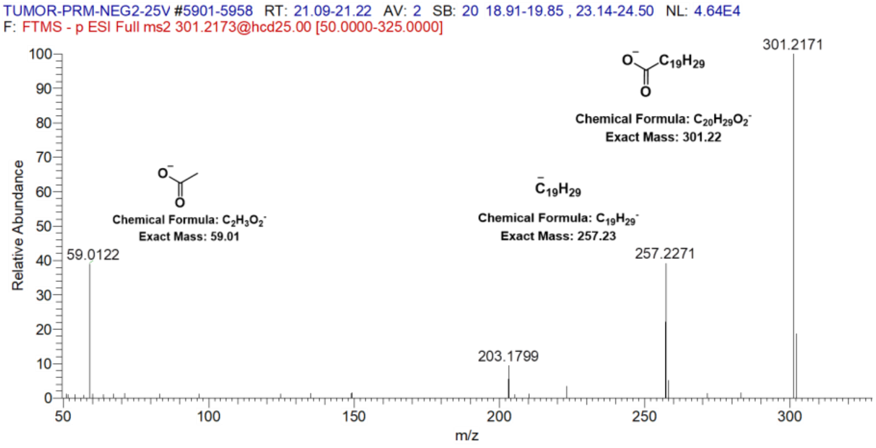

Supplementary Figure 64 | The MS/MS spectrum and the fragmentation pathway of FA-20:5.

FA-20:4

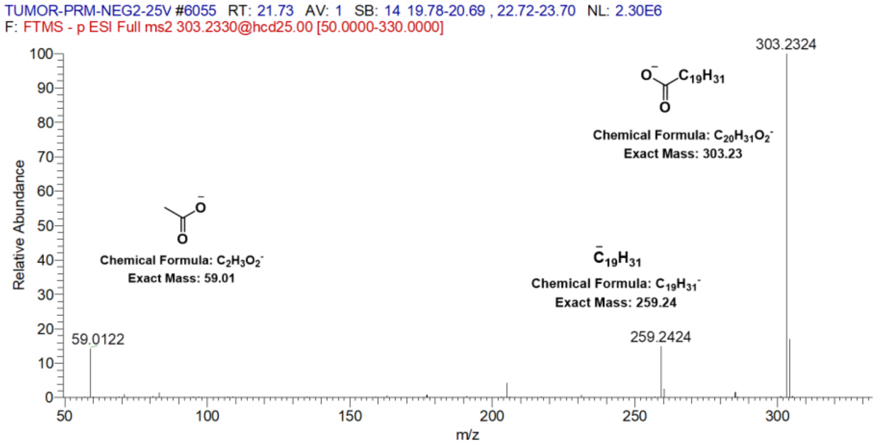

Supplementary Figure 65 | The MS/MS spectrum and the fragmentation pathway of FA-20:4.

FA-22:6

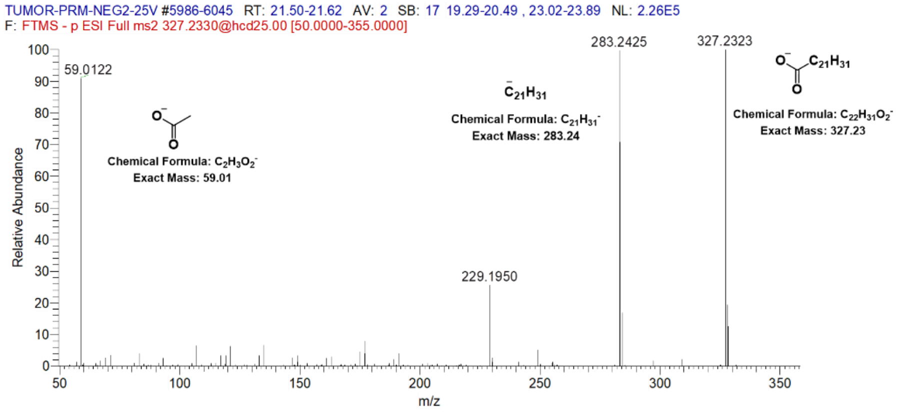

Supplementary Figure 66 | The MS/MS spectrum and the fragmentation pathway of FA-22:6.

FA-22:5

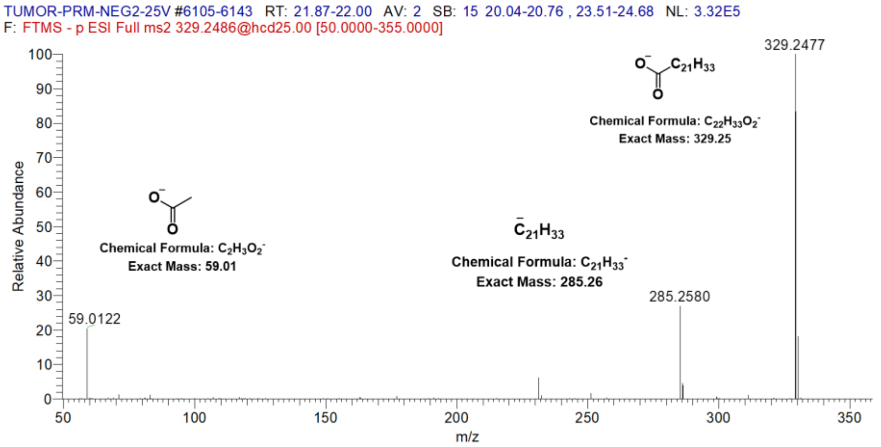

Supplementary Figure 67 | The MS/MS spectrum and the fragmentation pathway of FA-22:5.

Lyso PC-16:0

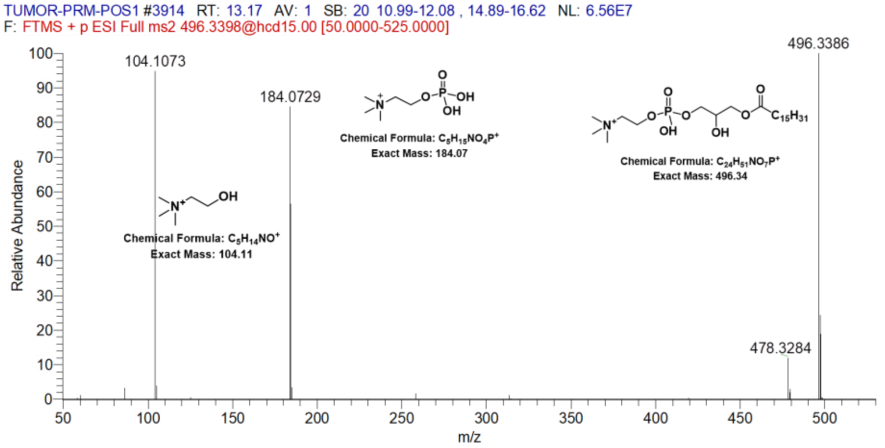

Supplementary Figure 68 | The MS/MS spectrum and the fragmentation pathway of lysoPC-16:0.

PC-32:0

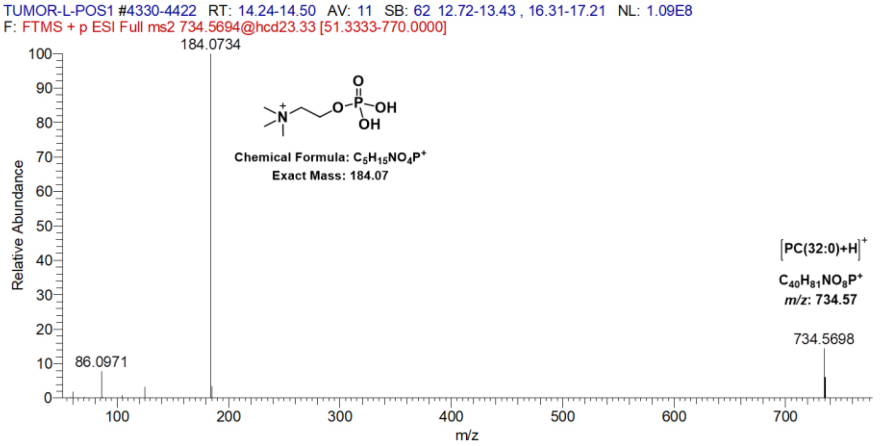

Supplementary Figure 69 | The MS/MS spectrum and the fragmentation pathway of PC-32:0.

PC-34:1

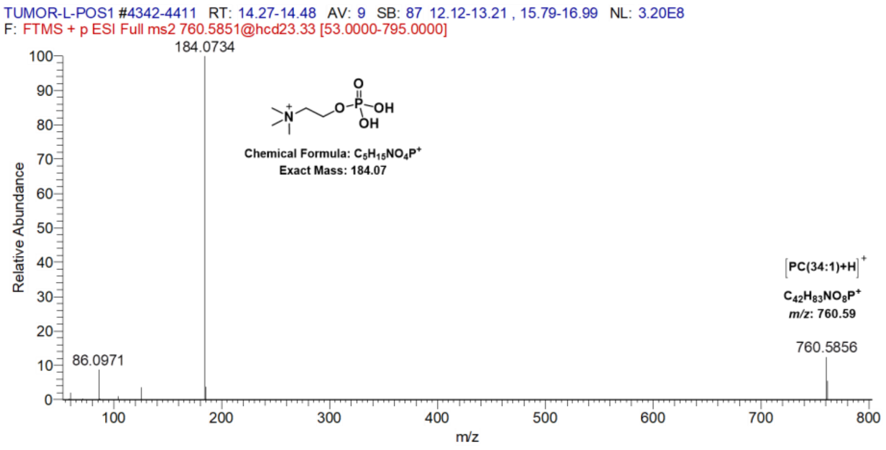

Supplementary Figure 70 | The MS/MS spectrum and the fragmentation pathway of PC-34:1.

PC-38:4

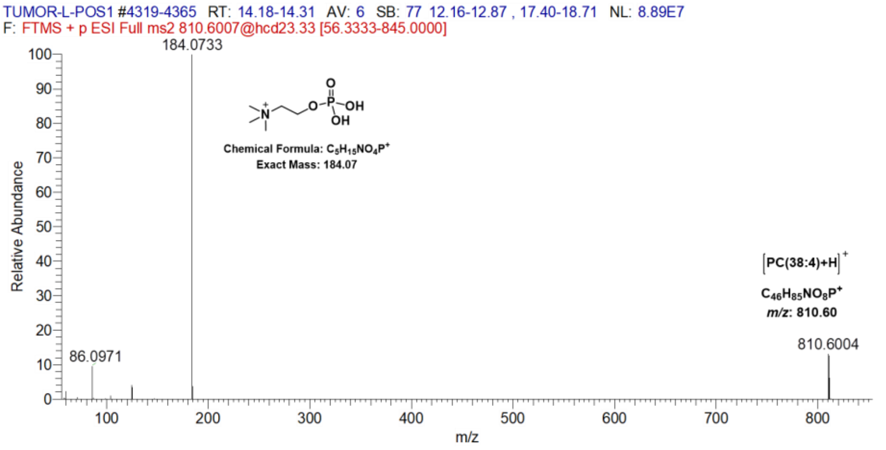

Supplementary Figure 71 | The MS/MS spectrum and the fragmentation pathway of PC-38:4.

PE-34:0

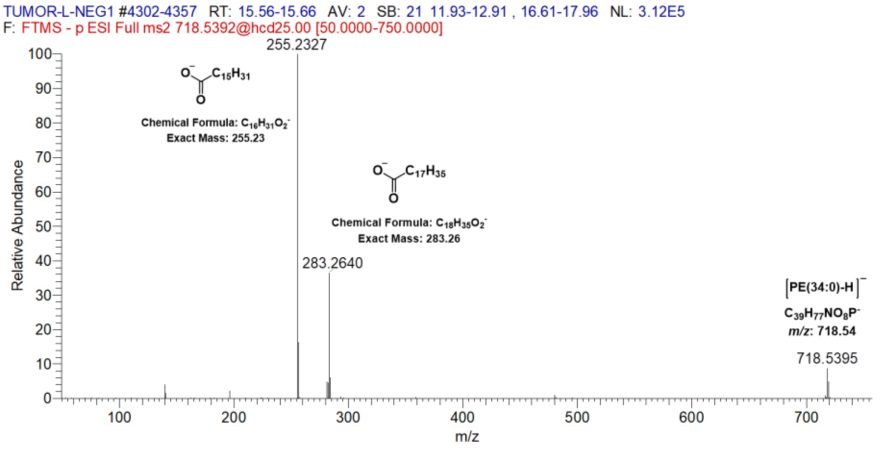

Supplementary Figure 72 | The MS/MS spectrum and the fragmentation pathway of PE-34:0.

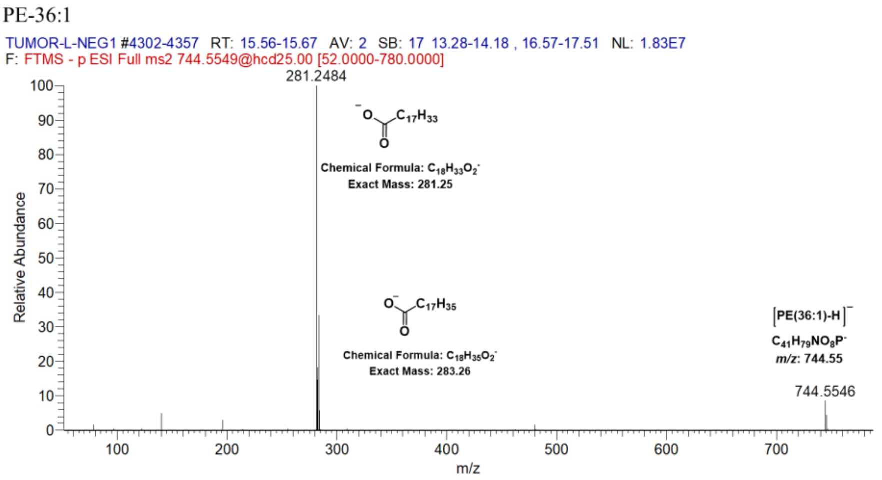

Supplementary Figure 73 | The MS/MS spectrum and the fragmentation pathway of PE-36:1.

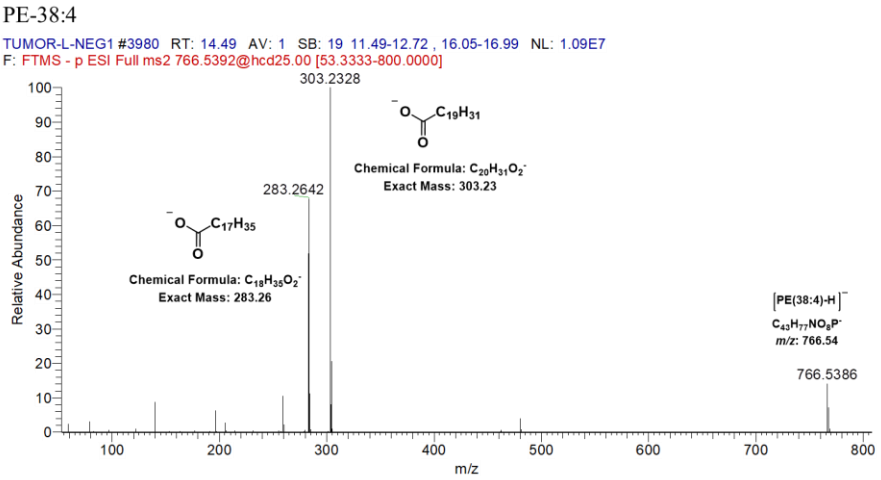

Supplementary Figure 74 | The MS/MS spectrum and the fragmentation pathway of PE-38:4.

Supplementary Table 1 | Annotated metabolites among the metabolic pathways analyzed in this study.

**Name**	**Formula**	**Ion type**	**Theoretical *m/z***	**Measured *m/z***	**Relative error (ppm)**
Choline	$C_5H_{13}NO$	$[M+H]^+$	104.107	104.1066	-3.85
Histamine	$C_5H_9N_3$	$[M+H]^+$	112.0869	112.0865	-3.57
Proline	$C_5H_9NO_2$	$[M+H]^+$	116.0706	116.0702	-3.45
Spermine	$C_{10}H_{26}N_4$	$[M+H]^+$	203.2228	203.2225	-1.48
Spermidine	$C_7H_{19}N_3$	$[M+H]^+$	146.1651	146.1648	-2.05
Arginine	$C_6H_{14}N_4O_2$	$[M+H]^+$	175.119	175.1185	-2.86
L-Carnitine*	C7H15NO3	[M+H]+	162.1122	162.112	-1.23
Betaine*	C5H11NO2	[M+H]+	118.0863	118.0860	-2.54

Phosphocholine	$C_5H_{14}NO_4P$	$[M+H]^+$	184.0733	184.0729	-2.17
Glycerophos phocholine	$C_8H_{20}NO_6P$	$[M+H]^+$	258.1101	258.1092	-3.49
Lyso PC-16:0	$C_{24}H_{50}NO_7P$	$[M+H]^+$	496.3398	496.3386	-2.42
Lyso PC-18:0	$C_{26}H_{54}NO_7P$	$[M+H]^+$	524.3711	524.3694	-3.24
Lyso PC-18:1	$C_{26}H_{52}NO_7P$	$[M+H]^+$	522.3554	522.3538	-3.06
PC-32:0	$C_{40}H_{80}NO_8P$	$[M+H]^+$	734.5694	734.5664	-4.09
PC-34:1	$C_{42}H_{82}NO_8P$	$[M+H]^+$	760.5851	760.5815	-4.74
PC-34:2	$C_{42}H_{80}NO_8P$	$[M+H]^+$	758.5694	758.5662	-4.22
PC-36:2	$C_{44}H_{84}NO_8P$	$[M+H]^+$	786.6007	786.5981	-3.31
PC-38:4	$C_{46}H_{84}NO_8P$	$[M+H]^+$	810.6007	810.5972	-4.32
Lactic acid	$C_3H_6O_3$	$[M-H]^-$	89.0244	89.0240	-4.49
Taurine	$C_2H_7NO_3S$	$[M-H]^-$	124.0074	124.0068	-4.84
Malic acid	$C_4H_5O_5$	$[M-H]^-$	133.0142	133.0136	-4.51
Hypoxanthine	$C_5H_4N_4O$	$[M-H]^-$	135.0312	135.0306	-4.44
Glutamate	$C_5H_9NO_4$	$[M-H]^-$	146.0459	146.0452	-4.79
Histidine	$C_6H_9N_3O_2$	$[M-H]^-$	154.0622	154.0615	-4.55
Phenylalanine	$C_9H_{11}NO_2$	$[M-H]^-$	164.0706	164.0709	1.83
Glucose-phosphate	$C_6H_{13}O_9P$	$[M-H]^-$	259.0224	259.0217	-2.70
FA-16:1	$C_{16}H_{30}O_2$	$[M-H]^-$	253.2173	253.2163	-3.95
FA-18:1	$C_{18}H_{34}O_2$	$[M-H]^-$	281.2486	281.2473	-4.63
FA-20:1	$C_{20}H_{38}O_2$	$[M-H]^-$	309.2799	309.2785	-4.53
FA-22:1	$C_{22}H_{42}O_2$	$[M-H]^-$	337.3112	337.3096	-4.75
FA-24:1	$C_{14}H_{26}O_2$	$[M-H]^-$	365.3425	365.3408	-4.66
FA-16:0	$C_{16}H_{32}O_2$	$[M-H]^-$	255.2330	255.2319	-4.31
FA-18:0	$C_{18}H_{36}O_2$	$[M-H]^-$	283.2643	283.2630	-4.59
FA-20:0	$C_{20}H_{40}O_2$	$[M-H]^-$	311.2956	311.2942	-4.50
FA-22:5	$C_{22}H_{34}O_2$	$[M-H]^-$	329.2486	329.2471	-4.56
FA-22:6	$C_{22}H_{32}O_2$	$[M-H]^-$	327.2330	323.2317	-3.96
FA-20:5	$C_{20}H_{30}O_2$	$[M-H]^-$	301.2173	301.2161	-3.99
FA-18:2	$C_{18}H_{32}O_2$	$[M-H]^-$	279.2330	279.2317	-4.66
FA-20:4	$C_{20}H_{32}O_2$	$[M-H]^-$	303.2330	303.2320	-3.30
PE-36:2	$C_{41}H_{78}NO_8P$	$[M-H]^-$	742.5392	742.5377	-2.02
PE-36:1	$C_{41}H_{80}NO_8P$	$[M-H]^-$	744.5549	744.5518	-4.17

PE-34:1	$C_{39}H_{76}NO_8P$	$[M-H]^-$	716.5235	716.5215	-2.79
PE-34:0	$C_{39}H_{78}NO_8P$	$[M-H]^-$	718.5392	718.5360	-4.46
PE-38:6	$C_{43}H_{74}NO_8P$	$[M-H]^-$	762.5079	762.5066	-1.71
PE-38:4	$C_{43}H_{78}NO_8P$	$[M-H]^-$	766.5392	766.5375	-2.22
PI-36:4	$C_{45}H_{79}O_{13}P$	$[M-H]^-$	857.5186	857.5160	-3.03
PI-38:5	$C_{47}H_{81}O_{13}P$	$[M-H]^-$	883.5342	883.5314	-3.17
PI-38:4	$C_{47}H_{83}O_{13}P$	$[M-H]^-$	885.5499	885.5479	-2.26
PI-36:1	$C_{45}H_{85}O_{13}P$	$[M-H]^-$	863.5655	863.5651	-0.46
PI-34:1	$C_{43}H_{81}O_{13}P$	$[M-H]^-$	835.5342	835.5316	-3.11
PS-36:2	$C_{42}H_{78}NO_{10}P$	$[M-H]^-$	786.5291	786.5277	-1.78
PS-36:1	$C_{42}H_{80}NO_{10}P$	$[M-H]^-$	788.5447	788.5430	-2.16
PS-38:4	$C_{44}H_{78}NO_{10}P$	$[M-H]^-$	810.5291	810.5263	-3.46
PS-38:6	$C_{44}H_{74}NO_{10}P$	$[M-H]^-$	806.4978	806.4951	-3.35
PG-38:4	$C_{44}H_{79}O_{10}P$	$[M-H]^-$	797.5338	797.5301	-4.64
PG-38:5	$C_{44}H_{77}O_{10}P$	$[M-H]^-$	795.5182	795.5156	-3.27
PG-38:3	$C_{44}H_{81}O_{10}P$	$[M-H]^-$	799.5495	799.5482	-1.63
PG-36:4	$C_{42}H_{75}O_{10}P$	$[M-H]^-$	769.5025	769.5010	-1.95
C24:1-OH-ST	$C_{48}H_{91}NO_{12}S$	$[M-H]^-$	904.6189	904.6180	-0.99
C24:0-OH-ST	$C_{48}H_{93}NO_{12}S$	$[M-H]^-$	906.6346	906.6324	-2.42
