# Supplementary material for: Spatially resolved multi-omics highlights cell-specific metabolic remodeling and interactions in gastric cancer
Source: Nat Commun. 2023 May 10;14:2692. doi: 10.1038/s41467-023-38360-5 (PMC10172194; doi:10.1038/s41467-023-38360-5)
Supplement: Supplementary file 2 — Description of Additional Supplementary Files [file 41467_2023_38360_MOESM2_ESM.pdf]

Description of Additional Supplementary Files

File Name: Supplementary Data 1

Description: expression levels of representative lipids in different micro-regions of gastric cancer tissue from patient No.0429.

File Name: Supplementary Data 2

Description: expression levels of glucose, glucose-phosphate, lactic acid, succinic acid, malic acid, histidine, histamine, FA-18:1, Lyso-PC-16:1, C26:2-OH-SFT, C22:0-OH-SFT, C22:1-OH-SFT, C24:0-OH-SFT, and C24:1-OH-SFT in normal epithelium (NE), serrated glandular structure (SGS), and tumor tissue of gastric cancer tissue section.

File Name: Supplementary Data 3

Description: screened genes and pathways that enriched in serrated glandular structure (SGS) tissue.

File Name: Supplementary Data 4

Description: marker genes for the cell cluster annotation of gastric cancer tissue.

File Name: Supplementary Data 5

Description: expression levels of glutamine, glutamate arachidonic acid, docosahexaenoic acid, docosapentaenoic acid, and docosatetraenoic acid in different micro-regions of gastric cancer tissue from patient No.0406.

File Name: Supplementary Data 6

Description: the raw values for the maximum tumour volume.
