# Supplementary material for: Spatially resolved multi-omics highlights cell-specific metabolic remodeling and interactions in gastric cancer
Source: Nat Commun. 2023 May 10;14:2692. doi: 10.1038/s41467-023-38360-5 (PMC10172194; doi:10.1038/s41467-023-38360-5)
Supplement: Supplementary file 9 — Reporting Summary [file 41467_2023_38360_MOESM9_ESM.pdf]

Corresponding author(s): Jiuming He, Zhaode Bu, Wentai Dai, Jiafu Ji

Last updated by author(s): Apr 19, 2023

Reporting Summary

Nature Portfolio wishes to improve the reproducibility of the work that we publish. This form provides structure for consistency and transparency in reporting. For further information on Nature Portfolio policies, see our Editorial Policies and the Editorial Policy Checklist.

Statistics

For all statistical analyses, confirm that the following items are present in the figure legend, table legend, main text, or Methods section.

n/a	Confirmed	
☐	☒	The exact sample size (*n*) for each experimental group/condition, given as a discrete number and unit of measurement
☐	☒	A statement on whether measurements were taken from distinct samples or whether the same sample was measured repeatedly
☐	☒	The statistical test(s) used AND whether they are one- or two-sided<br>*Only common tests should be described solely by name; describe more complex techniques in the Methods section.*
☒	☐	A description of all covariates tested
☐	☒	A description of any assumptions or corrections, such as tests of normality and adjustment for multiple comparisons
☐	☒	A full description of the statistical parameters including central tendency (e.g. means) or other basic estimates (e.g. regression coefficient) AND variation (e.g. standard deviation) or associated estimates of uncertainty (e.g. confidence intervals)
☐	☒	For null hypothesis testing, the test statistic (e.g. *F*, *t*, *r*) with confidence intervals, effect sizes, degrees of freedom and *P* value noted<br>*Give P values as exact values whenever suitable.*
☒	☐	For Bayesian analysis, information on the choice of priors and Markov chain Monte Carlo settings
☒	☐	For hierarchical and complex designs, identification of the appropriate level for tests and full reporting of outcomes
☒	☐	Estimates of effect sizes (e.g. Cohen's *d*, Pearson's *r*), indicating how they were calculated

Our web collection on statistics for biologists contains articles on many of the points above.

Software and code

Policy information about availability of computer code

Data collection	No software was used for data collection.
Data analysis	MassImager Pro 1.0 software was used for AFADESI-MS data imaging. SCiLS Lab 2018b software was used for MALDI-MS data imaging. Loupe Browser 6 software was used for spatial transcriptomics analysis. KEGG pathway enrichment analyses of DEGs were performed by KOBAS-i. MetaboAnalyst 5.0 was used to perform metabolic pathway analysis

For manuscripts utilizing custom algorithms or software that are central to the research but not yet described in published literature, software must be made available to editors and reviewers. We strongly encourage code deposition in a community repository (e.g. GitHub). See the Nature Portfolio guidelines for submitting code & software for further information.

Data

Policy information about availability of data

All manuscripts must include a data availability statement. This statement should provide the following information, where applicable:

- Accession codes, unique identifiers, or web links for publicly available datasets
- A description of any restrictions on data availability
- For clinical datasets or third party data, please ensure that the statement adheres to our policy

Sequencing and spatial transcriptomics data generated in this study have been deposited in the GSA (Genome Sequence Archive)-human database of the National Genomics Data Center under accession number HRA003070 [https://ngdc.cncb.ac.cn/gsa-human/browse/HRA003070]. The raw ADADESI-MS data and MALDI-MS

data generated in this study have been deposited in the OMIX database of the National Genomics Data Center under accession number OMIX002397 [https://ngdc.cncb.ac.cn/omix/release/OMIX002397]. ADADESI-MS imaging and MALDI-MS imaging data have been deposited in the Metaspace database which allows visualization of mass spectrometry imaging results, and all the MS imaging data can be directly download from [https://metaspace2020.eu/datasets?q=710b1782-343a-11ed-89bf-738830e26a67]. The remaining data are available within the Article, Supplementary Information or Source Data file.

Human research participants

Policy information about studies involving human research participants and Sex and Gender in Research.

Reporting on sex and gender	This study is mainly focused on the development of integrated spatial multi-omics analysis method and the exploration of tumor metabolic remolding, so no gender-based analyses have been performed.
Population characteristics	For SM, SL, and ST samples, patients were diagnosed with gastric cancer. We have 7 male participants based on self-reported gender information. The age distribution is 50-89. All the tumor classification of these 7 patients is adenocarcinoma. And all patients did not undergo chemotherapy or radiotherapy before the surgery.
Recruitment	For AFADESI-MSI based SM and MALDI-MSI based SL analysis, and 10× Genomics Visium-based ST analysis, all patients were recruited from Peking Cancer Hospital (Beijing, China). There was no potential self selection bias.
Ethics oversight	Tissues samples were collected in concordance with the Declaration of Helsinki and Good Clinical Practice and has been approved by the Ethnical Committee of Peking Cancer Hospital (grant no. 2022KT87).

Note that full information on the approval of the study protocol must also be provided in the manuscript.

Field-specific reporting

Please select the one below that is the best fit for your research. If you are not sure, read the appropriate sections before making your selection.

☒ Life sciences ☐ Behavioural & social sciences ☐ Ecological, evolutionary & environmental sciences

For a reference copy of the document with all sections, see nature.com/documents/nr-reporting-summary-flat.pdf

Life sciences study design

All studies must disclose on these points even when the disclosure is negative.

Sample size	No sample size calculation was performed in this study. The sample size was determined by the purpose of this study. In this work, we focus more on how to perform spatially resolved multi-omics analysis and explore the cell-specific metabolic remodeling and interactions in gastric cancer. The realization of this exploration is based on how to extract and integrate transcriptomic, lipidomic and metabolomic data from highly heterogeneous tumor tissues, rather than on the size of the sample. Therefore, we analyzed gastric cancer tissues with the most significant spatial heterogeneity. A total of seven human gastric cancer tissue samples were collected and cut into 10 μm frozen sections. All these seven sections were subjected to AFADESI-MSI based SM and MALDI-MSI based SL analysis, and four of them were used to perform 10× Genomics Visium-based ST analysis.
Data exclusions	No data was excluded from the analysis.
Replication	Attempts at replication were successful. For Fig. 1c-e, the spatial images were built based on the scan spots, the number of scan spots were illustrated in these figures. For Fig. 4-6, The number stated in individual figure legends as n = x.
Randomization	Randomization was not applicable to this study, since we do not look at different groups of individuals subject to different treatment/intervention regiment.
Blinding	No blinding was done in this study since it is exploratory in character and have no elements that might be influenced by bias from the subject or observer.

Reporting for specific materials, systems and methods

We require information from authors about some types of materials, experimental systems and methods used in many studies. Here, indicate whether each material, system or method listed is relevant to your study. If you are not sure if a list item applies to your research, read the appropriate section before selecting a response.

Materials & experimental systems

n/a	Involved in the study
☐	☒ Antibodies
☒	☐ Eukaryotic cell lines
☒	☐ Palaeontology and archaeology
☐	☒ Animals and other organisms
☒	☐ Clinical data
☒	☐ Dual use research of concern

Methods

n/a	Involved in the study
☒	☐ ChIP-seq
☒	☐ Flow cytometry
☒	☐ MRI-based neuroimaging

Antibodies

Antibodies used

anti-CD20 (Abcam, monoclonal, ab78237),1:100
anti-CD38 (Abcam, monoclonal, ab108403),1:500
anti-AOC1 (Abcam, monoclonal, ab278497)1:500

Validation

Antibodies are first validated by manufactures where possible. CD20 (Abcam, ab78237) rabbit monoclonal antibody validated by manufacture via ICC/IF, WB, IP, IHC-P and evidences from the citing articles were also listed. e.g. on human spleen tissue (IHC), human tonsil tissue (IHC), human lymphoma tissue (IHC). CD30 (Abcam, ab108403) rabbit monoclonal antibody validated by manufacture via WB, IHC-P, Flow Cyt and evidences from the citing articles were also listed. e.g. on human tonsil tissue (IHC), human endometrial adenocarcinoma tissue (IHC), human colon tissue (IHC). AOC1 (Abcam, ab278497) rabbit monoclonal antibody validated by manufacture via WB, Flow Cyt (Intra), IHC-P and evidences from the citing articles were also listed. e.g. on human placenta tissue (IHC), human kidney tissue (IHC).

Animals and other research organisms

Policy information about studies involving animals; ARRIVE guidelines recommended for reporting animal research, and Sex and Gender in Research

Laboratory animals

A total of nine nude mouse were used to place xenografts in this study.

Wild animals

We did not use any wild animals.

Reporting on sex

This study is mainly focused on the development of integrated spatial multi-omics analysis method and the exploration of tumor metabolic remolding, so no gender-based analyses have been performed.

Field-collected samples

Studies did not include samples collected from the field.

Ethics oversight

The animal experiments were conducted with the approval of the Animal Ethical Committee at the Institute of Materia Medica, Chinese Academy of Medical Science, and Peking Union Medical College (grant no. 00007751).

Note that full information on the approval of the study protocol must also be provided in the manuscript.
